# Biosynthetic origin of anticancer dolastatin 15 reveals a noncanonical NRPS architecture enabling 2-keto acid activation

**DOI:** 10.1073/pnas.2615454123

**Published:** 2026-09-29

**Authors:** Manyun Chen, Jie Liu, Hsin-Ying Tsai, Cheng-Yu Li, Jeremiah D. Batucan, Campbell W. Eckhardt, Tam H. D. Pham, Emma K. Ellis, Ranjala Ratnayake, Valerie J. Paul, Steven D. Bruner, Mohamed S. Donia, Hendrik Luesch, Yousong Ding

**Affiliations:** ^a^ Department of Medicinal Chemistry, Center for Natural Products, Drug Discovery, and Development, University of Florida, Gainesville, FL 32610; ^b^ Department of Molecular Biology, Princeton University, Princeton, NJ 08544; ^c^ Department of Chemistry, University of Florida, Gainesville, FL 32611; ^d^ Smithsonian Marine Station, Fort Pierce, FL 34949; ^e^ Program in Cancer and Stem Cell Biology, Duke-National University of Singapore Medical School, Singapore 169857, Singapore

**Keywords:** natural product biosynthesis, genome-resolved metagenomics, nonribosomal peptide synthetase, enzyme evolution, dolastatin 15

## Abstract

Elucidating the biological origins and biosynthetic pathways of marine natural products remains a major challenge, particularly for compounds derived from microbial consortia. Here, we identify the marine cyanobacterial genus *Dapis* as the source of the potent anticancer natural product dolastatin 15 using genome-resolved metagenomics. Reconstruction of two complete and two near-complete genomes provides genome-resolved characterization of the genus *Dapis*, revealing extensive biosynthetic potential. Genome mining identified the dolastatin 15 biosynthetic gene cluster. Subsequent analyses reconstructed its pathway and uncovered a noncanonical five-domain nonribosomal peptide synthetase module containing an embedded 2-keto acid–activating domain, revealing an unexpected architectural solution for incorporating 2-keto acid-derived hydroxy acid building blocks. Comparative and evolutionary analyses support a model in which this architecture may have arisen through recruitment of keto acid-activating domains and reduction of canonical adenylation domain features. We further biochemically and structurally characterize an *O*-methyltransferase that catalyzes formation of the characteristic methoxy pyrrolinone moiety, thereby validating a key terminal tailoring step in the proposed biosynthetic pathway. Together, these findings define the biosynthetic logic of dolastatin 15 and expand understanding of substrate activation and assembly-line diversification in nonribosomal peptide biosynthesis.

## Significance

Marine natural products have inspired important medicines, yet for many compounds the producing organisms and biosynthetic genes remain unknown, limiting mechanistic understanding and biotechnological access. Here, we identify the marine cyanobacterial genus *Dapis* as the source of the clinically relevant anticancer compound dolastatin 15 and provide genomic characterization of this underexplored lineage. More broadly, this work uncovers a noncanonical nonribosomal peptide synthetase (NRPS) architecture for 2-keto acid activation, expanding current views of substrate activation in assembly-line biosynthesis. By linking a major drug scaffold to its biological origin and biosynthetic logic, this study provides a framework for connecting bioactive metabolites to their genetic sources and expanding opportunities for pathway discovery and engineering.

Marine organisms are a rich source of structurally diverse natural products, with more than 35,000 marine natural products (MNPs) reported to date (1–3). These compounds exhibit remarkable chemical novelty and biological activity (4, 5), as exemplified by multiple FDA-approved drugs derived from marine sources (6). However, a central challenge in MNP research is the inability to reliably link bioactive metabolites to their biosynthetic genes and producing organisms. This limitation is particularly pronounced in marine microbial systems, where organisms frequently exist within complex consortia that obscure the true origin of metabolites (7–10). Recent advances in genome-resolved metagenomics have expanded access to marine microbial diversity and biosynthetic potential. Large-scale sequencing efforts have generated thousands of reconstructed genomes and enabled the prediction of numerous biosynthetic pathways. However, these approaches often lack direct connections between predicted biosynthetic pathways and their corresponding metabolites (7, 11–13), highlighting a persistent gap between genomic potential and chemical function. As a result, for many clinically relevant MNPs, both the identity of the producing organism and the enzymatic logic underlying their assembly remain unresolved.

Cyanobacteria are among the most prolific producers of bioactive secondary metabolites, many of which are assembled by nonribosomal peptide synthetase (NRPS), polyketide synthase (PKS), and hybrid biosynthetic pathways and exhibit diverse pharmacological activities (14, 15). However, accurate assignment of these metabolites to their producing organisms has been hindered by morphology-based taxonomy, widespread misclassification, and the frequent occurrence of cyanobacteria in complex microbial consortia (16, 17). Recent advances in genome-resolved metagenomics enable the reconstruction of high-quality genomes from uncultivated microorganisms and the direct association of biosynthetic gene clusters (BGCs) with specific taxa (7, 11–13). These approaches provide a framework to link metabolites to their producers and to interrogate biosynthetic pathways at the molecular level (18, 19). Nevertheless, for many clinically important cyanobacterial metabolites, both the identity of the producing organism and the enzymatic logic underlying their assembly remain poorly defined. Consequently, uncertainty in taxonomy and microbial associations continue to impede compound reisolation, restrict the exploration of natural products for drug development, and limit their production through synthetic biology.

**Fig. 1.**
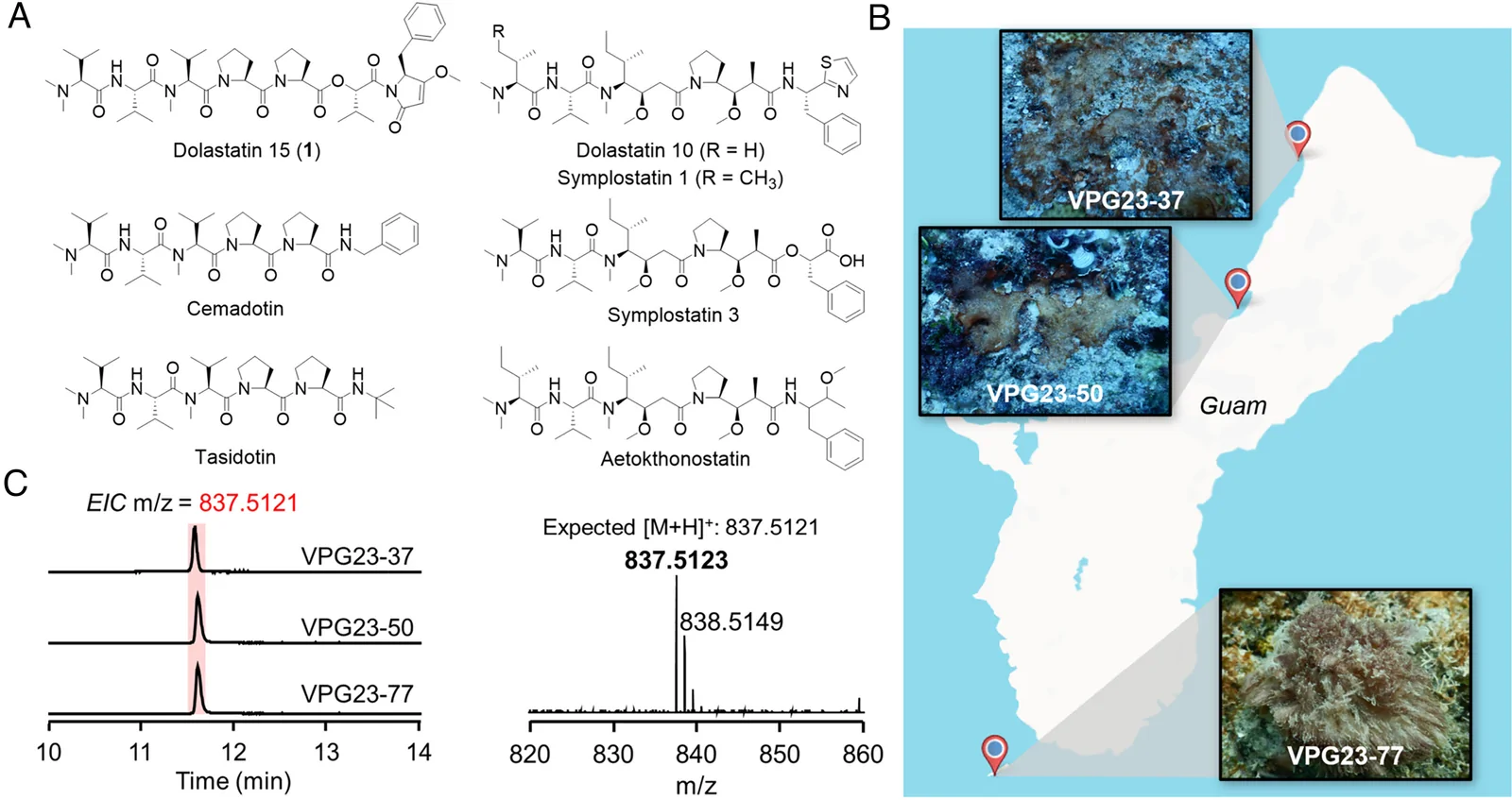
Identification of dolastatin 15 producing cyanobacteria. (*A*) Chemical structures of dolastatin 15, synthetic analogs, and selected natural analogs. (*B*) Geographical location of the *Dapis* sp. samples investigated. (*C*) Extracted ion chromatogram (EIC) traces and HRMS spectrum of dolastatin 15 identified in the cyanobacterial samples.

Dolastatins represent one of the most clinically impactful classes of cyanobacterial natural products (20). The tubulin-targeting compound dolastatin 10 (Fig. 1*A*) exhibits exceptional cytotoxicity (21), and its synthetic analogs monomethyl auristatins E (MMAE) and F (MMAF) have served as the cytotoxic payloads of antibody–drug conjugates (ADCs), resulting in six FDA-approved medications (15). More recently, trastuzumab botidotin, a HER2-targeted ADC carrying the MMAF derivative duostatin 5, was approved for cancer treatment in China. Dolastatin 15 (Fig. 1*A*) is a closely related analog with highly potent nanomolar cytotoxicity (22) along with strong potential for therapeutic development, as reflected by the synthetic derivatives tasidotin and cemadotin (Fig. 1*A*), which have advanced to clinical evaluation as ADC payloads for solid tumor treatment (23, 24). Although dolastatins were originally isolated from the sea hare *Dolabella auricularia* (22, 25), our subsequent studies established marine cyanobacteria as the native producers, and the dolastatin 10 producer was recently classified as *Caldora penicillata* (26–28), highlighting the challenges of assigning natural products to their biological sources. More recently, we identified the dolastatin 10 BGC using metagenomic approaches (19), providing biosynthetic insights into this compound family. We also isolated dolastatin 15 from a marine cyanobacterial sample (28). However, in contrast to dolastatin 10, the biosynthetic origin and enzymatic logic underlying the assembly of its structural analog dolastatin 15 remain unresolved.

Here, we combine genome-resolved metagenomics, comparative genomics, and biochemical, bioinformatic, and structural analyses to identify the cyanobacterial producer of dolastatin 15 and elucidate its biosynthetic pathway. We show that the natural product is produced by members of the marine cyanobacterial genus *Dapis*, a genomically underexplored lineage (29), and provide comprehensive genomic insight into its biosynthetic potential. Genome mining enabled identification of the dolastatin 15 BGC and reconstruction of its pathway. Subsequent analyses uncover a noncanonical five-domain NRPS architecture containing an embedded 2-keto acid-activating domain and validate a key tailoring step in dolastatin 15 assembly. Together, these findings resolve the biological origin of dolastatin 15 and reveal insights into depsipeptide biosynthesis. This work further establishes a framework for linking natural products to their biosynthetic genes and producers in complex microbial systems, enabling access to the vast chemical diversity encoded in uncultivated microbial lineages.

## Results

### Identification of Dolastatin 15-Producing *Dapis* sp.

We previously reported the direct isolation of dolastatin 15 from a benthic marine cyanobacterium from Guam (28), supporting its cyanobacterial origin. To further investigate its biosynthesis and identify the producing organisms, cyanobacterial samples collected from multiple sites on Guam were screened for dolastatin 15 production by LC-HRMS analysis (Fig. 1*B*). Dolastatin 15 was detected in crude extracts of three samples (VPG23-37, VPG23-50, and VPG23-77) (Fig. 1*C*), and its identity was confirmed by isolation followed by LC-HRMS and ^1^H NMR analysis (*SI Appendix*, Fig. S1). These dolastatin 15–producing samples were subjected to both Illumina short-read and PacBio HiFi long-read sequencing, yielding four high-quality metagenome-assembled genomes (MAGs) (Dataset S1). Two MAGs, hereafter referred to as *Dapis* sp. VPG23-77 and VPG23-50, represent complete circular genomes, whereas the two other MAGs, hereafter referred to as *Dapis* sp. VPG23-37-b1 and -b2, consist of two and three contigs, respectively, with 100% completeness and low contamination (1.11% and 1.42%).

**Fig. 2.**
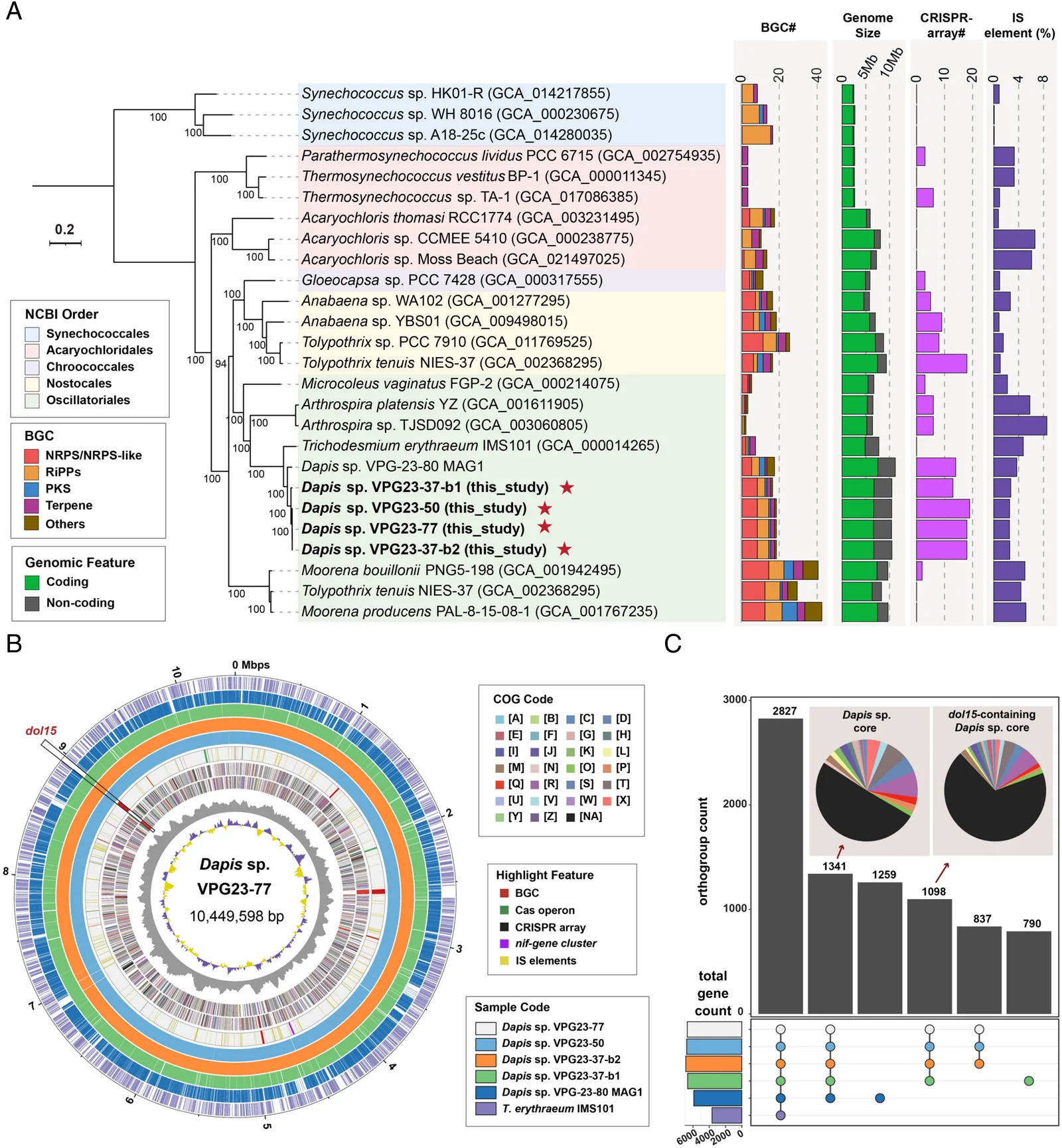
Genomic analysis of the dolastatin 15-producing *Dapis* sp. (*A*) Phylogenomic analyses of *Dapis* sp. strains and cyanobacteria reference genomes and highlighted genomic features. Phylogenetic tree was constructed using 568 concatenated single-copy genes identified from these genomes using OrthoFinder with 1,000 bootstraps. *Dapis* strains from this study are marked with red stars. (*B*) Circular genome of *Dapis* sp. VPG23-77 with highlighted genomic features and whole genome alignments of related genomes. Concentric rings (from inside to outside) indicate: 1, GC skew; 2, GC contents; 3, protein coding genes on forward frame; 4, protein coding genes on reverse frame; 5, *Dapis* sp. VPG23-77 genome with highlighted features; 6 to 10, whole genome alignments of related genomes against *Dapis* sp. VPG23-77. Only regions with ≥80% alignment identity are shown, and gaps indicate the absent or highly divergent regions. Protein coding genes in ring 3 and ring 4 were colored based on their Cluster of Orthologous Group (COG) categories based on the Integrated Microbial Genome platform (IMG) annotation ([A], RNA processing and modification; [B], chromatin structure and dynamics; [C], energy production and conversion; [D], cell cycle control, cell division, and chromosome partitioning; [E], amino acid transport and metabolism; [F], nucleotide transport and metabolism; [G], carbohydrate transport and metabolism; [H], coenzyme transport and metabolism; [I], lipid transport and metabolism; [J], translation, ribosomal structure, and biogenesis; [K], transcription; [L], replication, recombination, and repair; [M], cell wall/membrane/envelope biogenesis; [N], cell motility; [O], posttranslational modification, protein turnover, and chaperones; [P], inorganic ion transport and metabolism; [Q], secondary metabolites biosynthesis, transport, and catabolism; [R], general function prediction only; [S], function unknown; [T], signal transduction mechanisms; [U], intracellular trafficking, secretion, and vesicular transport; [V], defense mechanisms; [W], extracellular structures; [X], mobilome, prophages, and transposons; [Y], nuclear structure; [Z], cytoskeleton; [NA], not assigned). (*C*) Orthogroup analyses of five *Dapis* genomes and *T. erythraeum* IMS101. UpSet plot shows the six most abundant orthogroup intersections among genomes. Pie plots show the COG assignment of *Dapis* sp. VPG23-77 genes belonging to five *Dapis* core orthogroups and four *dol*-15 containing *Dapis* core orthogroups.

Due to the lack of a validated *Dapis* reference genome in Genome Taxonomy Database (GTDB), genome-based taxonomic classification assigned these dolastatin 15–producing MAGs to *Trichodesmium*, the closest available reference genus, based on phylogenomic placement rather than average nucleotide identity (ANI) to a *Dapis* reference genome. These MAGs share only ~86% ANI with the representative strain *Trichodesmium erythraeum* IMS101, indicating substantial genomic divergence. To resolve this discrepancy, we performed a genome-scale phylogenomic analysis incorporating recently reported *Dapis* MAGs (30), together with phylogenetic analysis based on 16S rRNA genes. Both analyses consistently support assignment of these strains to the genus *Dapis* (family Microcoleaceae) (Fig. 2*A*, *SI Appendix*, Figs. S2 and S3, and Dataset S2). The cyanobacterial genus *Dapis* was established in 2018 following its separation from *Lyngbya* (29). Although five species have been described to date (29, 30), the genus remains genomically underexplored. The four MAGs reported here form a distinct clade within *Dapis* based on 16S rRNA phylogeny (*SI Appendix*, Fig. S2) and are clearly separated from previously available *Dapis* genomes in phylogenomic analysis (*SI Appendix*, Fig. S3), sharing ~90.4 to 90.5% ANI with their closest relative, *Dapis* sp. VPG23-80 MAG1 (31). Among the strains identified in this study, three (*Dapis* sp. VPG23-77, VPG23-50, and VPG23-37-b2) are highly similar (>99% ANI), whereas *Dapis* sp. VPG23-37-b1 is comparatively more divergent (~96% ANI relative to the other strains).

Several bioactive secondary metabolites, e.g., iheyamides, malyngolide, lyngbic acid, lyngbyoic acid, and dapalides A-C, have been isolated from *Dapis* spp. (29, 31, 32). However, exploration of their genomic features and biosynthetic potential has been limited by the lack of high-quality *Dapis* genomes. Here, we report complete and high-quality MAGs for this genus, enabling comprehensive analysis of biosynthetic gene content and other genomic features. Given the overall high similarity among the four *Dapis* MAGs, *Dapis* sp. VPG23-77 was selected as the representative genome for subsequent comparative analyses.

### Genomic Characterization and Comparison of Dolastatin 15-Producing *Dapis* sp.

The *Dapis* sp. VPG23-77 genome is 10.45 Mb with a GC content of 36% and encodes 8,170 predicted proteins, of which 4,312 are assigned Cluster of Orthologous Groups (COG) functions (Dataset S2). Two copies of the 16S rRNA gene are present, sharing 99.3% sequence identity, a feature conserved across the four *Dapis* MAGs identified in this work. These genomes exhibit unusually low coding density (average 63.7% versus 83.7% in reference cyanobacteria), consistent with increased noncoding content (Fig. 2*A*), a feature also observed in *Trichodesmium* strains (33). All four *Dapis* genomes encode a complete nitrogen fixation gene cluster, including *nifB, nifS, nifU, nifH, nifD, nifK, nifE*, and *nifX*, suggesting the capacity for diazotrophic growth similar to *Trichodesmium* (Fig. 2*B*) (34). In addition, all genomes contain type I-D and type III-B CRISPR-Cas systems and associated repeat-spacer arrays, consistent with adaptive immunity against mobile genetic elements (35). Moderate levels of insertion sequence elements (2.53 to 2.74%) were observed (Fig. 2*A*), lower than those in *Dapis* sp. VPG23-80 MAG1 and *T. erythraeum* IMS101.

Comparative genomic analysis of the four dolastatin 15–producing *Dapis* MAGs, *Dapis* sp. VPG23-80 MAG1, and *T. erythraeum* IMS101 revealed 2,827 core orthogroups shared across all genomes (Fig. 2*B* and Dataset S3), representing 43% of coding genes in *Dapis* sp. VPG23-77 but 75% in *T. erythraeum*. Within the genus, 1,341 orthogroups are conserved across all five *Dapis* genomes, whereas a substantial fraction of genes are lineage-specific, indicating pronounced genomic diversification. Among *Dapis* strains, the VPG23-80 MAG1 is the most divergent, containing 1,259 unique orthogroups enriched in mobilome- and regulatory-associated functions. In contrast, the dolastatin 15–producing *Dapis* MAGs share a distinct set of orthogroups enriched in signal transduction pathways. Consistent with its slightly lower ANI (~96%), *Dapis* sp. VPG23-37-b1 also contains a substantial number of unique genes relative to the other strains.

Together, these results indicate that *Dapis* genomes are both expanded and highly dynamic, sharing less than half of their coding genes with *Trichodesmium* and exhibiting substantial diversification within the genus. This genomic complexity, coupled with their enriched biosynthetic capacity as shown below, underscores *Dapis* as a promising and previously underexplored lineage for natural product discovery.

### BGC Analysis of Dolastatin 15–Producing *Dapis* sp.

**Fig. 3.**
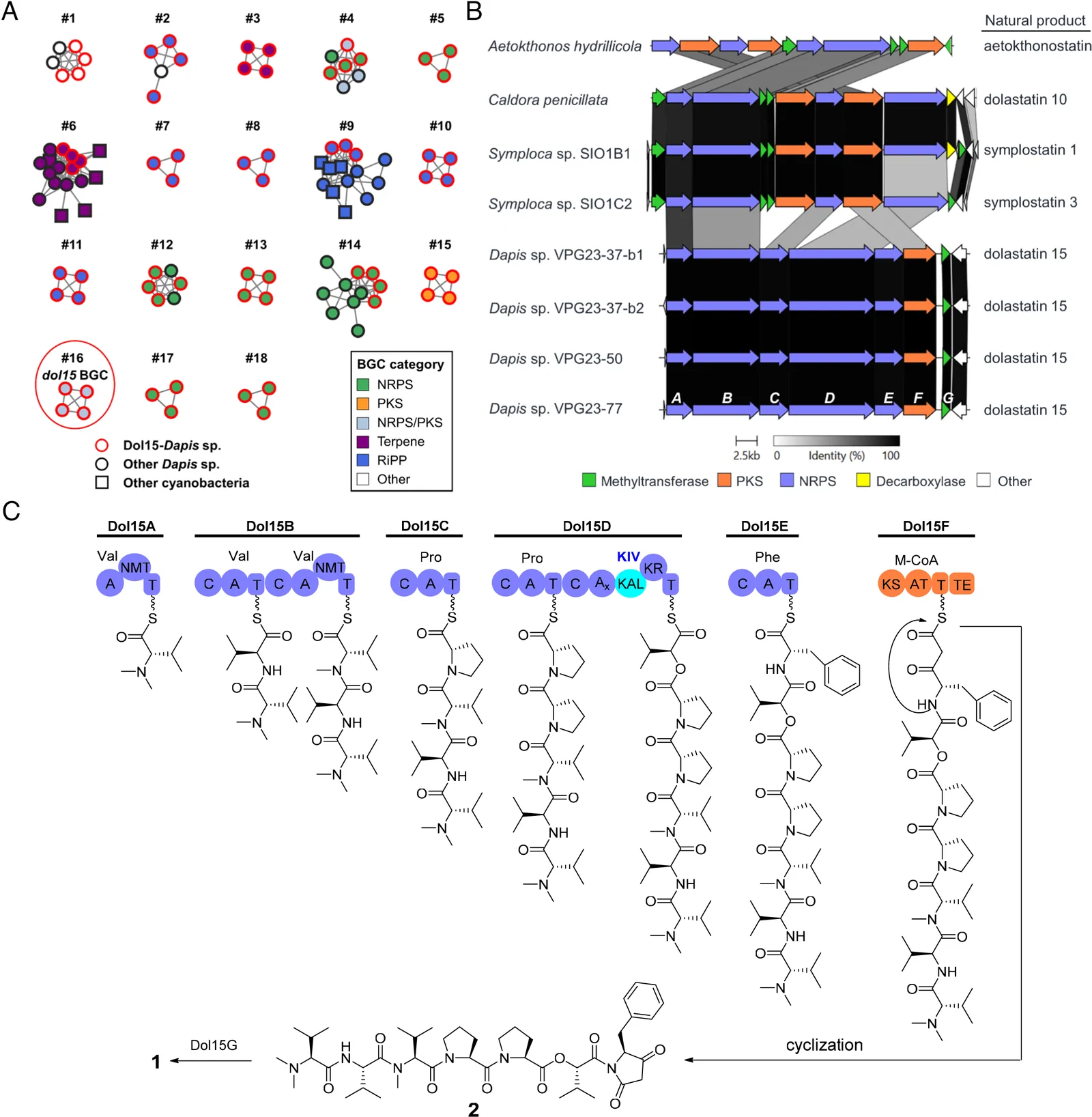
Identification of the *dol15* BGC. (*A*) BGC similarity network of dolastatin 15 producing *Dapis sp*. The BGC clusters were generated by BiG-SCAPE with a cutoff threshold at 0.3 and the clusters were numbered according to the BGC annotation table (*SI Appendix*, Table S1). (*B*) Comparison of the *dol15* BGC with other dolastatin-type BGCs. (*C*) Proposed biosynthetic pathway of dolastatin 15. C: condensation; A: adenylation; A_x_: inactive adenylation domain in ketoacid activating NRPS module; KAL: 2-keto acid AMP ligase; NMT: *N-*methyltransferase; T: thiolation; KR: ketoreductase; KS: ketosynthase; AT: acyltransferase; TE: thioesterase; M-CoA: malonyl-CoA; KIV: 2-ketoisovaleric acid.

Consistent with their expanded genomes, *Dapis* strains harbor a diverse repertoire of BGCs. antiSMASH prediction (36) revealed 18 BGCs in *Dapis* sp. VPG23-77, including two terpenes, six ribosomally synthesized and posttranslationally modified peptides (RiPPs), six NRPSs, one PKS, and two NRPS/PKS hybrid clusters (*SI Appendix*, Table S1). To assess the novelty and conservation of *Dapis* BGCs, we curated a dataset comprising BGCs from four dolastatin 15–producing *Dapis* strains, previously reported *Dapis* MAGs of limited completeness and quality (*SI Appendix*, Fig. S3), and 385 complete cyanobacterial genomes from the NCBI RefSeq database (March, 2026). These BGCs were grouped using BiG-SCAPE (37) with a similarity threshold of 0.3. The resulting network shows that, with the exception of terpene BGC#6 and RiPP BGC#9, the majority of BGCs in *Dapis* sp. VPG23-77 are restricted to the *Dapis* genus, with 11 clusters uniquely present in dolastatin 15–producing strains (Fig. 3*A*). This pattern suggests that *Dapis* represents a distinct but largely unexplored genus for MNP research (30). A subset of BGCs is predicted to encode conserved cyanobacterial metabolites. For example, BGC#4 corresponds to a cyanochelin-like siderophore pathway (38), and BGC#14 encodes a mycosporine-like amino acid pathway containing both MysE and MysD (39). Consistent with comparative genomic analysis indicating that *Dapis* sp. VPG23-37-b1 is somewhat divergent from the other dolastatin 15–producing strains, this genome lacks six BGCs (#5, 7, 8, 9, 17, and 18) that are conserved in the others (Fig. 3*A* and *SI Appendix*, Fig. S4).

Based on gene annotation and NRPS/PKS specificity predictions, BGC#16 was identified as the dolastatin 15 BGC (*dol15*) (GenBank accession: PZ353175) (40). The 34-kb BGC encodes seven core biosynthetic enzymes, including five NRPSs (Dol15A-E), one PKS (Dol15F), and one standalone *O*-methyltransferase (Dol15G) (Fig. 3*B* and *SI Appendix*, Table S2). The substrate specificities of most NRPS adenylation (A) domains agree well with the structure of dolastatin 15 (Fig. 3*C* and *SI Appendix*, Table S3). Dol15A, a single NRPS loading module, contains an embedded *N*-methyltransferase (MT) domain predicted to catalyze the dimethylation of loaded l-Val. Dol15B is a bimodular NRPS responsible for incorporation of the l-Val-*N*-methyl-l-Val moiety. Subsequently, two l-Pro building blocks are incorporated by Dol15C and the first NRPS module of Dol15D. The second NRPS module of Dol15D is predicted to incorporate (2*S*)-hydroxyisovaleric acid (HIVA). Following incorporation of the final amino acid, l-Phe, by Dol15E, the PKS Dol15F extends the peptide chain using malonyl-CoA as a substrate. The thioesterase (TE) domain of Dol15F is likely responsible for cyclization to form the pyrrolidine-2,4-dione moiety and release of the dolastatin 15 precursor (**2**). Finally, the tailoring enzyme Dol15G catalyzes *O*-methylation to generate the mature dolastatin 15 (**1**).

The identification of the *dol15* BGC expands the known biosynthetic logic of the dolastatin family (e.g., dolastatin 10 and aetokthonostatin), whose gene clusters occur across diverse marine and freshwater cyanobacteria (Fig. 3*B*) (19, 41). Relative to the previously characterized dolastatin 10 and aetokthonostatin pathways, the *dol15* BGC contains fewer MT genes, mirroring the reduced methylation pattern of dolastatin 15 (Fig. 1), which lacks the highly methylated residues (*3R*,4*S*,5*S*)-dolaisoleuine (Dil) and (2*R*,3*R*,4*S*)-dolaproine (Dap) that demand extensive tailoring modifications.

### A Noncanonical Five-Domain NRPS Module Enables 2-Keto Acid Activation.

**Fig. 4.**
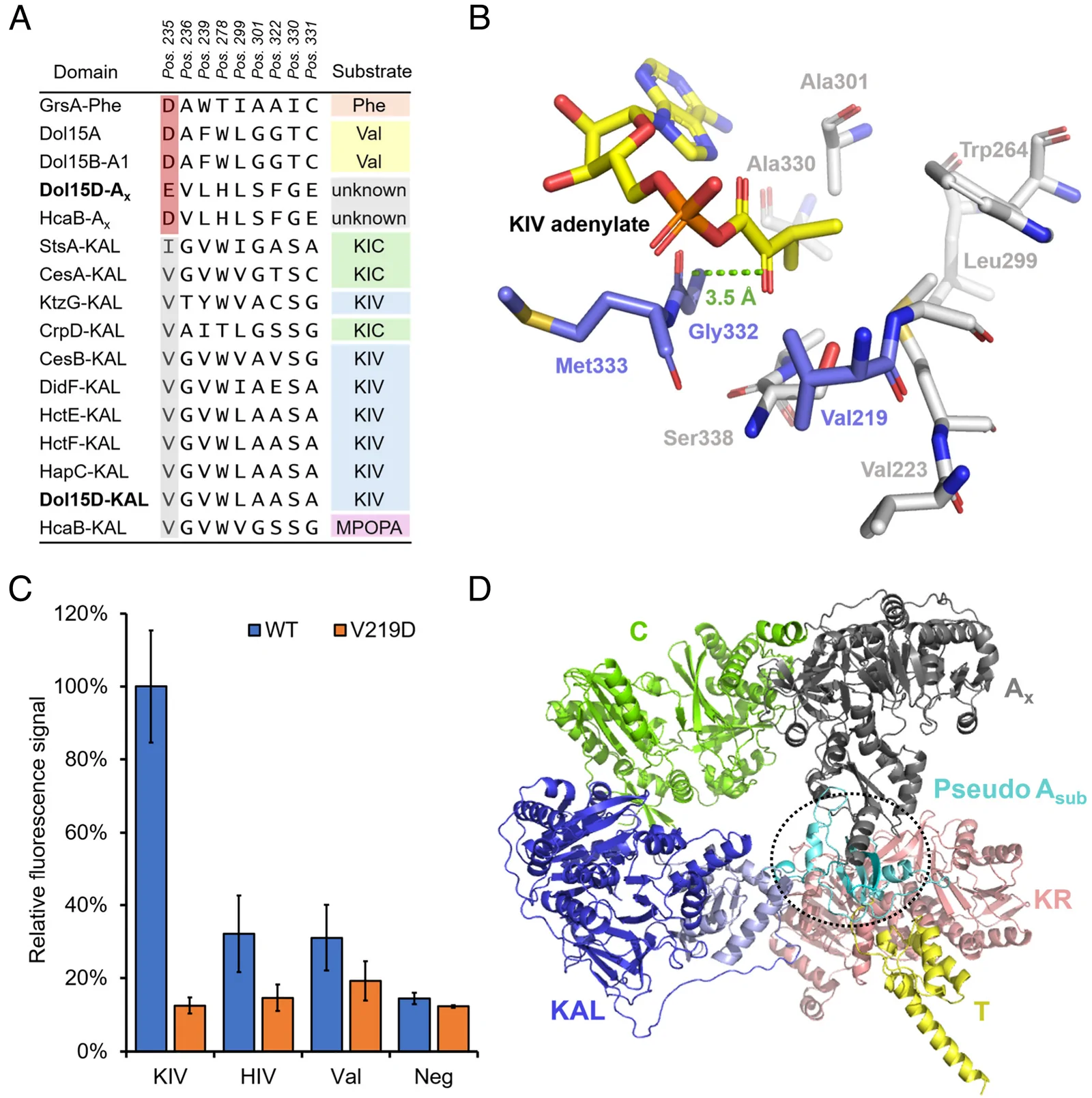
Sequence analysis and biochemical characterization of the Dol15D-M2 KAL domain. (*A*) Alignment of the substrate specificity codes of A_x_ and KAL domains from Dol15D-M2 and selected NRPS KAL utilizing 2-keto acids. KIC: 2-ketoisocaproic acid; KIV: 2-ketoisovaleric acid; MPOPA: 3-(4-methoxyphenyl)-2-oxopropanoic acid. (*B*) Binding model of Dol15D-M2 KAL with the KIV-adenylate intermediate predicted by AlphaFold 3. (*C*) In vitro characterization of the substrate specificity of the WT and V219D variant of the Dol15D-M2 KAL domain. The adenylation activity was determined by the measurement of fluorescence-based method to quantify pyrophosphate generated in the reaction. At least four replicates were performed for each group. (*D*) Predicted structure of Dol15D-M2 by AlphaFold 3, with a helical element from the A_x_ domain contributing to the pseudo A_sub_ domain of the KR domain.

Depsipeptide biosynthesis in systems such as cryptophycin and cereulide relies on activation of 2-keto acids by specialized adenylation (A_k_) domains (42, 43), followed by ketoreduction to generate hydroxy acid units that form ester linkages. Canonical NRPS A domains contain a conserved Asp235 residue required for amino acid recognition (44), whereas A_k_ domains replace this residue with a hydrophobic residue to accommodate keto acid substrates (Fig. 4*A*). Based on the structure of dolastatin 15 (Fig. 1*A*), the second module of Dol15D (Dol15D-M2) would be expected to activate 2-ketoisovaleric acid (KIV). However, antiSMASH 8.0 (36) analysis revealed a noncanonical five-domain architecture (C–A–CAL–KR–T) (*SI Appendix*, Fig. S5), in which the A domain (hereafter A_x_) retains an acidic residue (Glu235) at position 235 and is therefore unlikely to activate a keto acid substrate (Fig. 4*A*). Although A_x_ retains the canonical A1-A10 A domain architecture (45), the A3 motif is only partially conserved (*SI Appendix*, Fig. S6), consistent with impaired ATP binding and loss of catalytic function. In contrast, the embedded CAL (CoA ligase) domain annotated by antiSMASH lacks the Asp235-equivalent residue and contains sequence features consistent with keto acid recognition (Fig. 4*A*). Classical CAL belongs to the adenylate-forming enzyme (ANL) superfamily and catalyzes ATP-dependent activation of carboxylates via acyl-AMP intermediates (46, 47). However, because the designation CAL implies a classical CoA ligase, whereas both the annotated A_k_ and CAL domains are predicted to catalyze ATP-dependent activation of 2-keto acid substrates, we refer to them collectively as 2-keto acid AMP ligases (KALs) throughout this study.

We next characterized the function of the Dol15D-M2 KAL domain. This domain shares moderate sequence similarity to the 2-ketoisocaproic acid (KIC)-specific KAL domain of StsA (45% identity, 62% similarity), a cereulide synthetase homolog from *Bacillus stratosphericus* LAMA 585 (48). Recently, the X-ray structure of its initiation module carrying a KAL-KR didomain has been reported (48). Consistent with sequence similarity, structural prediction shows strong resemblance to the StsA KAL domain (PDB: 6ULX; Cα rmsd = 0.99 Å), particularly within the conserved A_core_ region (*SI Appendix*, Fig. S7). Modeling of a KIV-adenylate intermediate positions the 2-keto carbonyl proximal to Val219 in Dol15D-M2 KAL (Fig. 4*B*). A conserved Gly-Met motif, implicated in 2-keto recognition in the StsA KAL domain, is retained, with the backbone carbonyl between Gly332 and Met333 located ~3.5 Å from the KIV 2-keto group (Fig. 4*B* and *SI Appendix*, Fig. S8), consistent with a stabilizing carbonyl–carbonyl interaction. Biochemical assays confirm this prediction. The purified KAL domain catalyzes ATP-dependent adenylation of KIV, whereas a Val219Asp variant abolishes activity (Fig. 4*C*). Despite multiple attempts, the A_x_ domain of Dol15D-M2 could not be obtained in soluble form. Together, these results support a model in which the KAL domain, rather than the A_x_ domain, mediates 2-keto acid activation. The resulting ketoacyl intermediate is subsequently reduced by the KR domain to generate a hydroxyacyl building block for incorporation into the growing peptide chain through ester bond formation (Fig. 3 and *SI Appendix*, Fig. S9*A*).

Beyond catalysis, structural comparisons suggest that the A_x_ domain may retain a supporting structural role. In the StsA KAL–KR structure (48), a small domain appended to KR adopts a pseudo A_sub_-like fold that is completed by structural elements provided from the N terminus. Consistently, AlphaFold 3 models of Dol15D-M2 predict that the A_x_ domain may contribute a helical element that could help complete this pseudo A_sub_-like fold appended to its KR domain (Fig. 4*D*), supporting a potential structural role in maintaining module architecture. Thus, Dol15D-M2 represents a noncanonical assembly line in which keto acid activation is decoupled from the canonical A domain and instead mediated by an embedded KAL domain.

### Evolution and Diversification of Keto Acid-Activating NRPS Modules.

**Fig. 5.**
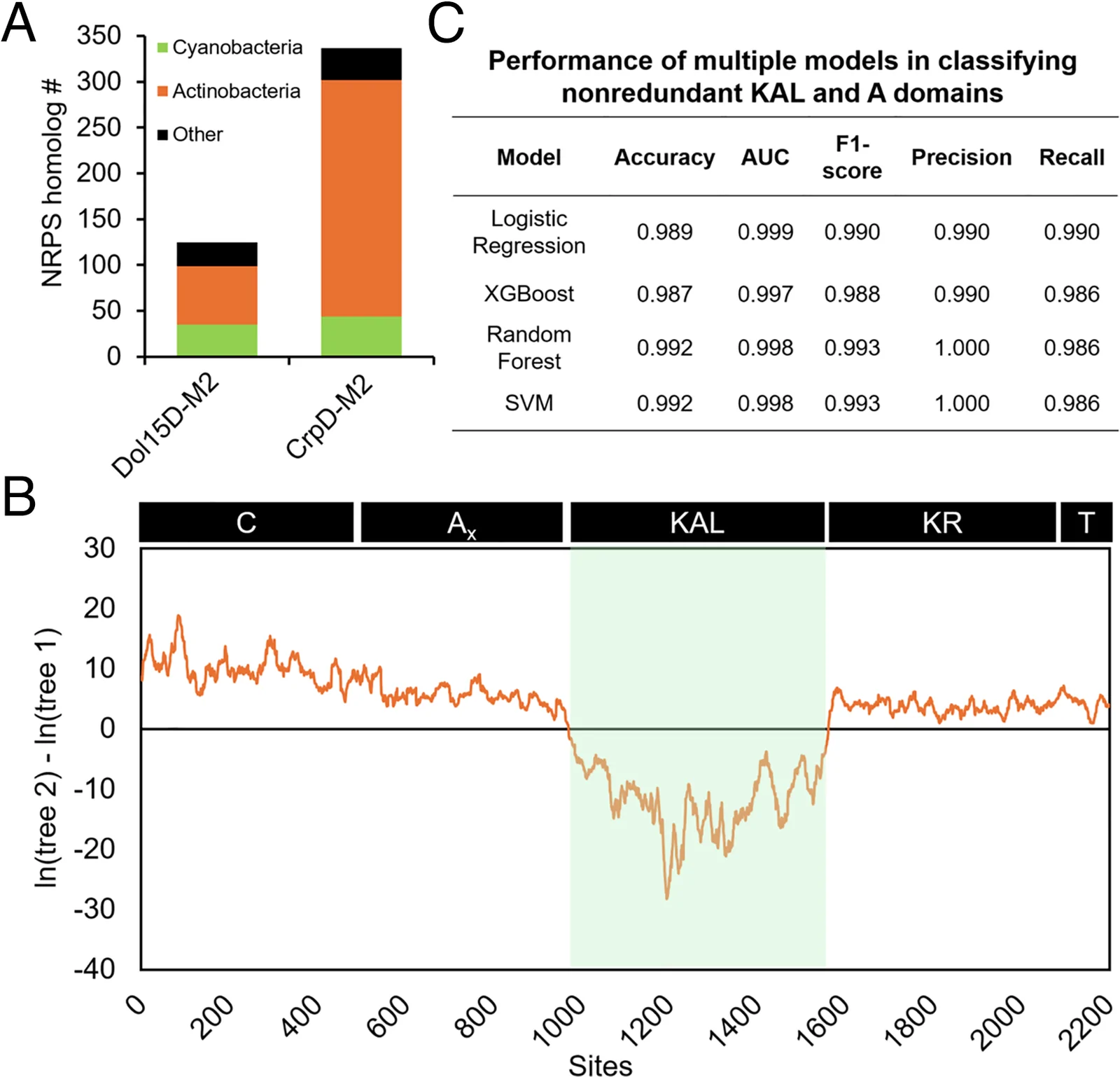
Evolution analysis of keto acid-activating NRPS modules. (*A*) Distribution of 125 five-domain Dol15-M2 homologs and 337 four-domain CrpD-M2 homologs in different bacterial phyla. (*B*) Comparative phylogenetic analysis of 125 Dol15-M2 homologs using a two-tree model reveals recombination insertion of a KAL domain between the C-A_x_ and KR-T segments. (*C*) Four supervised machine learning classifiers distinguish KAL and canonical A domains using ESM2-derived embeddings.

To assess how widespread keto acid–activating NRPS architectures are, we identified 125 Dol15D-M2 homologs with the same five-domain organization and 337 homologs of the related four-domain module CrpD-M2 in the NCBI RefSeq and antiSMASH database version 5 (49). CrpD-M2, a validated keto acid-activating NRPS module with a C-KAL-KR-T architecture, activates KIC during the biosynthesis of the cyanobacterial depsipeptide cryptophycin (43, 50). These modules are distributed primarily in actinobacteria, followed by cyanobacteria (Fig. 5*A*). Although Dol15D-M2 homologs are generally larger because they retain an A_x_ domain (*SI Appendix*, Fig. S10), the extent of A_x_ degeneration varies substantially, and many four-domain homologs also retain partial A_x_-like regions that are not recognized by antiSMASH, suggesting a continuum of domain reduction rather than two discrete architectures. Notable Dol15D-M2 homologs include modules involved in the biosynthesis of hectoramide B (HcaB), hectochlorin (HctF-M1), hapalosin (HapC), and pyridomycin (PyrG) (*SI Appendix*, Fig. S11) (51–54). PyrG represents the only five-domain NRPS example for which the CAL/A_k_ domain has been experimentally shown to activate 2-keto-β-methylvaleric acid (51, 55), and its accompanying A_x_ domain likewise lacks an intact A3 motif. Notably, A_x_ domains across Dol15D-M2 homologs contain similarly divergent or partially conserved A3 motifs (*SI Appendix*, Fig. S6), consistent with a shared pattern of catalytic attenuation in this architecture. AlphaFold3 modeling further revealed that CrpD-M2 retains a partial A_x_-like region not predicted by antiSMASH (*SI Appendix*, Fig. S12), positioned similarly to the A_x_ domain in Dol15D-M2 and N-terminal structural elements of StsA (48), suggesting that the four-domain architecture may have arisen through reduction of a five-domain precursor. Consistent with this model, sequence alignments show that these partial A_x_-like regions retain A6-A10 motifs (*SI Appendix*, Fig. S6).

Phylogenetic and recombination analyses provide insights into the evolution of keto acid-activating NRPSs. Evolutionary analysis using a hidden Markov model framework (56) to infer recombination breakpoints under two- or three-tree models identified breakpoints consistent with insertion of a KAL domain between the C-A_x_ and KR-T segments in Dol15D-M2 homologs (Fig. 5*B* and *SI Appendix*, Fig. S13*A*). The same recombination breakpoints were detected among CrpD-M2 homologs using the three-tree model (*SI Appendix*, Fig. S13*B*). Together, these results support a model in which these architectures may have arisen through KAL recruitment and functional degeneration of A_x_.

We next examined how these KAL domains relate to other adenylating enzymes. Phylogenetic analysis places them in a distinct clade within the ANL superfamily, separate from fatty acyl-CoA ligases and canonical NRPS A domains (*SI Appendix*, Fig. S14), yet closely related to experimentally validated KAL domains. We next examined whether KAL domains are distinguishable from canonical NRPS A domains based on sequence features alone. Using a nonredundant dataset (≤85% sequence identity) comprising 219 KAL [84 A_k_ and 135 CAL annotated by antiSMASH 8.0 (36)] and a balanced reference set of 169 canonical A domains, we analyzed domain relationships using pretrained ESM2 (57) protein language model embeddings together with supervised machine learning classifiers, including logistic regression, support vector machines (SVMs), random forests, and XGBoost (58). Principal component analysis (PCA) and uniform manifold approximation and projection (UMAP) of the ESM2 embeddings revealed clear separation between KAL and canonical A domains (*SI Appendix*, Fig. S15). Consistent with these visualizations, all four classifiers achieved excellent performance, with accuracies ranging from 98.7 to 99.2% and AUC values of 0.997 to 0.999, demonstrating that KAL domains constitute a sequence-distinct class readily distinguishable from canonical NRPS A domains (36, 57) (Fig. 5*C*).

Finally, clustering of KAL domains with characterized enzymes suggests grouping according to substrate preference. Domains predicted to activate aliphatic keto acids such as KIV and KIC cluster separately from those predicted to accept aromatic keto acids such as phenylpyruvic acid (PPA) or 4-hydroxyphenylpyruvic acid (HPPA) (*SI Appendix*, Fig. S16). This framework provides a basis for functional prediction of uncharacterized pathways. For example, a *dol15*-like BGC in *Symploca* sp. SIO1C2 (Figs. 1*A* and 3*B*) is predicted to incorporate an aromatic keto acid and may produce symplostatin 3 (*SI Appendix*, Figs. S16 and S17), a cytotoxic dolastatin analog whose biosynthesis has not been previously resolved (59). Collectively, these findings suggest that KAL domains represent a conserved and adaptable strategy for expanding NRPS substrate scope.

### Dol15G Validates the Proposed Terminal Tailoring Step in Dolastatin 15 Biosynthesis.

**Fig. 6.**
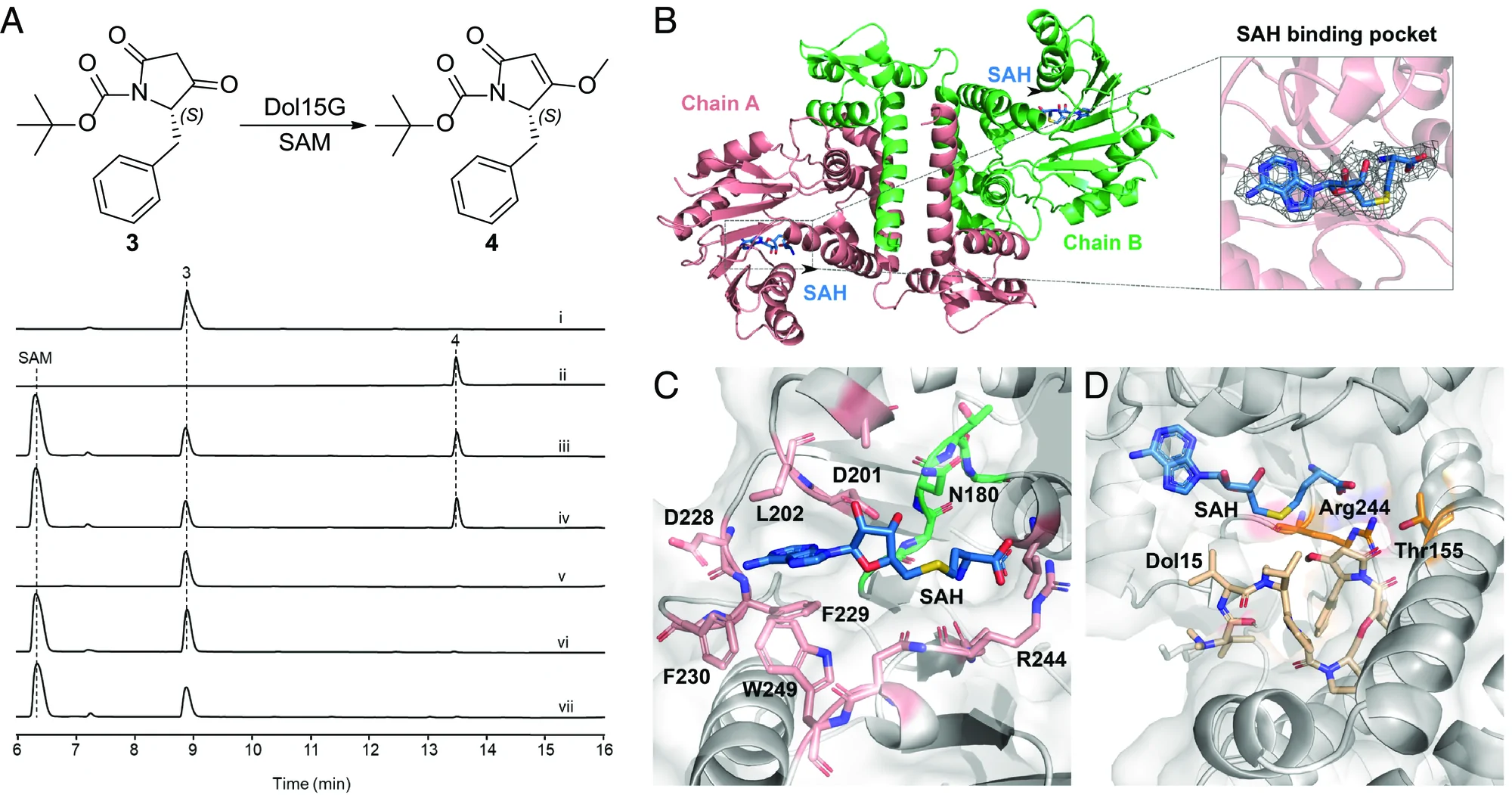
Biochemical characterization of Dol15G. (*A*) Scheme of the in vitro Dol15G reaction and LC traces of the in vitro Dol15G reactions underlying its activity, cofactor dependence, and substrate selectivity. Traces represent: (i) synthetic substrate mimic **3**; (ii) synthetic standard compound **4**; (iii) full Dol15G reaction containing 10 mM MgCl_2_, 0.2 mM **3**, 2 mM SAM, and 2 µM Dol15G in 50 mM HEPES buffer at pH 7.5; (iv) the reaction without MgCl_2_ and with the addition of 1 mM EDTA; (v) reaction without SAM; (vi) negative control using heat deactivated Dol15G; (vii) reaction with **3R** as substrate. (*B*) Overall structure of Dol15G in complex with SAH. Two protomers are shown in pink and green, respectively. SAH within the structure is shown in blue. A 2Fo-Fc map (*Inset*, contoured at 1.0 σ) around SAH is shown. (*C*) Dol15G SAH binding site. Residues that participate in hydrophobic interaction and hydrogen bond interaction are shown in pink, and the glycine-rich motif is shown in green. (*D*) Dolastatin 15 is docked into active site of Dol15G. The key residues involved in the substrate specificity are shown in orange and dolastatin 15 is shown in beige.

Dolastatin 15 contains a characteristic C-terminal *N*-acylated tetramic acid (pyrrolidine-2,4-dione) moiety, a structural motif widely found in natural products (60, 61). *O*-methylated tetramic acids are particularly enriched in marine cyanobacterial metabolites, including dolastatin analogs such as caldoramide (62) and symplostatin 4 (63) (*SI Appendix*, Fig. S18), yet the biosynthetic basis of this modification remains poorly understood. To complete experimental validation of the proposed dolastatin 15 pathway (Fig. 3*C*) and define the molecular basis of this terminal tailoring step, we expressed recombinant Dol15G in *Escherichia coli* (*SI Appendix*, Fig. S19). Because chemical synthesis of the predicted dolastatin 15 precursor **2** has not been established, we prepared a simplified substrate mimic (**3**) along with its *O*-methylated product (**4**) as a standard for in vitro assays (*SI Appendix*, Figs. S20–S22). HPLC analysis showed that Dol15G efficiently converts **3** to a product with identical retention time to **4**, whereas no product was detected in control reactions lacking enzyme or SAM (Fig. 6*A*). The reaction proceeds in the absence of divalent metal ion Mg^2+^ and is unaffected by EDTA, indicating that Dol15G functions as a metal-independent MT. To assess stereoselectivity, we synthesized the enantiomeric substrate (**3*R***) from d-Phe. Dol15G does not methylate **3*R***, demonstrating strict stereoselectivity.

To provide structural insight into this transformation, we determined the crystal structure of Dol15G in complex with *S*-adenosylhomocysteine (SAH) at 2.65 Å resolution (space group C121; PDB: 10QU) (64) (*SI Appendix*, Table S4). Dol15G adopts a canonical class I MT fold and forms a homodimer, with N-terminal α-helices contributing to the dimer interface (Fig. 6*B*) (65, 66). The electron density map clearly shows SAH present in the cocrystal structure (Fig. 6 *B*, *Inset*). The adenine moiety is stabilized by a hydrophobic pocket formed by Leu202, Phe229, Phe230, and Trp249, while Asp228 forms a hydrogen bond with the adenine amine (Fig. 6*C* and *SI Appendix*, Fig. S23). Additional polar interactions coordinate the ribose and homocysteine groups, consistent with conserved cofactor recognition in class I MTs (67–71).

Comparison with structurally related MTs identified using the DALI server (72) indicates that Dol15G adopts a cofactor-bound, substrate-free conformation (*SI Appendix*, Fig. S24). In contrast to substrate-bound homologs (e.g., PDBID: 3GWZ and 8RDN), which undergo conformational rearrangements to enclose the substrate, Dol15G retains a more open active site architecture. This observation suggests that substrate binding may trigger local conformational changes during catalysis. To investigate substrate recognition, we docked dolastatin 15 into the SAH-bound Dol15G structure (Fig. 6*D*). The model reveals a hydrophobic pocket formed by Phe247, Met276, and Leu298 that accommodates the aromatic moiety of the substrate, while Thr155 and Arg244 are positioned to engage nearby carbonyl groups. These interactions provide a structural basis for both regioselective *O*-methylation and the strict stereochemical preference observed in biochemical assays. Together, these results identify Dol15G as the SAM-dependent O-MT responsible for the terminal tailoring step in dolastatin 15 biosynthesis and uncover a molecular basis for *O*-methylated tetramic acid formation.

## Discussion

The cyanobacterial genus *Lyngbya* has long been recognized as a polyphyletic group comprising morphologically similar yet phylogenetically distinct taxa (29). Subsequent molecular phylogenetic studies have led to reclassification of several *Lyngbya*-like cyanobacteria into new genera, including *Okeania*, *Moorena*, *Caldora,* and *Dapis* (27, 29, 73, 74). Although *Dapis* was established as a distinct genus in 2018 (29), its genomic features and biosynthetic potential have remained largely unexplored (30). Here, we report complete genomes of *Dapis* spp. and identify the *dol15* BGC, establishing *Dapis* as a previously unrecognized cyanobacterial lineage capable of producing pharmacologically relevant natural products. Consistent with this emerging chemical potential, mildly cytostatic kahalalide F analogs (dapalides A-C) were recently isolated from a marine *Dapis* strain collected from Guam (31), while iheyamides A-C with antitrypanosomal activity were reported from another marine *Dapis* strain (32).

Dol15D-M2 contains a noncanonical five-domain NRPS module in which an embedded KAL domain mediates 2-keto acid activation (Fig. 4). Comparative analyses suggest that this catalytic strategy extends beyond the dolastatin pathway and is distributed across diverse bacterial lineages (Fig. 5*A*). Phylogenetic analyses further indicate that these KAL domains form a distinct clade within the ANL superfamily, separate from canonical NRPS A domains and fatty acyl ligases (*SI Appendix*, Fig. S14), consistent with specialization in keto acid activation and expansion of NRPS substrate scope beyond amino acids. Beyond providing evolutionary insight, KAL-mediated activation followed by ketoreduction provides a genetically encoded route for incorporating hydroxy acid building blocks into peptide backbones (*SI Appendix*, Fig. S9*A*). Because amide-to-ester substitutions can alter the conformational, physicochemical, and pharmacological properties of peptide scaffolds (e.g., improved membrane permeability) (75), embedded KAL modules may provide a complementary biosynthetic platform for engineering depsipeptide analogs with tailored backbone architectures and expanded chemical diversity (76, 77).

Comparison of KAL-containing NRPS modules further reveals the architectural flexibility of NRPS assembly lines. Whereas previously characterized systems typically employ four-domain modules (C-KAL-KR-T) with specialized KAL domains, Dol15D-M2 and PyrG adopt a five-domain organization (C-A_x_-KAL-KR-T) in which keto acid activation is mediated by an embedded KAL domain (Figs. 4 and 5). This divergence demonstrates that distinct NRPS architectures can accomplish the same overall chemical transformation. Comparative structural analyses further raise the possibility that the A_x_ domain has undergone progressive reduction and loss of catalytic function while retaining a potential structural role in maintaining module organization (Figs. 4 and 5 and *SI Appendix*, Figs. S6 and S12). Although the evolutionary origins of these configurations remain unresolved, the coexistence of four- and five-domain modules highlights the remarkable evolutionary plasticity of NRPS systems and illustrates how domain recruitment, degeneration, and functional specialization can collectively expand substrate scope and biosynthetic diversity.

The classifiers achieved excellent performance in distinguishing KAL and canonical NRPS A domains (Fig. 5*C*), but several limitations should be considered when interpreting these results. First, the training dataset comprised 219 nonredundant KAL domains and a balanced reference set of 169 canonical A domains, and therefore may not fully capture the sequence diversity of this emerging enzyme subfamily. Second, many KAL annotations were derived from genome mining rather than direct biochemical characterization, introducing uncertainty into the reference labels. Third, the current framework relies exclusively on ESM-derived sequence embeddings and does not incorporate complementary information, such as three-dimensional structural features, substrate-recognition determinants, or neighboring biosynthetic context. Future work incorporating larger experimentally validated datasets together with multimodal sequence, structural, and biosynthetic information may further improve model robustness and facilitate functional annotation of bioinformatically predicted KAL domains.

Biochemical and structural characterization of Dol15G further completes the biosynthetic logic of dolastatin 15 by validating its proposed terminal tailoring step. Dol15G catalyzes *O*-methylation of a tetramic acid moiety (*SI Appendix*, Fig. S9*B*), a structural feature widely observed in marine cyanobacterial metabolites, including dolastatin analogs and other pyrrolinone-containing natural products such as jamaicamide, malyngamides, and mirabimides (60, 61). Despite the prevalence of this motif, its biosynthetic origin has remained poorly understood. Notably, the C-terminal pyrrolinone of iheyamide A has been shown to be critical for biological activity (32, 78), underscoring the functional importance of this modification. Our results provide a molecular basis for *O*-methylated pyrrolinone formation and a foundation for identifying and engineering related biosynthetic pathways.

Formation of the *N*-acylated pyrrolinone ring is proposed to be catalyzed by the TE domain of Dol15F through intramolecular cyclization involving a secondary amide nitrogen as the nucleophile (Fig. 3*C* and *SI Appendix*, Fig. S9*B*), representing an unusual variant of TE-mediated cyclization (79). Although a standalone C-like enzyme (JamQ) has been proposed to catalyze similar cyclization in jamaicamide biosynthesis (80), no such homolog is present in the *dol15* pathway. The frequent co-localization of Dol15F- and Dol15G-like enzymes across diverse BGCs suggests coordinated roles in methoxy pyrrolinone biosynthesis (*SI Appendix*, Fig. S25). Although in vitro reconstitution of Dol15F activity with a simplified SNAC substrate **5** (*SI Appendix*, Fig. S26) was unsuccessful, likely reflecting substrate complexity, structural modeling suggests extended peptide recognition may be required (*SI Appendix*, Fig. S27). Our proposed biosynthetic pathway involves TE-mediated tetramic acid formation, followed by tautomerization and O-methylation by Dol15G (Fig. 6 and *SI Appendix*, Fig. S9*B*). Although this sequence is consistent with the required chemical transformations, the detailed catalytic mechanism and structural basis of TE-mediated tetramic acid formation remain poorly understood. Future biochemical, structural, and computational studies will be required to resolve the reaction trajectory and catalytic basis of this transformation.

Together, this work defines the biosynthetic logic of clinically important dolastatin 15 and reveals a noncanonical NRPS strategy for 2-keto acid activation, while identifying *Dapis* as an underexplored reservoir of biosynthetic diversity. The substantial genomic and BGC diversity observed among closely related *Dapis* strains highlights the potential of this lineage for discovery of new dolastatin analogs and other structurally distinct natural products. More broadly, comparative genomic analyses of *Dapis*, together with recognition that embedded KAL domains function as alternative 2-keto acid-activating modules, provide a framework for understanding NRPS evolution and diversification and for engineering NRPS assembly lines to generate new depsipeptides and other noncanonical peptide scaffolds (76).

## Materials and Methods

### Sample Collection.

Cyanobacterial sample VPG23-37 was collected from Double Reef, Guam, at a depth of 20 to 30 ft on March 31, 2023. Sample VPG23-50 was collected from the reef outside Tumon Bay, Guam, at a depth of 30 to 35 ft on March 31, 2023. Sample VPG23-77 was collected from the southern side of Cocos Lagoon, Guam, at a depth of approximately 20 ft on April 4, 2023. Portions of each sample were stored frozen in RNAlater for genomic DNA isolation, while the remaining material was lyophilized for metabolite analysis.

Additional materials and methods can be found in *SI Appendix, Materials and Methods*.

## Supplementary Material

Appendix 01 (PDF)

Dataset S01 (XLSX)

Dataset S02 (XLSX)

Dataset S03 (XLSX)

## Data Availability

The genomes of *Dapis* sp. VPG23-37-b1, *Dapis* sp. VPG23-37-b2, *Dapis* sp. VPG23-50, and *Dapis* sp. VPG23-77 have been deposited at the GitHub repository under a folder named Genomes (58). The *dol15* BGC from *Dapis* sp. VPG23-77 has been deposited to GenBank under accession number PZ353175 (40). The Dol15G X-ray crystal structure has been deposited in the RCSB PDB under the accession number 10QU (64). Code and datasets for ESM2 embeddings and classification have been deposited at https://github.com/dingyous/A_domain_classification (58). All other data are included in the article and/or supporting information.
