# Supplementary material for: Biosynthetic origin of anticancer dolastatin 15 reveals a noncanonical NRPS architecture enabling 2-keto acid activation
Source: Proc Natl Acad Sci U S A. 2026 Sep 29;123(40):e2615454123. doi: 10.1073/pnas.2615454123 (PMC13643306; doi:10.1073/pnas.2615454123)
Supplement: Supplementary file 1 [file pnas.2615454123.sapp.pdf]

PNAS

Supporting Information for

Biosynthetic origin of anticancer dolastatin 15 reveals a noncanonical NRPS architecture enabling 2-keto acid activation

Manyun Chen[1], Jie Liu[2], Hsin-Ying Tsai[1], Cheng-Yu Li[3], Jeremiah D. Batucan[1], Campbell W. Eckhardt[1], Tam H.D. Pham[1], Emma K. Ellis[1], Ranjala Ratnayake[1], Valerie J. Paul[4], Steven D. Bruner[3,*], Mohamed S. Donia[2,*], Hendrik Luesch[1,5,*], Yousong Ding[1,*]

[1]Department of Medicinal Chemistry, Center for Natural Products, Drug Discovery, and Development, University of Florida, Gainesville, Florida 32610, United States

[2]Department of Molecular Biology, Princeton University, Princeton, New Jersey 08544, United States.

[3]Department of Chemistry, University of Florida, Gainesville, Florida 32611, United States.

[4]Smithsonian Marine Station, 701 Seaway Drive, Fort Pierce, Florida 34949, United States.

[5]Program in Cancer and Stem Cell Biology, Duke-NUS Medical School, Singapore 169857, Singapore.

Co-correspondence: ydingcop.ufl.edu; luesch@cop.ufl.edu; donia@princeton.edu; bruner@ufl.edu

This PDF file includes:

Supporting text
Figures S1 to S27
Tables S1 to S5
Legends for Dataset S1-3
SI References

Other supporting materials for this manuscript include the following:

Dataset S1-3

Supporting Information Text

Contents

Supporting Information Text 2
Experimental Details........ 3
Figures........ 10
Fig. S1. ^{1}H-NMR spectra (600 MHz) of dolastatin 15 in $CDCl_3$........ 10
Fig. S2. Phylogenetic tree of *Dapis* sp. based on 16S rRNA gene sequences. 11
Fig. S3. Phylogenomic tree of four *Dapis* strains and *Trichodesmium*. 12
Fig. S4. Gene cluster family analysis of BGCs from *Dapis* sp. and reference cyanobacterial genomes. 13
Fig. S5. Domain architecture of Dol15D-M2........ 14
Fig. S6. Sequence alignment analysis shows the conserved motifs in Dol15D-M2 A_x domains. . 15
Fig. S7. Structure alignment of StsA-KAL with AlphaFold 3 predicted Dol15D-KAL. 16
Fig. S8. Sequence alignment of Dol15D-M2 KAL and selected KAL domains. 17
Fig. S9. Proposed biosynthetic sequence for hydroxy acid incorporation and methoxy pyrrolinone formation........ 18
Fig. S10. Size distribution of identified C-A_x-KAL-KR-T and C-KAL-KR-T homologs. 19
Fig. S11. Domain organization of selected NRPSs containing Dol15D-M2 homologs (A) or C-KAL-KR-T modules (B)........ 20
Fig. S12. Predicted structure of CrpD-M2 by AlphaFold 3. 21
Fig. S13. Comparative phylogenetic analysis of Dol15D-M2 (A) and CrpD-M2 homologs (B). 22
Fig. S14. Phylogenic tree of KAL domains with other members in the ANL family........ 23
Fig. S15. Visualization of ESM2 embedding space distinguishes KAL and canonical NRPS adenylation domains........ 24
Fig. S16. Phylogenic tree of KAL domains identified from cyanobacteria. 25
Fig. S17. Proposed biosynthetic pathway of symplostatin 3. 26
Fig. S18. Representative cyanobacteria natural products containing C-terminal *N*-acylated pyrrolidine-2,4-dione........ 27
Fig. S19. SDS-PAGE of recombinant proteins........ 28
Fig. S20. ^{1}H NMR spectrum of crude synthetic 3 in DMSO-d_6 (600 MHz) at 25 °C........ 29
Fig. S21. ^{1}H NMR spectrum of synthetic 4 in $CDCl_3$ (600 MHz) at 25 °C. 30
Fig. S22. ^{13}C NMR spectrum of synthetic 4 in $CDCl_3$ (150 MHz) at 25 °C. 31
Fig. S23. Ligplot of SAH in Dol15G. 32
Fig. S24. Structural comparison of different O-methyltransferase homologs (A) Dol15G, (B) 3GWZ, (C) 3LST, (D) 8RDN, (E) 8TJJ, and (F) 1KYW. 33
Fig. S25. Bioinformatic analysis revealed frequent co-localization of genes encoding Dol15F-G. 34
Fig. S26. ^{1}H NMR spectrum of crude synthetic 5 in $CDCl_3$ (600 MHz) at 25 °C. 35
Fig. S27. Structure of Dol15F-TE binding with compound 2 predicted by AlphaFold 3. 36

Tables........ 37
Table S1. AntiSMASH BGC prediction of *Dapis* sp. VPG23-77........ 37
Table S2. Features of the gene product in the *dol15* BGC from *Dapis* sp. VPG23-77. 38
Table S3. Substrate prediction of the adenylation domains in the *dol15* BGC. 39
Table S4. Data collection and refinement statistics of Dol15G. 40
Table S5. Primers and synthesized genes used in this study........ 41

Dataset (separate file). (S1) Genomic sequencing and genome assembly statistics of four new *Dapis* strains. (S2). Genomic features of *Dapis* strains and cyanobacteria references used in phylogenomic analysis. (S3) Orthogroup analysis of five *Dapis* strains and *Trichodesminum erythraeum* IMS101. 42

Experimental Details

Sample collection. The cyanobacteria sample VPG23-37 was collected from Double Reef, Guam at a depth of 20-30 feet on March 31, 2023; VPG23-50 was collected from the reef outside Tumon Bay, Guam at a depth of 30-35 feet on March 31, 2023; and VPG23-77 was collected from the southern side of Cocos Lagoon at a depth of ~20 feet, Guam on April 4, 2023. The cyanobacteria samples were freeze-stored in RNAlater solutions for genomic DNA isolation or lyophilized for metabolite analysis.

Detection, isolation and structure validation of dolastatin 15. The lyophilized cyanobacteria samples were extracted by 1:1 EtOAc:MeOH for LC-HRMS analysis using a Thermo Scientific Q-Exactive Mass spectrometer equipped with Electron Spray Ionization and Kinetex 2.6 µm C18 100 Å, 100 × 4.6 mm column. The method used a linear gradient of 10%-95% CH_3CN/H_2O (both with 0.1% HCOOH) for 15 min, followed by an isocratic elution of 95% CH_3CN/H_2O (both with 0.1% HCOOH) for 5 min at 0.5 mL/min flow rate. Discovery method (dd-MS2) in positive mode was employed with scan range of 113 to 1695 m/z and collision energy (NCE) set to 20, 30, and 40 eV. To purify dolastatin 15 for structure validation, the freeze-dried (11.485 g) biomass of VPG23-37 was soaked in 1:1 EtOAc/CH_3OH to yield 201.2 mg of non-polar crude extract. It was sequentially solvent-partitioned using 1:1 EtOAc/H_2O (3×), followed by *n*-BuOH to yield EtOAc fraction (37.8 mg), semi-polar BuOH fraction (9.9 mg), and polar H_2O fraction (135 mg). The EtOAc partition was eluted through an SPE-C18 with 100% CH_3OH and evaporated to dryness. Further fractionation *via* RP-HPLC was done using a semipreparative (10 mm × 250 mm) Luna C18 column, 5 µm, and a linear gradient of 70%-100% CH_3CN/H_2O for 45 min, followed by 100% CH_3CN for 10 min, at 2.5 mL/min flow rate to yield dolastatin 15 (t_R = 34.5 min, 2.4 mg). This assignment was corroborated by ^{1}H NMR spectrum having identical chemical shifts with the literature values (1, 2). Additional material was purified from VPG23-77 from the 1:1 EtOAc/H_2O extract through the same solvent partitioning to yield 27.2 mg of the EtOAc fraction, which was then partitioned into 14 silica fractions using a DCM:IPA gradient, in which dolastatin 15 was detected in the 8% IPA:DCM fraction (2.6 mg). This fraction was subjected to C18 clean up, then further purified through RP-HPLC using an analytical Luna C18 column (4.6 mm × 250 mm) with a linear gradient of 10%-100% MeCN in 20 min, followed by 100% MeCN for 15 min at 1 mL/min. This yielded the pure compound in <0.1 mg (t_R = 17 min), thus detected from LC-HRMS analysis.

Genome sequencing, assembly and annotation. The metagenomic DNA of *Dapis* sp. VPG23-37, VPG23-50 and VPG23-77 were extracted using the QIAamp DNA Micro Kit (Qiagen) and further purified with the DNeasy PowerClean Pro Cleanup kit (Qiagen). The metagenome sequencing was performed first with Illumina NovaSeq, SP 2 x 250 bp platform and then with the PacBio SEQUEL IIe sequencing platform with a SMRT cell at the UF Interdisciplinary Center for Biotechnology Research. PacBio HiFi reads of each sample were assembled using metaFlye (3) and hiCanu (4). The assemblies from two pipelines were binned independently using the HiFi-MAG-Pipeline (5). Quality of *Dapis* MAGs from two individual assemblies were evaluated using checkM2 (6) and the one with higher contiguity were retained for further analysis. Biosynthetic gene cluster (BGCs) were predicted using antiSMASH 8.0 (7). The dolastatin 15 (*dol15*) BGC was identified based on predicted synthetic modules and further corrected by quality filtered Illumina reads using Pilon (8). Corrected *Dapis* genomes were uploaded to the Integrated Microbial Genome platform (IMG, http://img.jgi.doe.gov) for whole genome annotation and analysis.

Phylogenetic and phylogenomic analyses. Each *Dapis* strain genome harbors two divergent 16S rRNA gene copies. Two copies of 16S rRNA gene were BLAST to NCBI ribosome database and the one with higher identity to a known reference was selected as representative 16S rRNA and used for phylogenetic analysis. Sequences for 16S rRNA phylogenetic analysis were aligned using MAFFT (9) and phylogenetic tree was constructed using RAxML in Geneious with 500 bootstrap replicates (10). Genomes and MAG references used for phylogenomic analyses were downloaded from NCBI GenBank and annotated using Prokka (11). Single copy genes from new *Dapis* strains and these cyanobacteria genomes and MAGs were identified using OrthoFinder (12) and concatenated for alignment using MAFFT (9). Best-fit evolutionary model for the concatenated alignment was selected by ModelTest-NG (13) and phylogenetic tree was built using RAxML-NG

with 1000 bootstrap replicates (14). Whole genome-based taxonomy classification of these genomes and MAGs was determined using GTDB-Tk (15). Genome features were analyzed for BGC contents via antiSMASH, insert element (transposases) via ISEScan (16) and CRISPR-Cas via CRISPRtyper (17). Phylogenomic tree and corresponding annotated genomic features were plotted using iTOL (18).

Comparative genomic analysis. Genome comparison was conducted among four new *Dapis* strains, *Dapis* sp. VPG-23-80 MAG1 and *Trichodesmium erythraeum* IMS10, with *Dapis* sp. VPG23-77 selected as reference. Complete genome and annotation of *T. erythraeum* IMS101 was retrieved from GenBank (GCA_000014265) and *Dapis* sp. VPG-23-80 MAG1 was annotated using Prokka (11). The COG assignment for these two genomes was performed using hmmsearch to COG2014 database in the same manner as JGI annotation pipeline for four new *Dapis* genomes (19). ANI among genomes is calculated using orthoANI (20). Genome alignment was performed using MuMmer (21) and plotted using Circos with regions >=80% identity (22). Orthoglog groups (OGs) analysis among these six genomes listed above was performed using OrthoFinder (12). OG categories were classified based on their prevalence in these genomes. Genes in each OG were counted for their assigned COG distribution.

BGC network analysis and identification of the *dol15* BGC. AntiSMASH 8.0 (7) was used to predict the BGCs from the genomes of dolastatin 15 producing *Dapis* sp. reported in this study and other MAGs of *Dapis* sp. classified in **Fig. S3**. To evaluate the novelty and conservation of *Dapis* BGCs, we clustered them with BGCs from 385 complete cyanobacteria genomes from the NCBI database. A total of 3305 BGCs including 217 *Dapis* BGCs were analyzed by BiGSCAPE 2.0 (23) with a cutoff threshold at 0.3. The singletons were excluded from the output and the clusters containing at least one *Dapis* BGC were visualized in Cytoscape (24). The BGCs from *Dapis* sp. VPG23-77 were first analyzed by AntiSMASH, and the putative *dol15* BGC was mined against the NCBI nr database. The homologous BGCs were aligned by clinker (25). The substrate specificity of the adenylation domains encoded in the dol15 BGC were predicted by PARAS (26).

Evolution analysis of keto acid–activating NRPS modules. To understand the diversity and distribution of the Dol15D-M2, it was mined against the NCBI RefSeq database and antiSMASH 5.0 database, and a total of 125 homologs with C-A_x-KAL-KR-T domains were retrieved (34 from cyanobacteria). Similarly, 337 CrpD-M2 homologs were identified. Architecture prediction was confirmed through antiSMASH v 8.0 for all sequences. All Dol15D-M2 homologs and CrpD-M2 homologs were separately aligned with MAFFT (9) using a L-INS-I model. Subsequent trimming using TrimAl v 1.4 (27) revealed a sharp reduction in alignment length at low gap thresholds (≤0.2), consistent with the removal of highly gapped regions. Across a broad intermediate range of thresholds (0.2–0.7), alignment length was relatively stable, indicating convergence on a shared set of well-represented columns. A threshold of 0.5 was selected for downstream analyses as a representative value within this stable region. Model selection was first performed using IQ-TREE v3 (28, 29), which identified WAG+F+I+G4 as the best-fitting amino acid substitution model under the Bayesian Information Criterion (BIC). These alignments were used for the evolutionary analysis software PhyML_Multi (30) to determine evolutionary recombination points using two or three trees under an HMM that together best fit the alignments. Maximum likelihood phylogenetic inference in PhyML_Multi was performed using the substitution model selected above. Tree topology and branch lengths were optimized using BioNJ (31). A proportion of invariable sites was estimated to account for site-specific evolutionary constraint, and among-site rate heterogeneity was modeled using a discrete gamma distribution with four rate categories, while empirical amino acid frequencies were computed from the observed alignment. To analyze the two tree outputs, the log-likelihood of tree 2 was subtracted from tree 1. To analyze the three-tree outputs, normalized probabilities were derived from their log-likelihood scores. For each tree *i*, the difference between its log-likelihood ($\ln L_i$) and the maximum log-likelihood across all trees ($\ln L_{max}$) was calculated to improve numerical stability. These values were then exponentiated to obtain relative likelihoods. Finally, probabilities were computed by dividing each relative likelihood by the sum across all trees, yielding normalized weights that sum to 1. To reduce local fluctuations in the probability profiles for both two tree and three tree analyses, values were smoothed using a centered sliding window. For

each site, the reported value represents the mean across a window spanning 10 sites upstream and 10 sites downstream (window size = 21 sites) with truncation at sequence boundaries.

Structure prediction and phylogenic analysis of Dol15D-M2 KAL. The domain architecture of Dol15D-M2 was first predicted by NCBI conserved domain analysis and Pfam analysis. We then predicted the structure of Dol15D-M2 through AlphaFold 3 (32) and determined the domain boundaries. A structure model of the KIV adenylate binding with Dol15D-KAL was also predicted by AlphaFold 3. The cyanobacteria NRPS modules with C–A_x–KAL–KR–T or C–KAL–KR–T domain aligned in MAFFT and their KAL domains were retrieved for phylogenic analysis using the maximum likelihood algorithm in MEGA with 1000 bootstrap replicates. Similar approach was applied to construct another phylogenic tree for Dol15-M2 KAL with other members in the adenylate ligase superfamily, including fatty acyl-CoA ligases, fatty acyl-AMP ligases, and the adenylation domains collected from MiBiG (33) database with known substrate specificity for 2-keto acids, hydroxyl acids or L-valine.

Machine learning approach to distinguish KAL and canonical A domains. Protein sequences corresponding to KAL (annotated CAL and A_k) domains were collected from antiSMASH 5.0 database and NCBI (34). Domain annotations were parsed from GenBank files using NRPS/PKS feature qualifiers. For KAL domains, sequences were extracted from NRPS modules that contained a KR domain within the same coding sequence. Candidate regions were identified by scanning for NRPS architectures in which a KR domain occurred between upstream condensation (C) and downstream carrier protein (T/PCP) domains. For A_k domains, sequences annotated as AMP-binding domains were included, while CAL domains were identified based on antiSMASH "CAL_domain" annotations. Canonical A domain sequences were collected by parsing through genomes of hits found in the CAL/A_K pipeline. Sequences were then aligned using MAFFT (9) using the L-INS-i algorithm depending on their code (A, A_k, or CAL). Alignments were manually trimmed to include only the AMP-binding domain. To minimize redundancy and reduce potential overfitting. The dataset comprised 135 CAL, 169 canonical A, and 84 A_k domains using the cutoff of 85% identity. Sequence representations were generated using the pretrained protein language model ESM-2 (35). The ESM-2 650M parameter model (33 layers) was used to encode each protein sequence into a high-dimensional embedding. For each sequence, the per-residue representations from the final layer were extracted, and a mean pooling operation across all residues was applied to obtain a fixed-length vector representation (1280 dimensions).

A supervised binary classification model was developed using the ESM-2 embeddings to distinguish canonical A domains from KAL domains. For binary classification, CAL and A_k domains were combined into a single class, referred to as KAL, while canonical A domains constituted the second class. Four machine learning algorithms were evaluated, including logistic regression, support vector machines (SVMs), random forests, and XGBoost. The dataset was divided into training and testing sets using an 80:20 stratified split. Model selection and hyperparameter evaluation were performed using stratified five-fold cross-validation on the training set, ensuring that the proportions of A and KAL domains were maintained in each fold.

Model performance was evaluated using accuracy, area under the receiver operating characteristic curve (AUC), precision, recall, and F1 score. KAL was defined as the positive class and canonical A as the negative class. True positives (TP) represent KAL domains correctly classified as KAL, true negatives (TN) represent canonical A domains correctly classified as A, false positives (FP) represent canonical A domains incorrectly classified as KAL, and false negatives (FN) represent KAL domains incorrectly classified as A.. These metrics are defined as follows:

$$Accuracy = \frac{TP + TN}{TP + TN + FP + FN}$$

$$Precision = \frac{TP}{TP + FP}$$

$$Recall = \frac{TP}{TP + FN}$$

$$F1\text{-}Score = 2 \times \frac{\text{Precision} \times \text{Recall}}{\text{Precision} + \text{Recall}}$$

The AUC measures the area under the receiver operating characteristic (ROC) curve, which is generated by plotting the true positive rate (recall) against the false positive rate across all possible classification thresholds.

Molecular cloning, expression and purification of recombinant proteins. To construct the expression vectors for Dol15D-A_x and Dol15D-KAL, codon optimized genes were synthesized by Twist Biosciences and inserted into the *NdeI* and *XhoI* restriction sites of pET28b through T4 ligation (Thermo). The sequence for recombinant Dol15D-KAL also carried a 67 aa N-ter flanking region between A_x and KAL. The mutant for Dol15D-KAL-V219D was created by the PCR amplification of the left fragment (N-terminus to the mutation site) and the right fragment (the mutation site to the C-terminus) and ligation of the fragments carrying V219D mutation into the *NdeI* and *XhoI* restriction sites of pET28b through Gibson assembly (NEB). To construct the expression vectors for Dol15G and Dol15F-TE, corresponding genes were cloned from the genomic DNA of *Dapis* sp. VPG23-77, and digested genes were inserted into the *NdeI* and *XhoI* restriction sites of pET28b. All the constructs were confirmed by Sanger sequencing at GENEWIZ. Constructs for adenylation domains were transformed into BL21-CodonPlus (DE3)-RIPL, and the constructs for Dol15G and Dol15F-TE were transformed into BL21-GOLD(DE3) for protein expression. Protein expression was carried out in 1 L Luria-Bertani broth supplemented with 50 µg/mL kanamycin (37 °C, 225 rpm). When the cell culture OD_{600} reached 0.6, IPTG (final concentration 0.1 mM) was added to the culture to induce protein expression (16 °C, 180 rpm, 20 h). The cells were harvested by centrifugation (3,500 xg, 15 min), and collected cell pellets were resuspended in the lysis buffer (25 mM HEPES, pH 7.5, 100 mM NaCl, 0.5 mg/mL lysozyme, 1 mM phenylmethylsulfonyl fluoride, 1 mM β-mercaptoethanol and 10 mM imidazole) and lysed by sonication on ice (5 s pulse and 10 s rest, 5 min in total). Following centrifugation (15,000 xg, 4 °C, 30 min), the cell lysates were incubated with the HisPur™ Ni-NTA Resin (GE Healthcare) at 4 °C for 1 h and then washed with wash buffer (25 mM HEPES, pH 7.5, 250 mM NaCl, 1 mM β-mercaptoethanol and 30 mM imidazole) for three times and then the N-His6-taggaed proteins were eluted with the elution buffer (25 mM HEPES, pH 7.5, 250 mM NaCl, 1 mM β-mercaptoethanol and 300 mM imidazole). Recombinant proteins were collected, concentrated and buffer-exchanged into storage buffer (25 mM HEPES, pH 7.5, 10% glycerol). The purity of the recombinant protein was analyzed on SDS-PAGE, and the concentration was determined by NanoDrop.

Substrate specificity assay of Dol15D-M2 KAL. To monitor the adenylation activity of Dol15-M2 KAL and its V219D variant, the *in-vitro* biochemical reactions were set in 100 µL reaction buffer containing 50 mM HEPES pH at 7.5, 10 mM $MgCl_2$, 2 µM enzyme and 2 mM substrate. Substrates tested in this study include KIV, HIVA and L-Val. The reactions were initiated by the addition of 1 mM ATP (final concentration) and incubated at 25 °C for 1 h. Pyrophosphate generated in the reactions were quantified by a fluorescence assay using the PiPer™ Pyrophosphate Assay Kit (Invitrogen) following manufacturer's protocol. Briefly, 5 µL adenylation reaction mixtures were diluted to 50 µL and mixed with 50 µL freshly prepared PiPer™ working solution containing 100 µM Amplex® Red reagent, 0.02 U/mL inorganic pyrophosphatase, 4 U/mL maltose phosphorylase, 0.4 mM maltose, 2 U/mL glucose oxidase and 0.4 U/mL HRP. The mixtures (100 µL) were incubated in a 96-well plate at 37 °C for 15 min, protected from light, and immediately subjected to fluorescent detection with an excitation at 540 nm and emission detection at 590 nm. Each reaction was performed with at least four replicates. The background signal was determined by a blank control group containing heat deactivated adenylation enzyme and was subtracted in the calculation.

Chemical synthesis of the substrates used in this study.

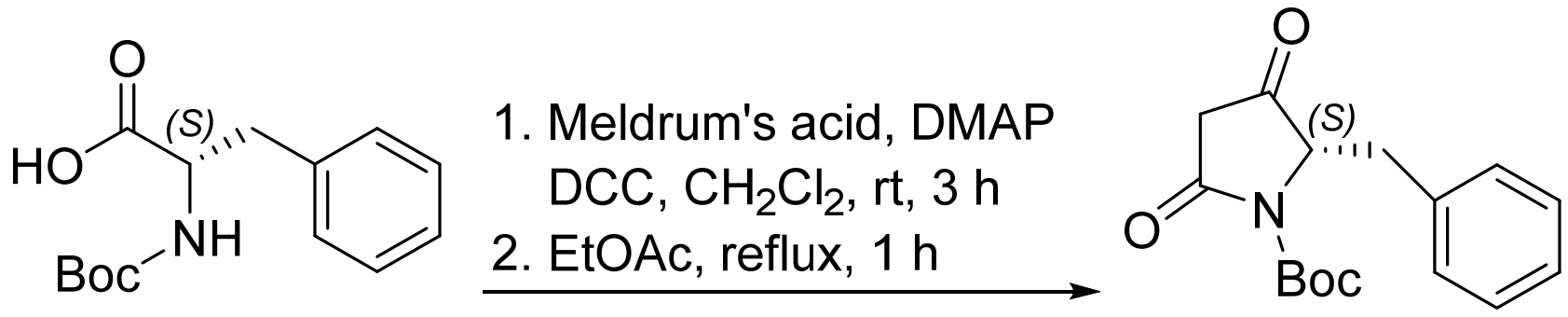

6 3

Compound **3** (Fig. S20) was synthesized based on a published protocol (36). To a stirred solution of *N*-Boc-L-phenylalanine (**6**) (506.5 mg, 1.91 mmol, 1.0 equiv) and Meldrum's acid (302.7 mg, 2.10 mmol, 1.1 equiv) in anhydrous CH_2Cl_2 (10 mL) at 0 °C were added 4-dimethylaminopyridine (DMAP) (349.9 mg, 2.86 mmol, 1.5 equiv), and *N*,*N'*-dicyclohexylcarbodiimide (DCC) (472.7 mg, 2.29 mmol, 1.2 equiv). After being stirred at 0 °C to room temperature for 3 h, the reaction mixture was filtered through a Celite pad and evaporated under pressure. The mixture was then resuspended in cold EtOAc (20 mL) and successively washed with cold 5% aqueous $KHSO_4$ (3 x 5 mL) and brine (1 x 5 mL), and the combined organic layer was then concentrated in vacuo for 30 min. The reaction mixture was then resuspended in anhydrous EtOAc and refluxed under heating for 1 h, cooled down to room temperature, and evaporated to give crude **3** as a light yellow solid (36, 37), which was washed with Et_2O to remove oily impurities and used in the next steps without further purification.

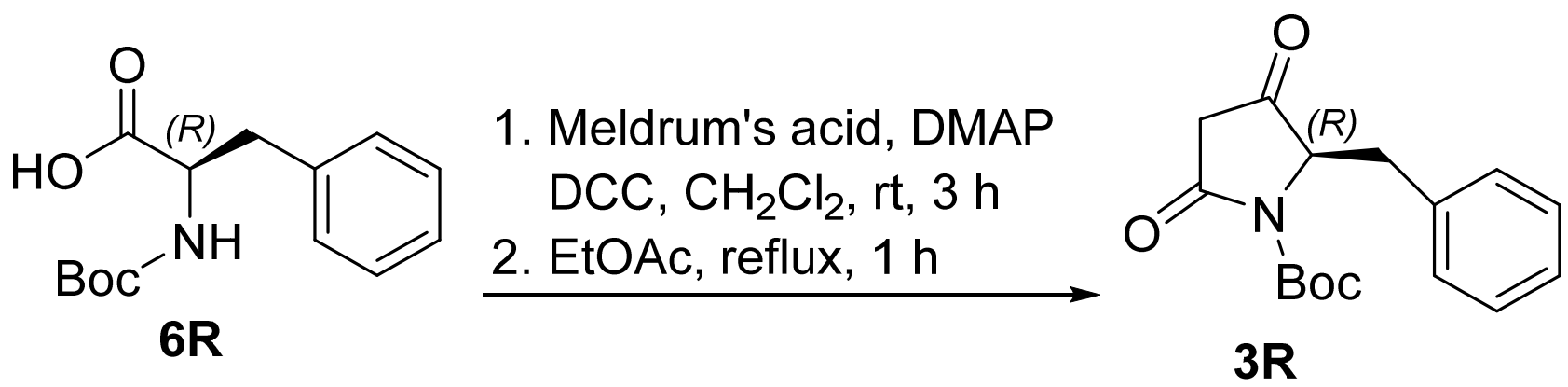

Compound **3R** was synthesized using the same protocol as mentioned previously using *N*-Boc-D-phenylalanine (**6R**).

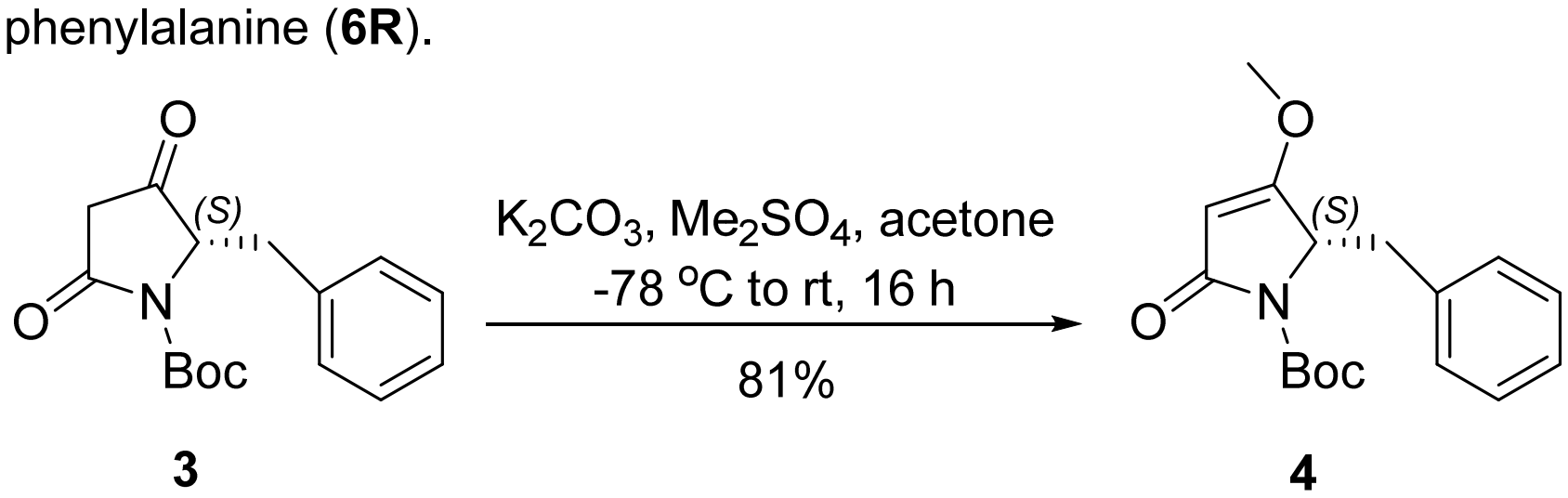

Compound **4** (Fig. S21-22) was synthesized based on a published protocol (37). To a mixture of the crude **3** (52 mg containing some impurity) and K_2CO_3 (37.3 mg, 0.27 mmol, 1.5 equiv) in acetone (1.5 ml) at −78 °C was added Me_2SO_4 (22.2 µl, 0.23 mmol, 1.3 equiv). After being stirred at −78 °C to room temperature for 16 h, the reaction mixture was filtered through a Celite pad with acetone and evaporated under pressure. The residue was purified by normal phase preparative TLC (1:1 EtOAc-hexanes) to afford **4** as a colorless amorphous solid (44.4 mg, 81%, Fig. S15-16): $[\alpha]^{21}_{D}$ -44.6 (*c* 1.0, $CHCl_3$); ^1H NMR (600 MHz, $CDCl_3$) *δ* 7.23–7.15 (m, 3H), 7.00–6.97 (m, 2H), 4.80 (s, 1H), 4.65 (dd, *J* = 4.8, 2.8 Hz, 1H), 3.76 (s, 3H), 3.43 (dd, *J* = -14.0, 5.0 Hz, 1H), 3.10 (dd, *J* = -14.0, 2.8 Hz, 1H), 1.59 (s, 9H); ^{13}C NMR (150 MHz, $CDCl_3$) *δ* 176.2, 168.8, 149.6, 134.2, 129.6, 128.3, 127.1, 95.3, 82.7, 60.2, 58.3, 35.4, 28.3.; HRMS (ESI) *m*/*z* calcd for $C_{17}H_{21}NO_4Na$ $[M + Na]^+$ 326.1368, found 326.1357.

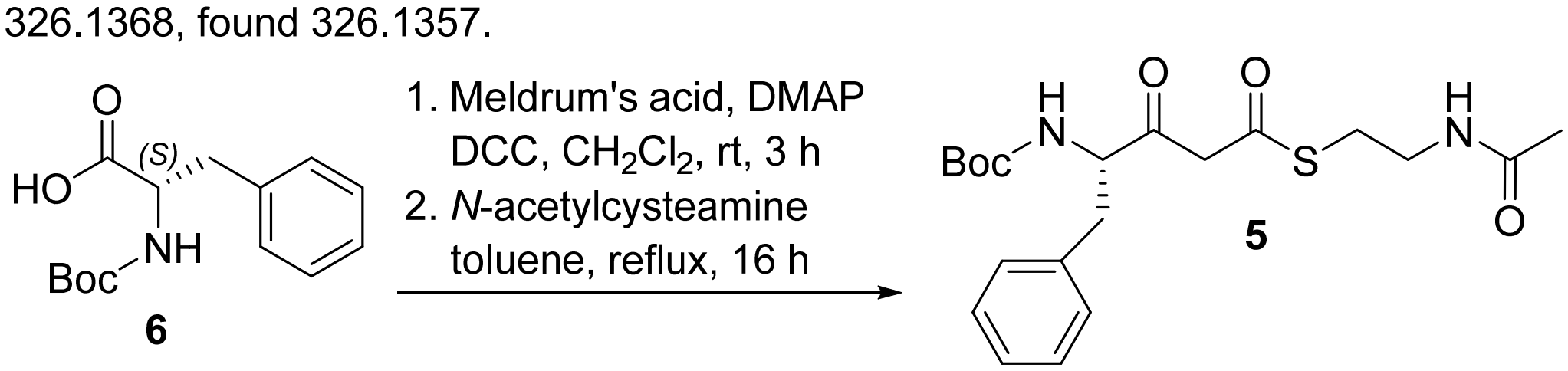

To a stirred solution of *N*-Boc-L-phenylalanine (**6**) (1.0 equiv) and Meldrum's acid (1.1 equiv) in anhydrous CH_2Cl_2 (5 mL) at 0 °C were added DMAP (1.5 equiv), and DCC (1.2 equiv). After being stirred at 0 °C to room temperature for 3 h, the reaction mixture was filtered through a Celite pad and evaporated under pressure. The mixture was then resuspended in cold EtOAc (20 mL) and successively washed with cold 5% aqueous $KHSO_4$ (3 x 5 mL) and brine (1 x 5 mL), and the combined organic layer was concentrated in vacuo for 30 min. The reaction mixture was then resuspended in anhydrous toluene and added 1.1 equiv of *N*-acetylcysteamine before refluxed under heating. After 16 h, the reaction mixture was diluted with EtOAc, washed excessively with brine, dried over $MgSO_4$, filtered, and concentrated in vacuo. The residue was purified by normal

phase preparative TLC (1:1 EtOAc-hexanes) to afford crude **5** (Fig. S26) as a colorless amorphous solid.

***In vitro* biochemical characterization of Dol15G.** The Dol15G full reaction (100 μL) was performed in 50 mM HEPES buffer at pH 7.5 containing 10 mM $MgCl_2$, 0.2 mM compound **3**, 2 mM SAM, and 2 μM Dol15G. The reactions were tested under different conditions including controls (1) using heat deactivated Dol15G; (2) in the absence of SAM; (3) without $MgCl_2$ and with the addition of 1 mM EDTA; (4) using compound **3R** as substrate. All reactions were incubated at 25 °C for 1 h and quench by adding equal volume of ice-cold acetonitrile. The reaction mixtures were then centrifuged (20,000 xg, 4 °C, 15 min) and analyzed by HPLC with an Agilent Poroshell 120 EC-C18 column (2.7 μm, 4.6 x 50 mm) using a Shimadzu Prominence UHPLC system with PDA detection. Solvent A was water and solvent B was acetonitrile. The column was equilibrated with 5% solvent B for 2 min and then eluted with a linear gradient of 10-50% in 8 min, followed by the other linear gradient of 50-95% solvent B in 5 min. The column was further eluted with 95% solvent B for 3 min and then re-equilibrated with 5% solvent B for 1 min. The flow rate was 0.5 mL/min. The reactions were monitored for the absorbance at 220 nm.

Crystallization of Dol15G. The expression plasmid pET28a-dol15G was transformed into BL21 (DE3) *E. coli*, and a 20 mL culture in LB medium, supplemented with 50 μg/mL kanamycin, was grown overnight at 37 °C. 10 mL of the liquid culture was added to 1 L of LB/kanamycin medium and incubated at 37 °C to an OD_{600}= 0.6–0.8, cooled to 16 °C, 0.1 mM IPTG was added for induction of protein expression, and the cells continued to grow overnight (~16 hours). Cells were harvested by centrifugation at 3500 rpm at 4 °C for 15 minutes, then resuspended in lysis buffer (20 mM Tris-HCl, pH 7.5, and 100 mM NaCl). Pelleted cells were lysed by sonication with 5-second pulse time at 25-second intervals for a total time of 2 minutes. The cell lysate was centrifuged at 13500 rpm / 4 °C for 30 minutes, and the supernatant was incubated with Ni-NTA resin for 1 hour. Bound recombinant protein was washed with lysis buffer containing 20 mM imidazole and eluted with lysis buffer containing 250 mM and 500 mM imidazole. The elution fractions were collected, 5U of thrombin (Sigma) was added to remove the hexa-histidine affinity tag, and the mixture was dialyzed against the lysis buffer overnight. Tag-free Dol15G protein was again applied to a Ni-NTA column, and the flow-through fraction was collected and further purified by size-exclusion chromatography using the HiLoad Superdex 200 pg 16/600 column in ÄKTA pure 25 chromatography system. Purified Dol15G was concentrated to 16 mg/mL and incubated with 5 mM *S*-adenosyl-L-homocysteine (SAH) for 30 mins at 4 °C before crystallization. Protein crystals were observed within a month in a reservoir solution containing 0.1 M HEPES-NaOH pH 7.5, 200 mM NaCl and 20% (w/v) polyethylene glycol 3000. Crystals were harvested, briefly soaked with 25% (v/v) glycerol as cryoprotectant and flash-frozen in liquid nitrogen. Diffraction data were collected on beamline 17-ID-1 (AMX) at Brookhaven National Laboratory NSLS with the wavelength 0.9197 Å and detector distance of 325 mm (38). Data were collected, integrated, merged, and scaled using the XDS package (39). An AlphaFold3 structure model (32) is used for molecular replacement and the structure was built using BUCCANEER (40) / COOT (41) and refined using Phenix Refine (42) and REFMAC (43).

***In vitro* biochemical characterization of Dol15F-TE.** The Dol15F-TE reactions (100 μL, 50 mM HEPES buffer at pH ranging from 6.5 to 8.0 or Tris-Cl buffer at pH ranging from 7.0 to 9.0) contained 0.5 mM SNAC substrate compound **5** and 2 μL Dol15F-TE. The reactions were incubated for 0.5 h, 1 h or 2 h at room temperature and were then quenched by adding 100 μL of ice-cold acetonitrile. After centrifugation (20,000 × g, 15 min, 4 °C), clear supernatants (10 μL) were immediately subjected for LC-HRMS analysis. LC-HRMS experiments were conducted on a Dionex Ultimate RSLC 3000 UHPLC system fitted with an Agilent Poroshell 120 EC-C18 column (2.7 μm, 4.6 x 50 mm) and a Thermo Scientific Q Exactive Focus mass spectrometer equipped with the H-ESI II probe on an Ion Max API Source. Solvent A was water with 0.1% formic acid (FA) and solvent B was acetonitrile with 0.1% FA. The column was equilibrated with 10% solvent B for 2 min and then eluted with a linear gradient of 10- 90% solvent B in 10 min, flushing with 90% solvent B for 2 min, followed with a linear gradient of 90-10% solvent B in 1 min and then re-equilibrated with 10% solvent B for 3 min. The flow rate was 0.5 mL/min. MS1 was acquired under a Full-Scan mode with

a mass range of 100−750, a resolution at 70,000 and AGC target at 1 × 10^6. Data were collected in both positive and negative ion modes.

Figures

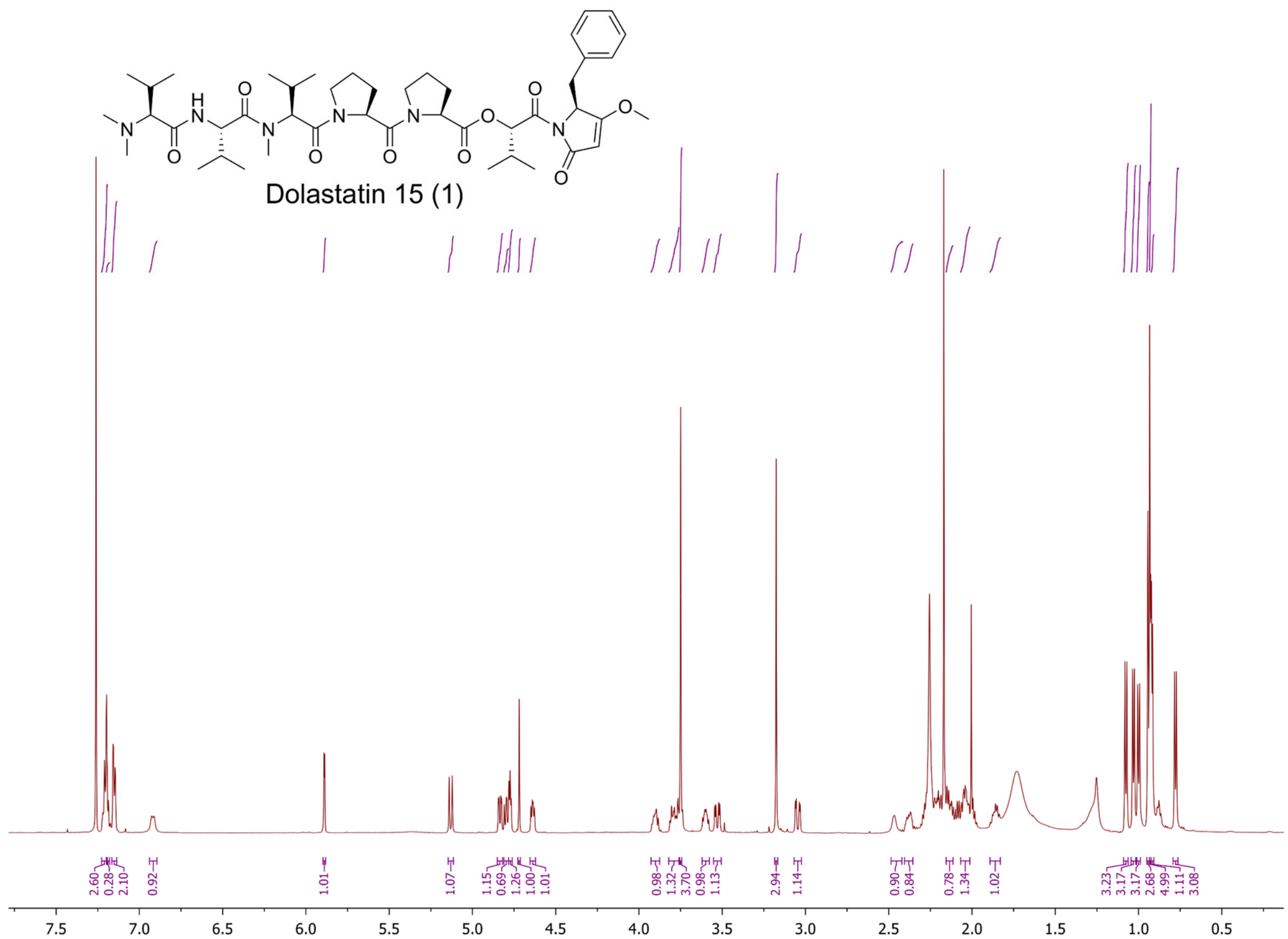

Fig. S1. ^{1}H-NMR spectra (600 MHz) of dolastatin 15 in $CDCl_3$.

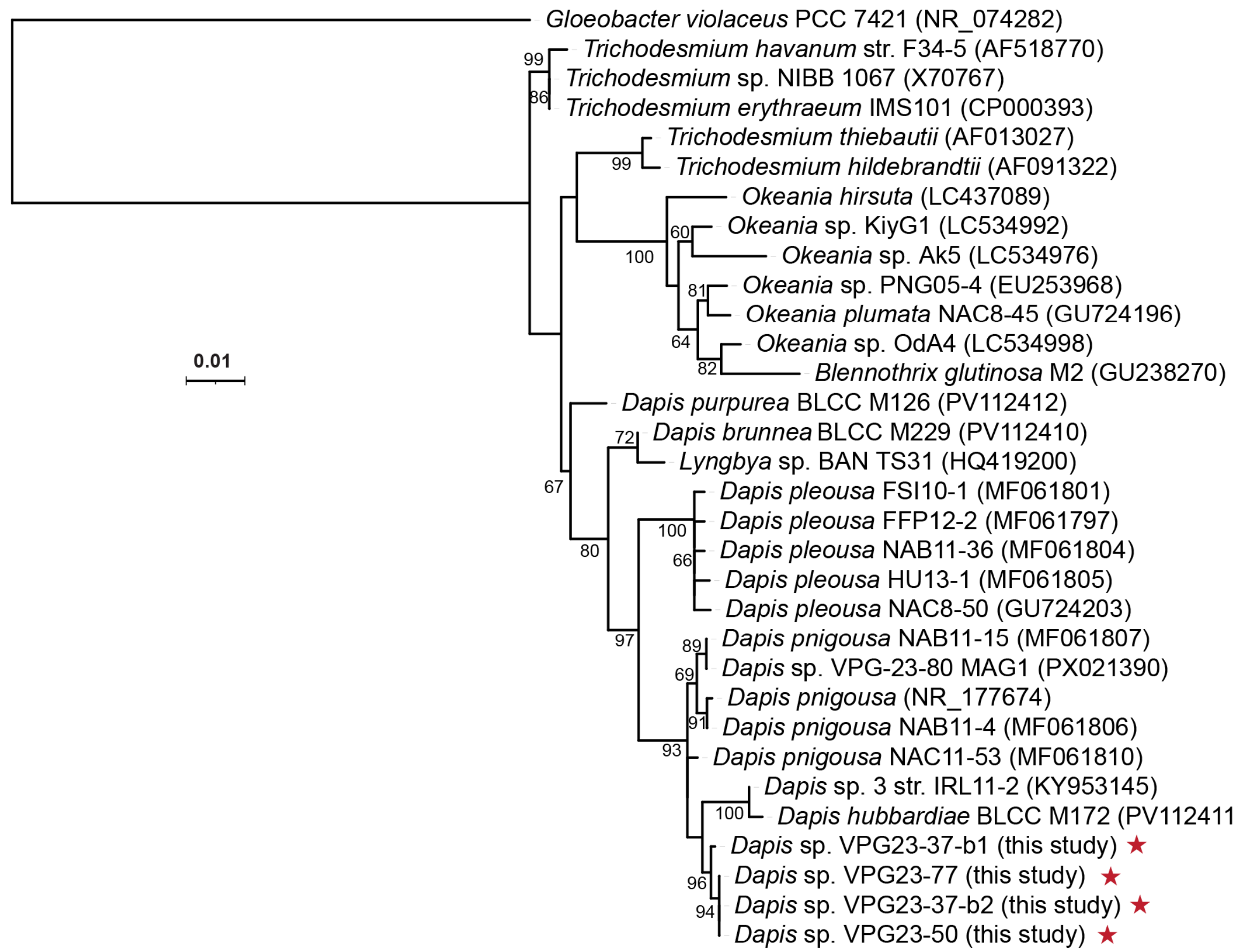

Fig. S2. Phylogenetic tree of *Dapis* sp. based on 16S rRNA gene sequences in comparison with closely related cyanobacterial references. Sequence alignment was performed using MAFFT and the phylogenetic tree was constructed using RAxML with 1000 bootstrap replicates. Bootstrap supports >=60 are shown on the tree. Four *Dapis* strains from this study are highlighted with red stars.

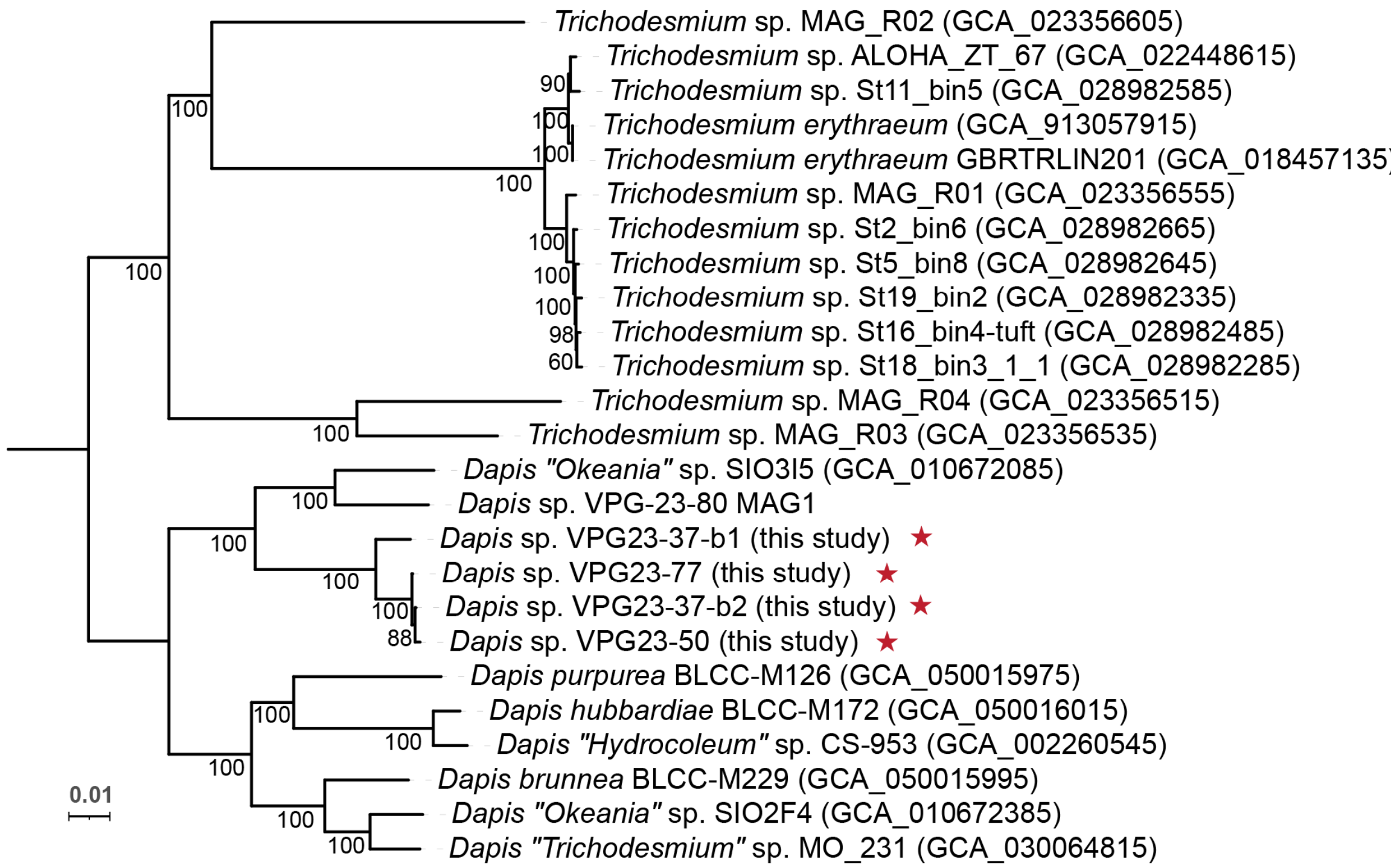

Fig. S3. Phylogenomic tree of four *Dapis* strains of this work along with 21 *Dapis* and *Trichodesmium* genomes and metagenome assembled genomes (MAGs) which are classified as "g_Trichodesmium" based on GTDBtk taxonomy classification. Phylogenetic tree was constructed using 271 concatenated single-copy genes identified from these genomes using OrthoFinder with 1000 bootstrap replicates. *Dapis* strains from this study are marked with red stars.

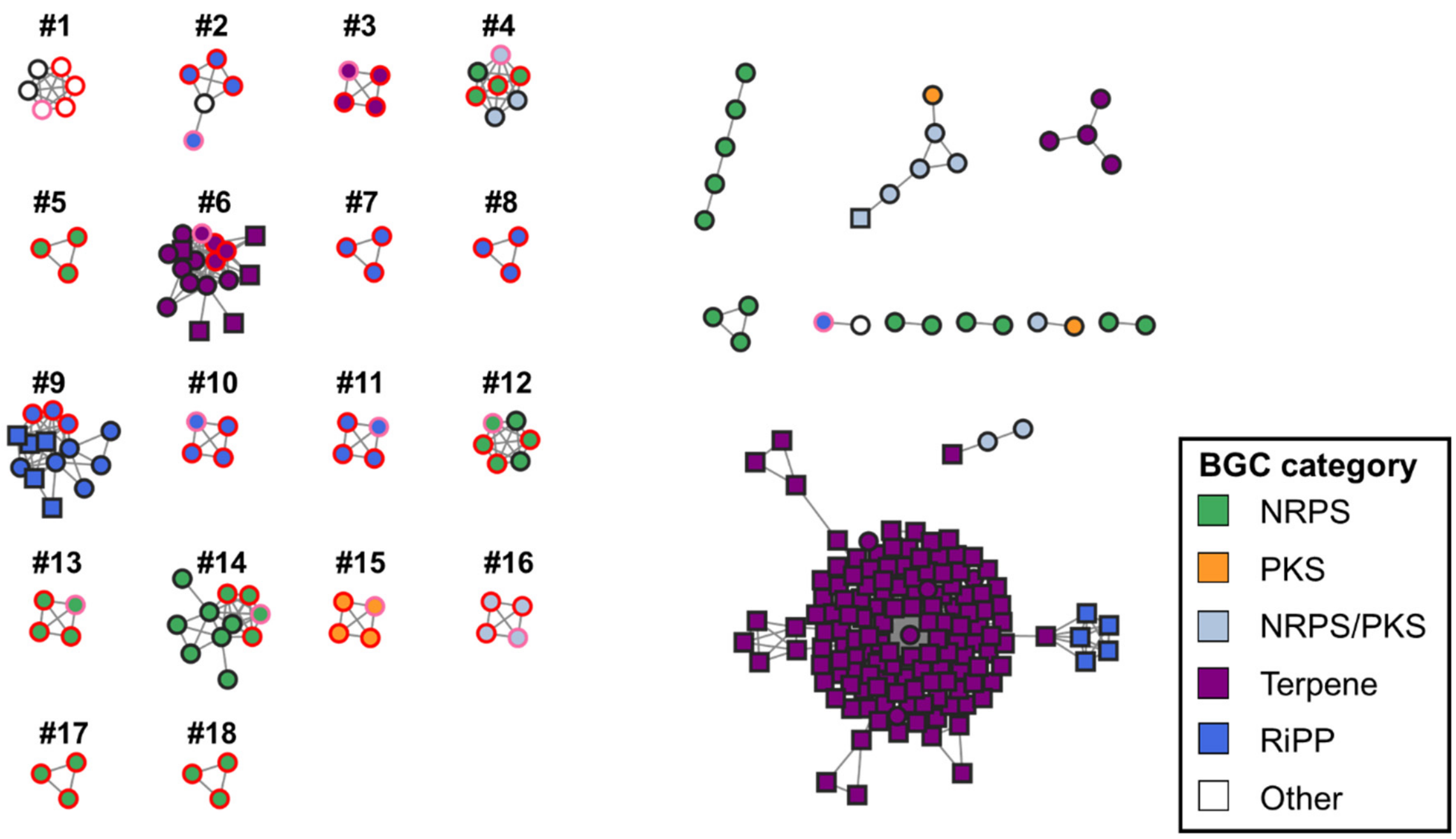

Fig. S4. Gene cluster family analysis of BGCs from *Dapis* sp. and 385 reference cyanobacterial genomes. A total of 3305 cyanobacterial BGCs including 217 from *Dapis* were analyzed by BiG-SCAPE with a cutoff threshold at 0.3 and the clusters containing *Dapis* BGCs were visualized. from *Dapis* sp. VPG23-77, VPG23-50 and VPG23-37-b2 are marked with red circles; BGCs from *Dapis* sp. VPG23-37-b1 were marked with pink circles; other *Dapis* BGCs are marked with black circles; and non-*Dapis* BGCs are shown in rectangles.

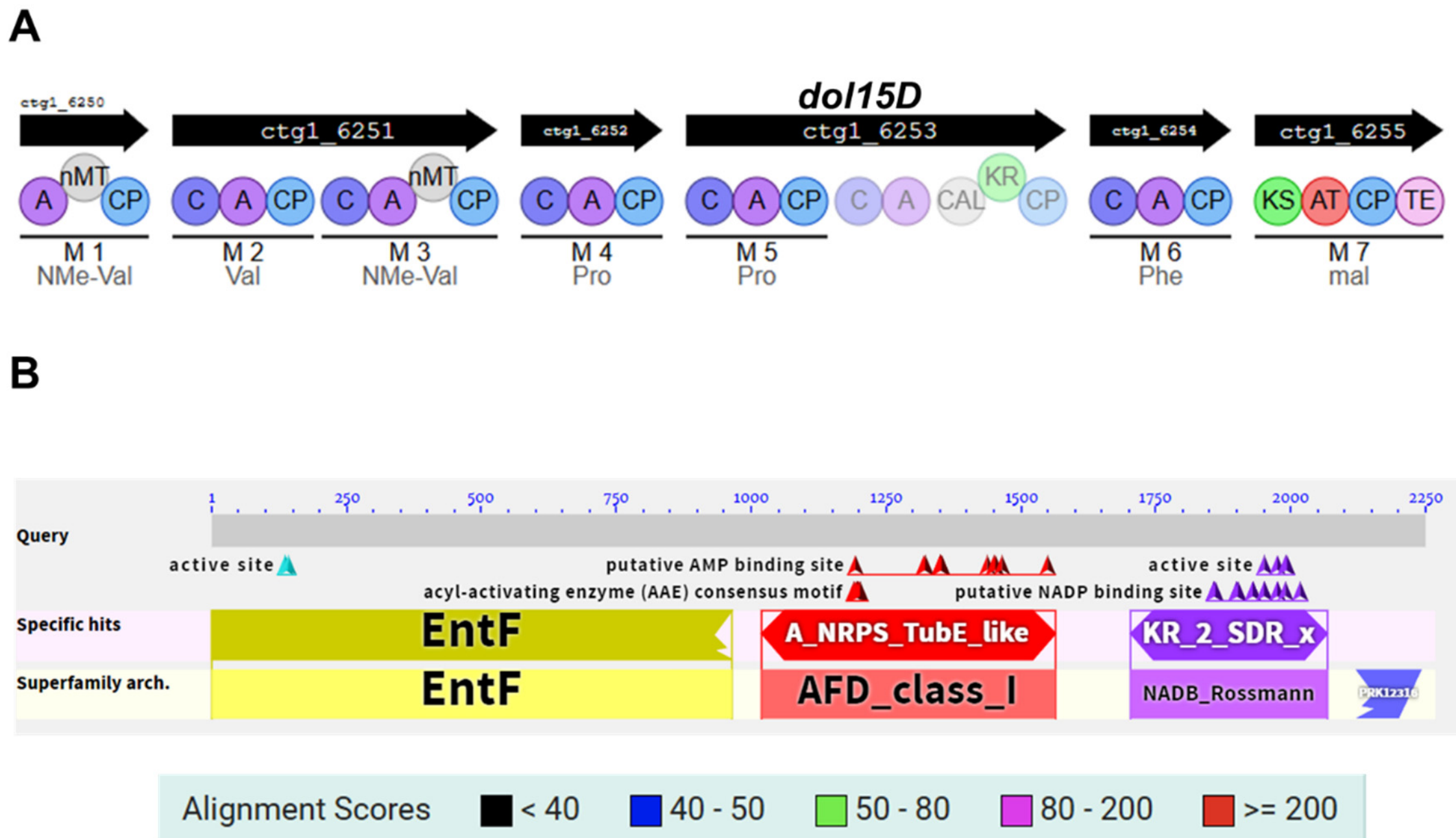

Fig. S5. Domain architecture of Dol15D-M2. (**A**) NRPS/PKS module prediction of the *dol15* BGC from *Dapis* sp. VPG23-77. (**B**) NCBI conserved domain analysis of Dol15D-M2. Of note, antiSMASH predicts the embedded domain as a CoA ligase (CAL).

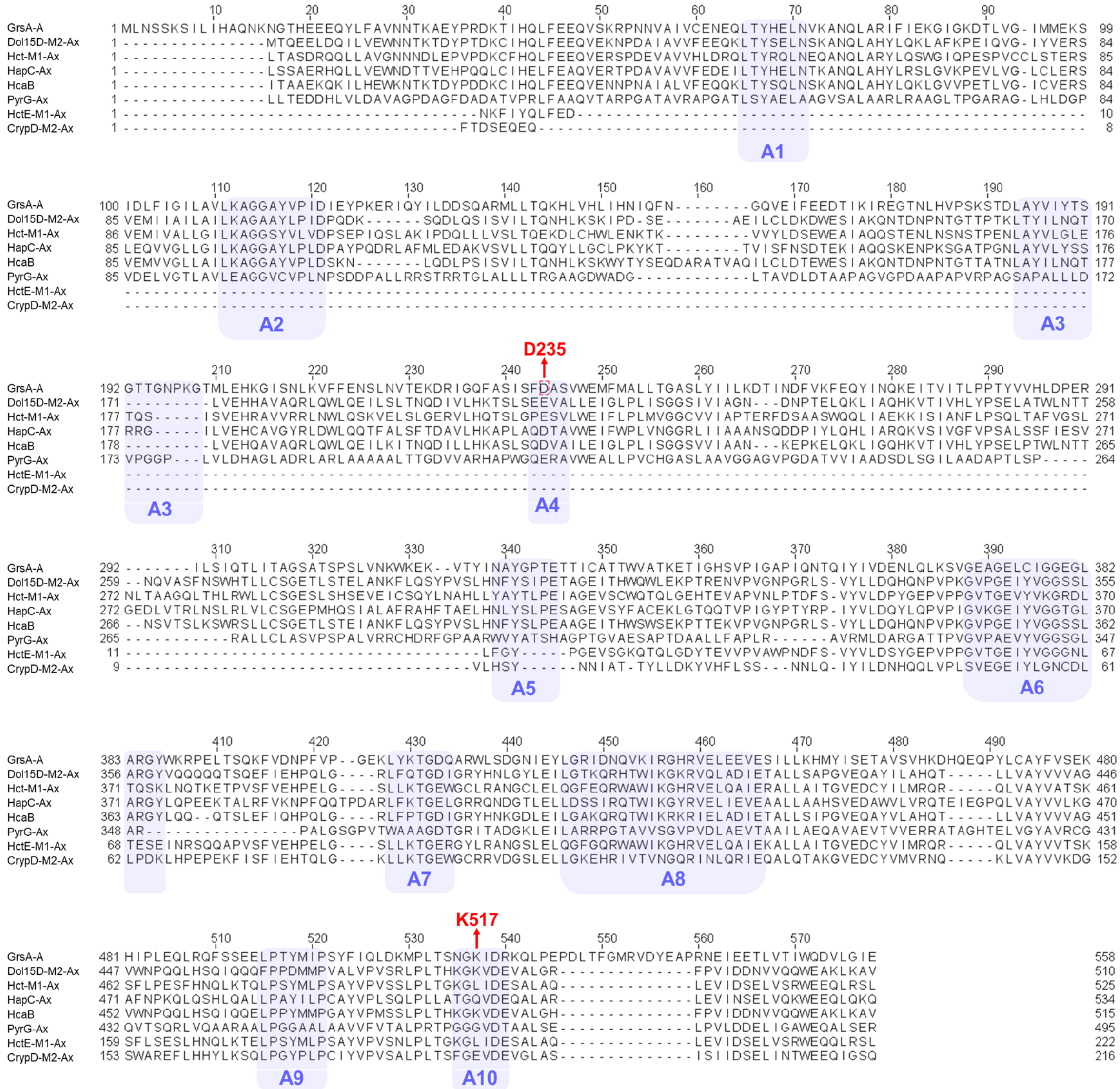

Fig. S6. Sequence alignment analysis shows the conserved A1-A10 motifs in the A_x and partial A_x domains of representative Dol15D-M2 and CrpD-M2 homologs.

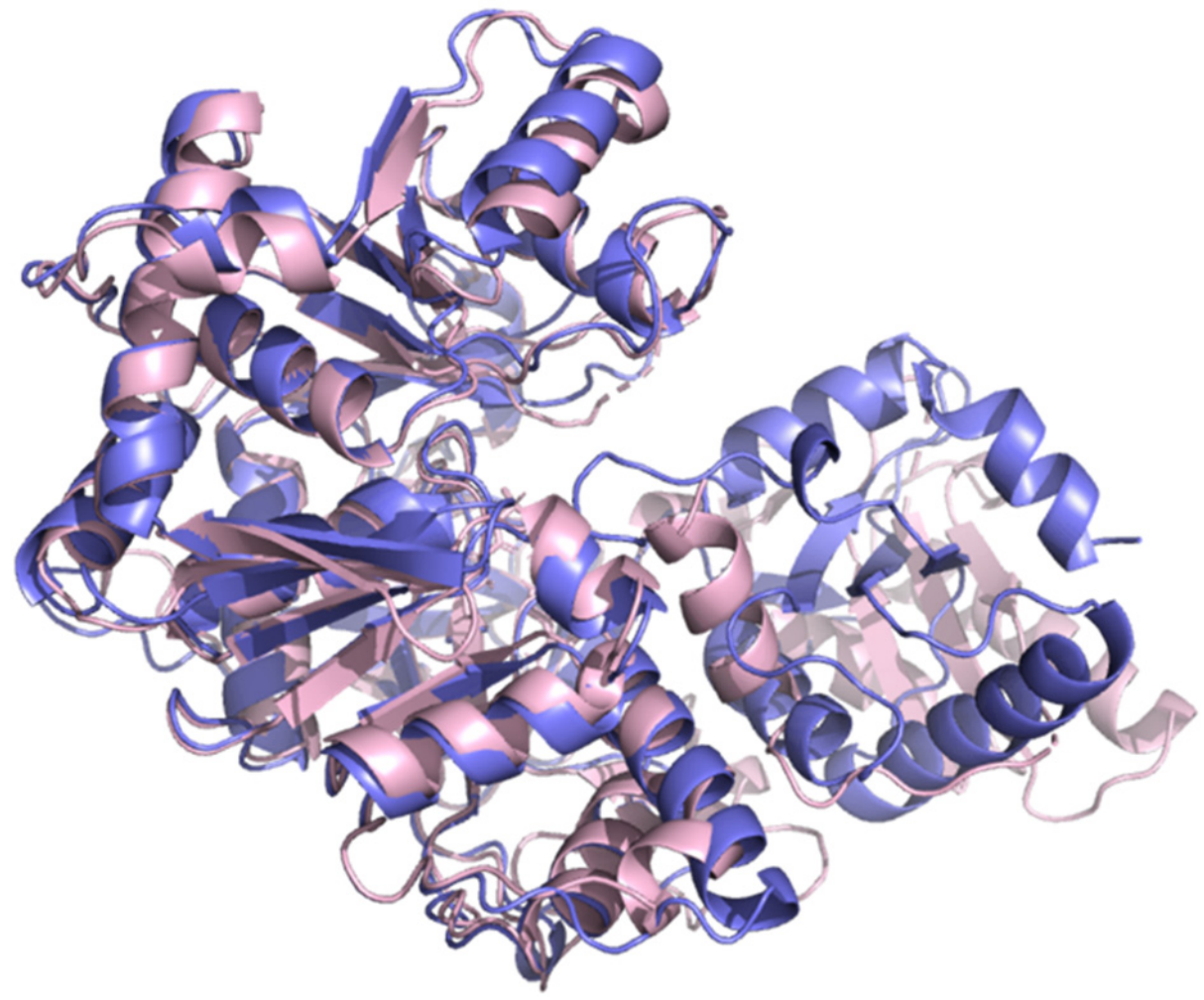

Fig. S7. Structure alignment of StsA-KAL (pink, PDB: 6ULX) with AlphaFold 3 predicted Dol15D-KAL (blue). RMSD = 0.9943 Å.

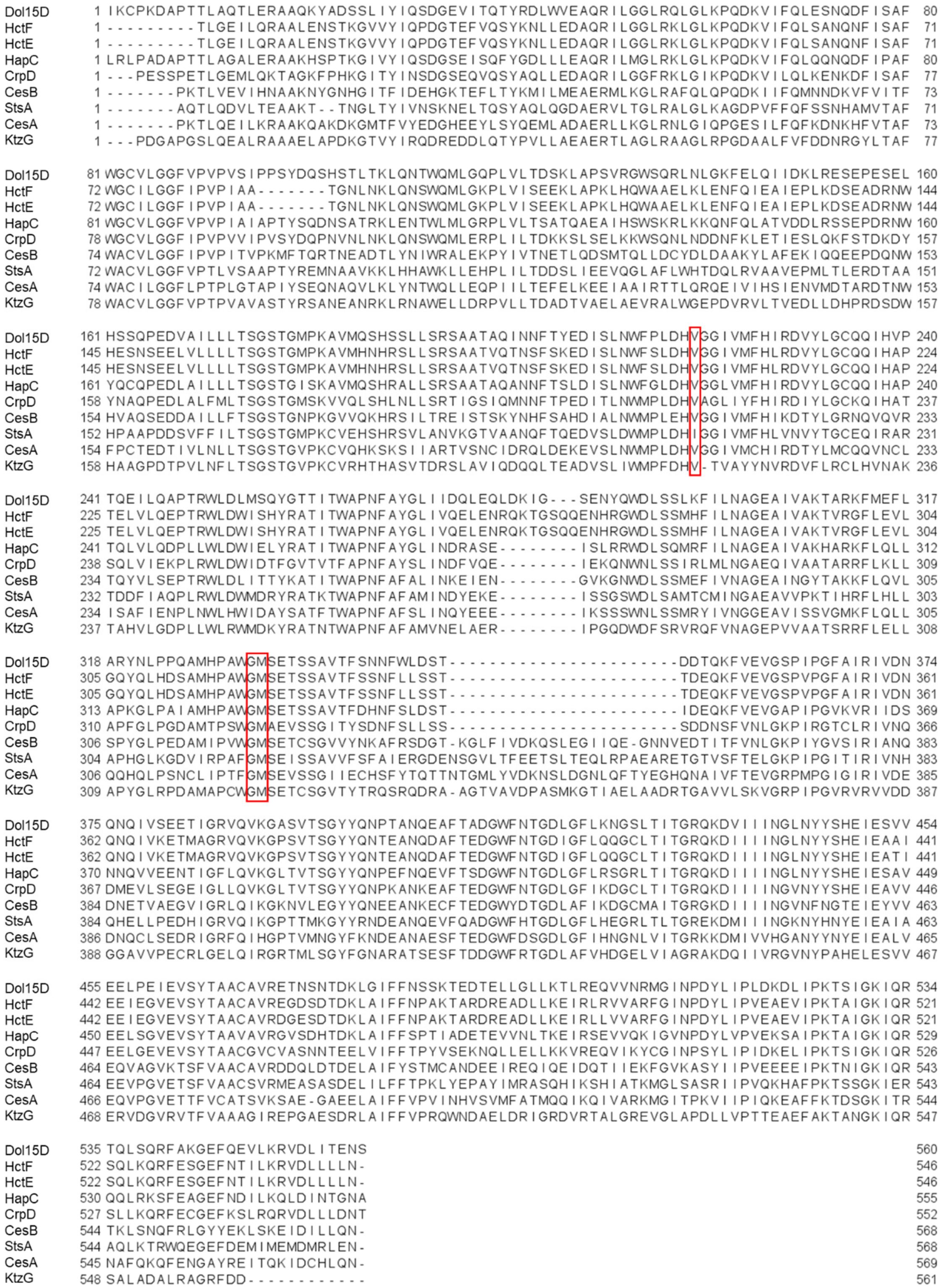

Fig. S8. Sequence alignment of Dol15D-M2 KAL and selected KAL domains confirmed or predicted to activate KIV or KIC. The conserved residue according to V219, G332 and M333 in Dol15D-M2 KAL are highlighted in the red box.

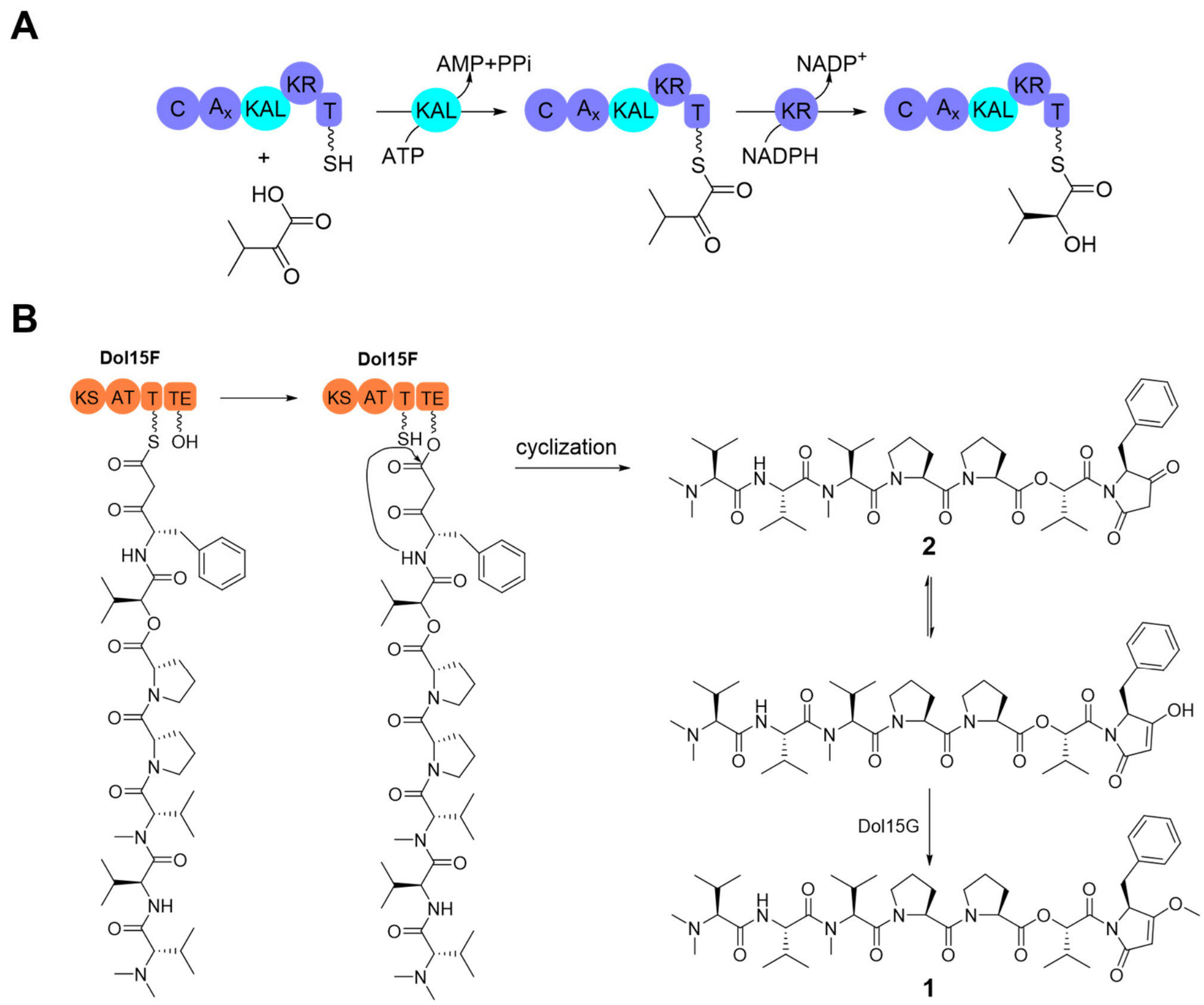

Fig. S9. Proposed biosynthetic sequence for hydroxy acid incorporation and methoxy pyrrolinone formation. (A) Proposed sequence of 2-keto acid activation by the KAL domain and ketoreduction to generate the corresponding hydroxyacyl intermediate for the ester bond formation. **(B)** Proposed sequence of TE-mediated tetramic acid formation, tautomerization between the diketone and enol forms, and *O*-methylation by Dol15G to generate the methoxy pyrrolinone moiety.

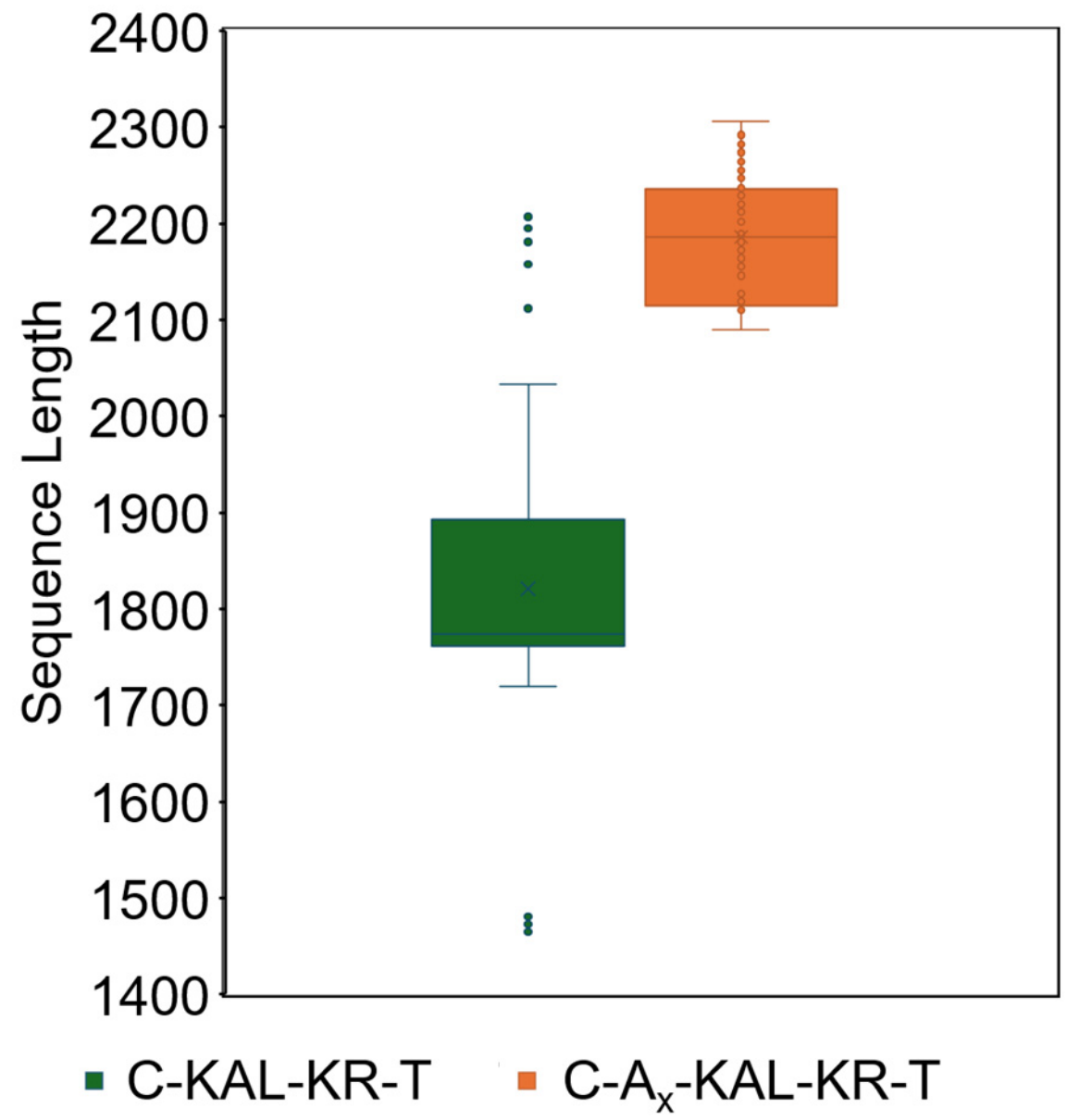

Fig. S10. Size distribution of identified C-A_x-KAL-KR-T and C-KAL-KR-T homologs.

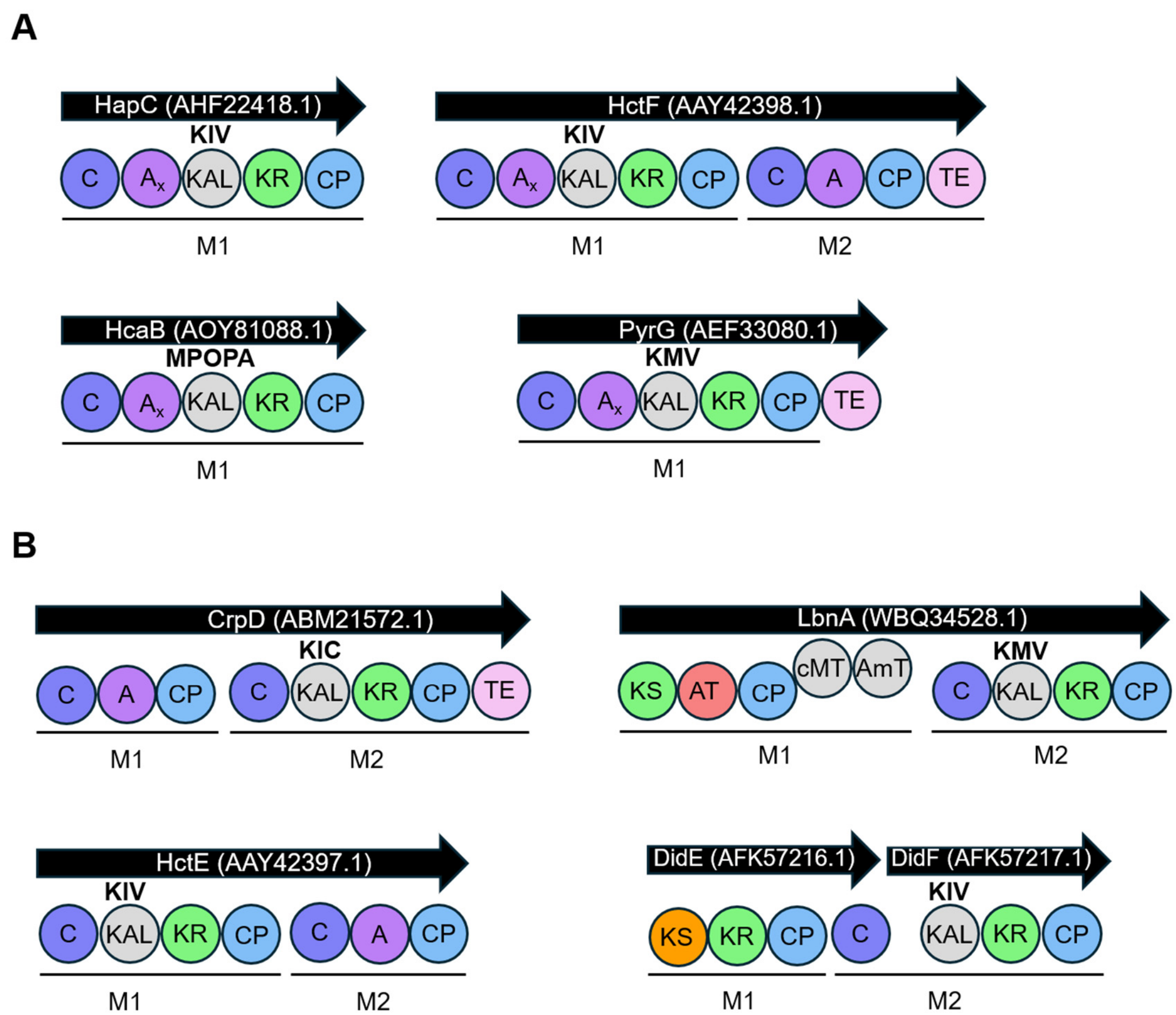

Fig. S11. Domain organization of selected NRPSs containing Dol15D-M2 homologs (A) or C-KAL-KR-T modules (B).

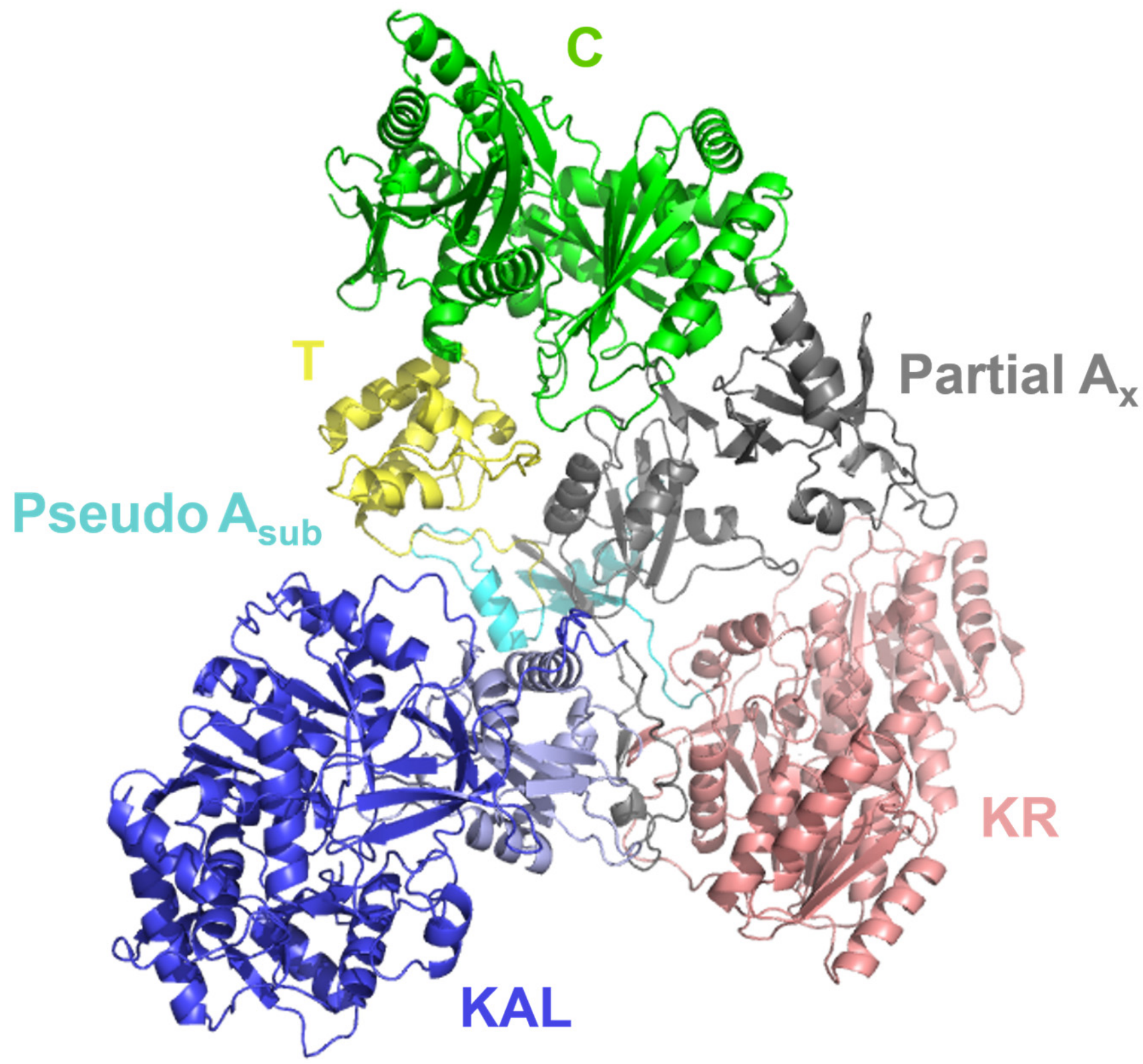

Fig. S12. Predicted structure of CrpD-M2 by AlphaFold 3. A partial A_x-like region is retained in CrpD-M2 (grey).

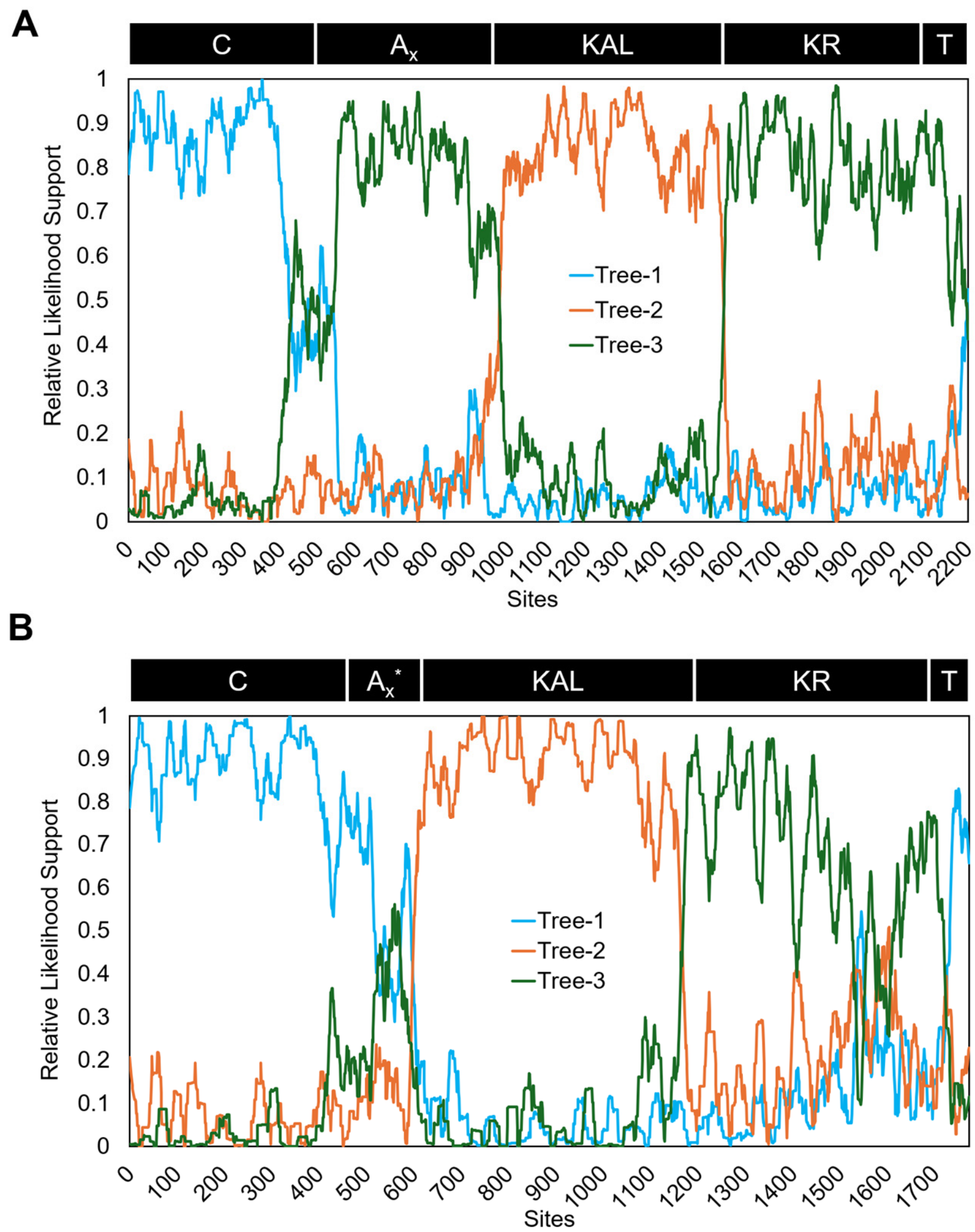

Fig. S13. Comparative phylogenetic analysis of 125 Dol15D-M2 (A) and 337 CrpD-M2 homologs (B) using a three-tree model reveals recombination insertion of a KAL domain between the C-A_x or C-A_x^* and KR-T segments. A_x^* represents partial A_x domain.

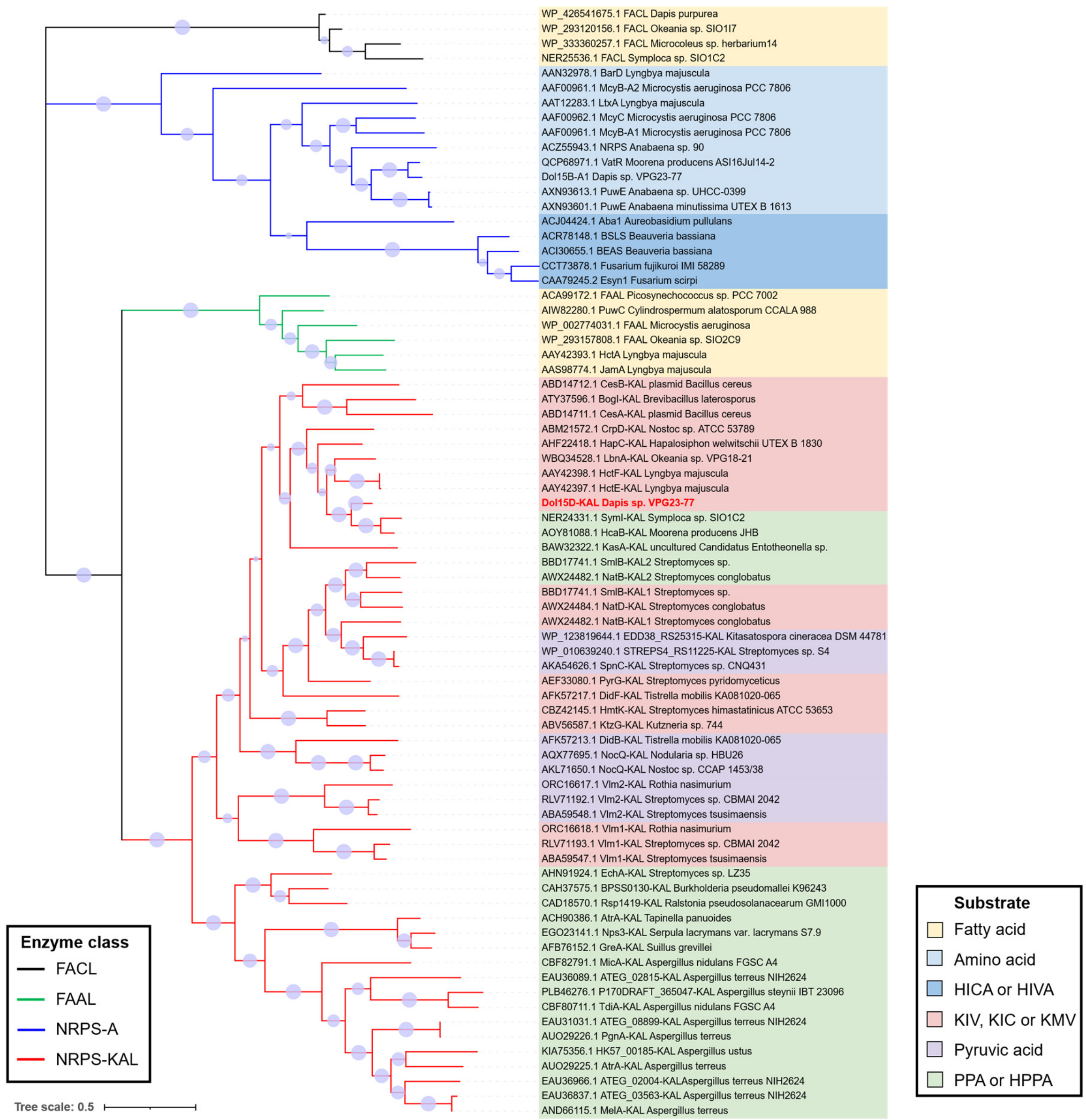

Fig. S14. Phylogenic tree of KAL domains with other members in the ANL family. The branches were colored according to the enzyme classes, and the label background was colored according to the proposed substrates. FACL: fatty acyl-CoA ligase; FAAL: fatty acyl-AMP ligase; NRPS-KAL: 2-keto acid AMP ligase domain present in a NRPS module; NRPS-A: canonical NRPS A domains; HICA: 2-hydroxyisocaproic acid; HIVA 2-hydroxyisovaleric acid; KIV: 2-ketoisovaleric acid; KIC: 2-ketoisocaproic acid; KMV: 2-keto-3-methylvaleric acid; PPA: phenylpyruvic acid; HPPA: 4-hydroxyphenylpyruvic acid.

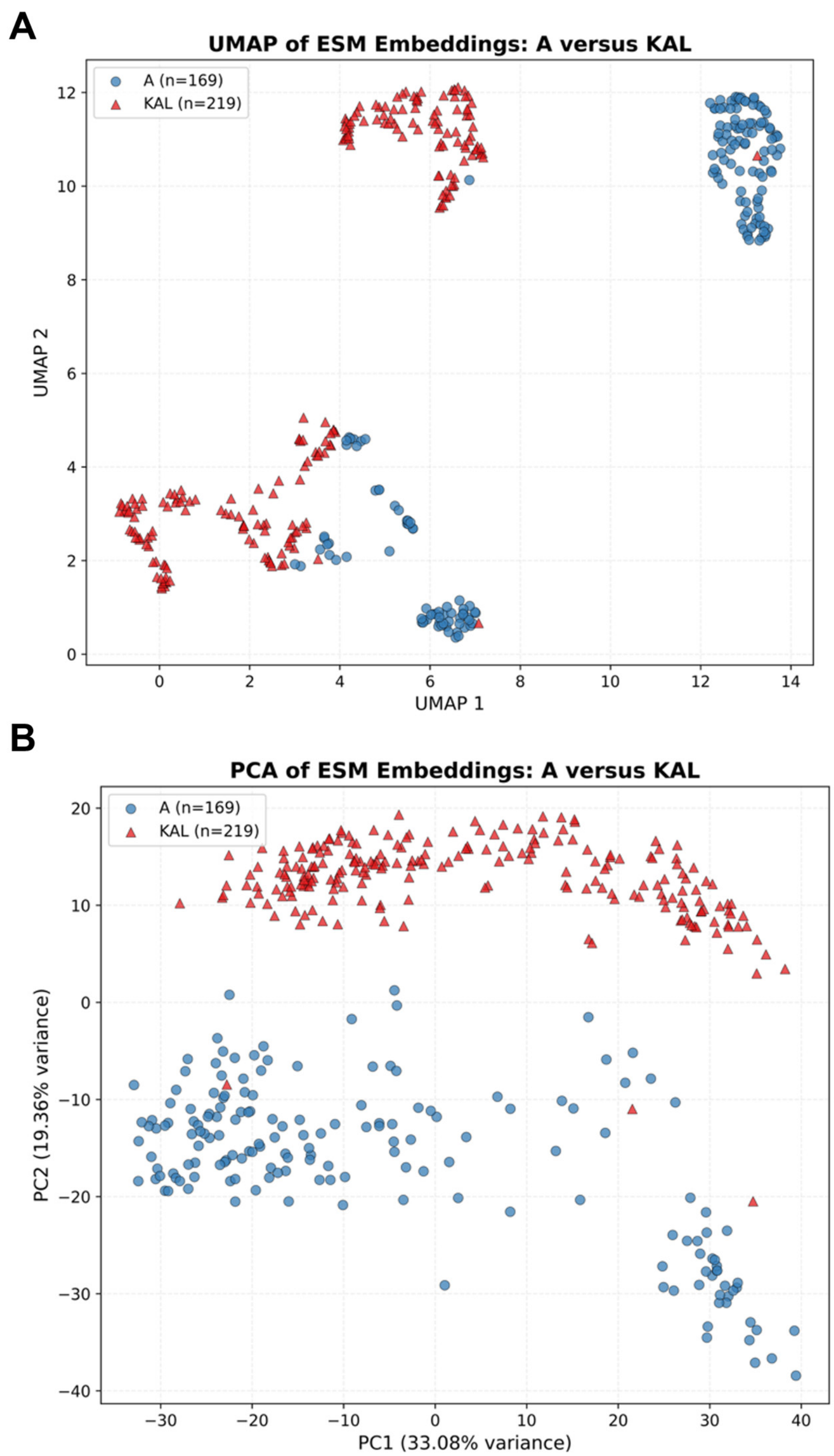

Fig. S15. Visualization of ESM2 embedding space distinguishes KAL and canonical NRPS adenylation domains. (A) Uniform Manifold Approximation and Projection (UMAP) visualization of ESM2 embeddings for a nonredundant dataset (≤85% sequence identity) comprising 219 KAL domains and 169 canonical NRPS A domains. **(B)** Principal component analysis (PCA) of the same ESM2 embeddings. In both analyses, KAL and canonical A domains form largely distinct clusters, supporting their separation based on sequence-derived protein language model embeddings.

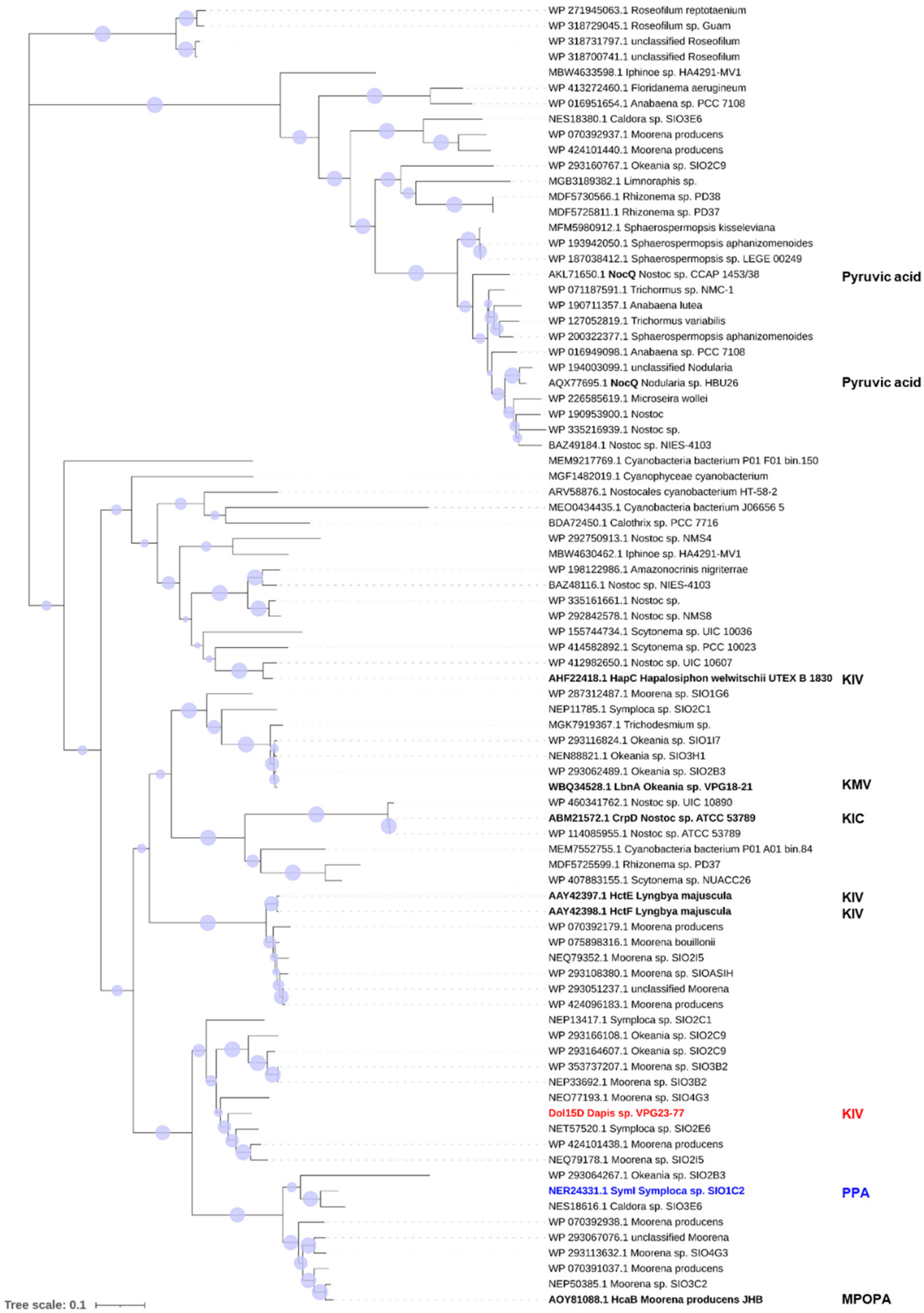

Fig. S16. Phylogenic tree of KAL domains identified from cyanobacteria. The homologs with known substrates were highlighted in bold. KIV: 2-ketoisovaleric acid; KIC: 2-ketoisocaproic acid; KMV: 2-keto-3-methylvaleric acid; PPA: phenylpyruvic acid; MPOPA: 3-(4-methoxyphenyl)-2-oxopropanoic acid.

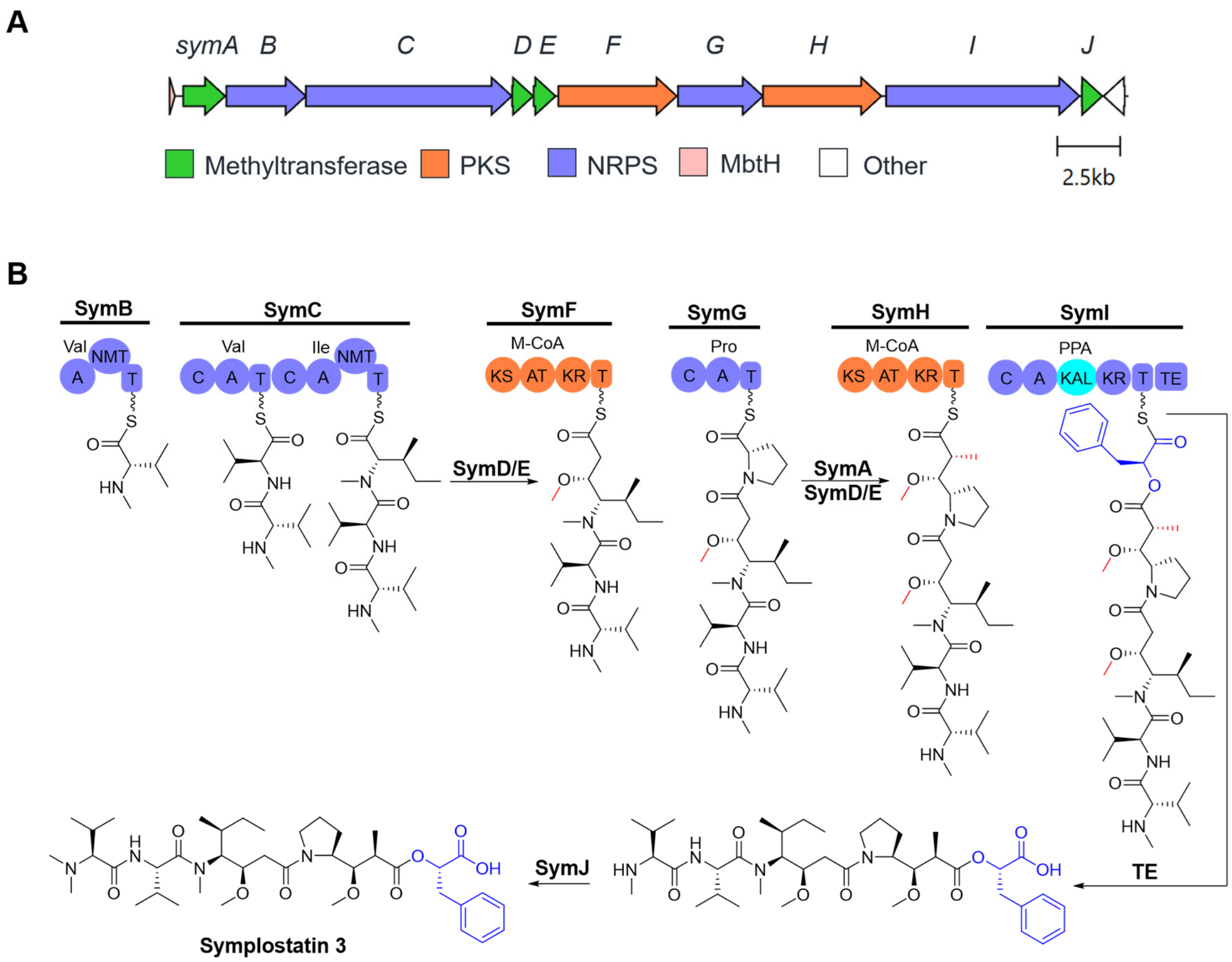

Fig. S17. Proposed biosynthetic pathway of symplostatin 3. (**A**) A putative dolastatin-type BGC from *Symploca* sp. SIO1C2 (GenBank accession: JAAHFP010000047.1). (**B**) Proposed symplostatin 3 biosynthetic route. M-CoA: malonyl-CoA; PPA: phenylpyruvic acid.

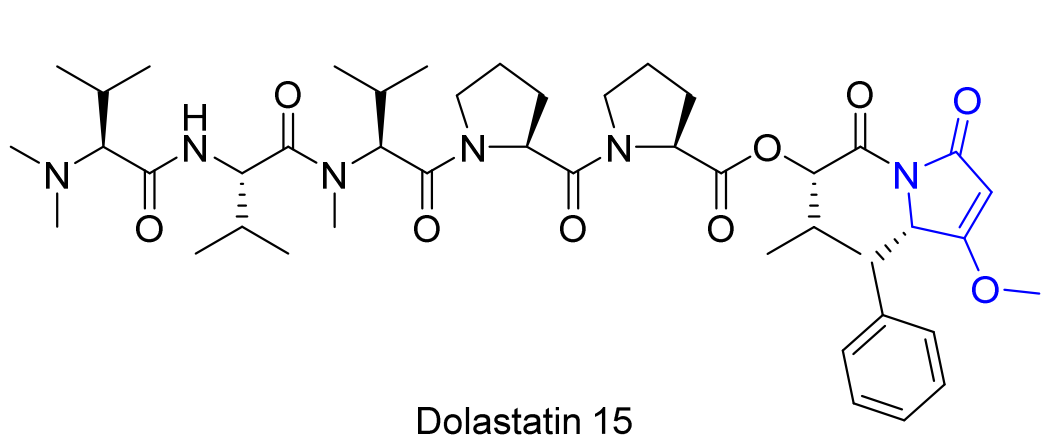

Dolastatin 15

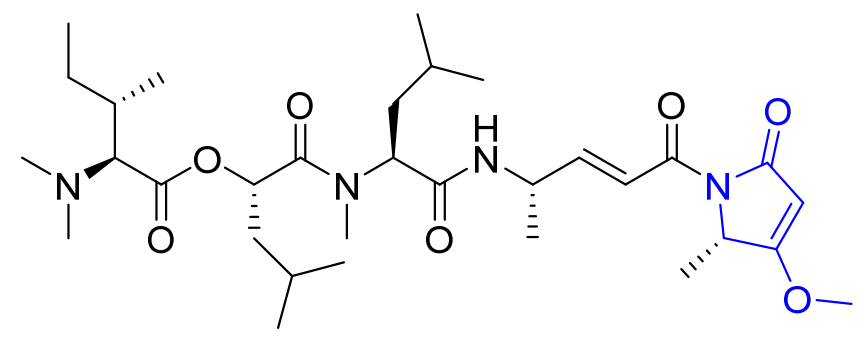

Symplostatin 4

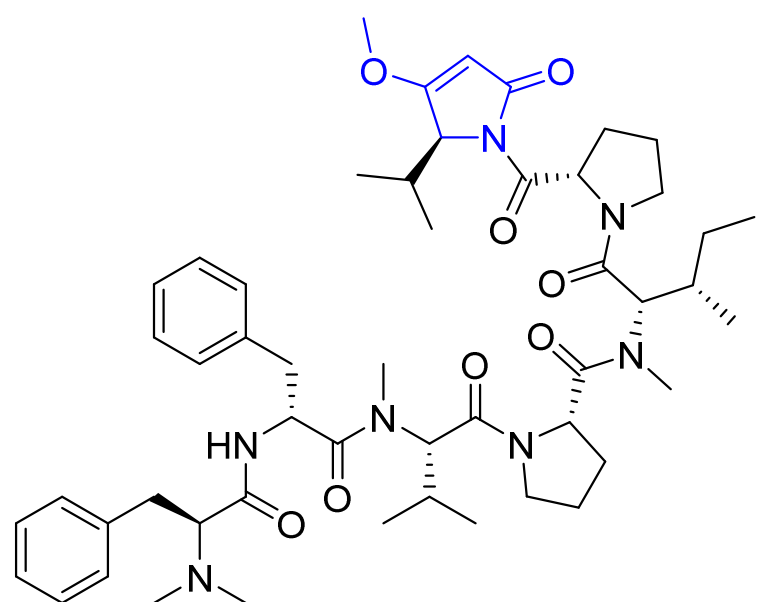

Iheyamide A

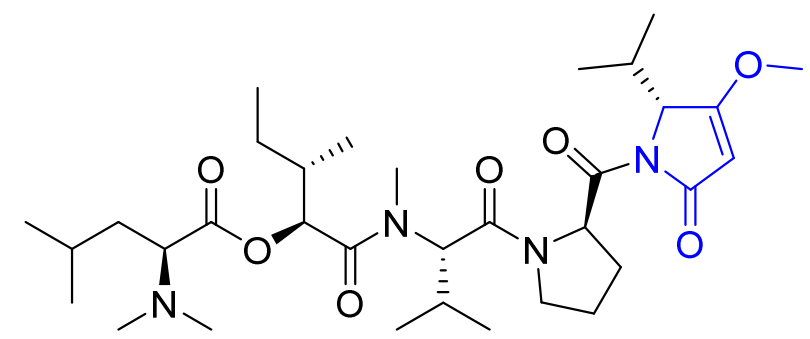

Mirabimide A

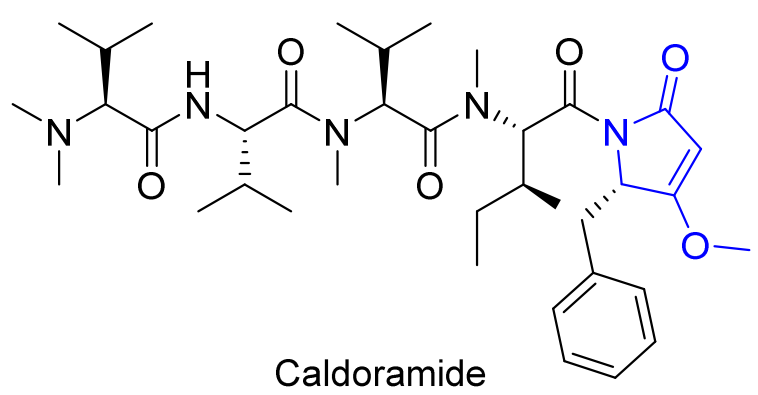

Caldoramide

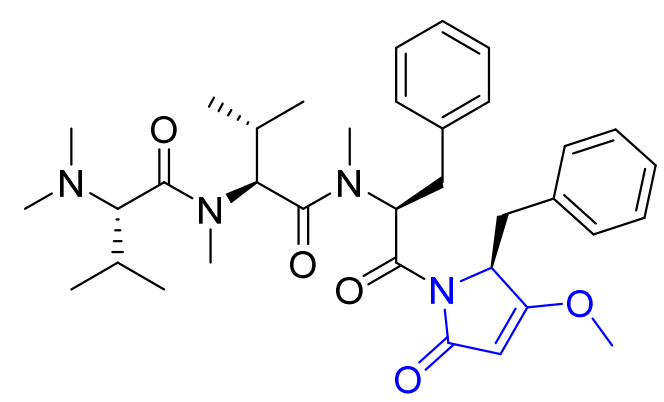

Belamide A

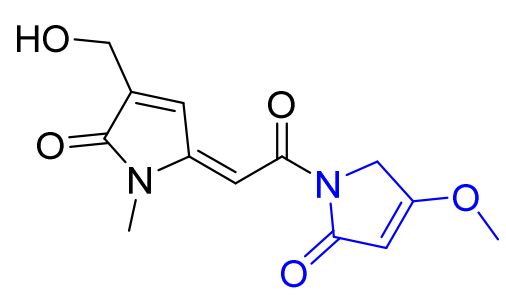

Pukeleimide A

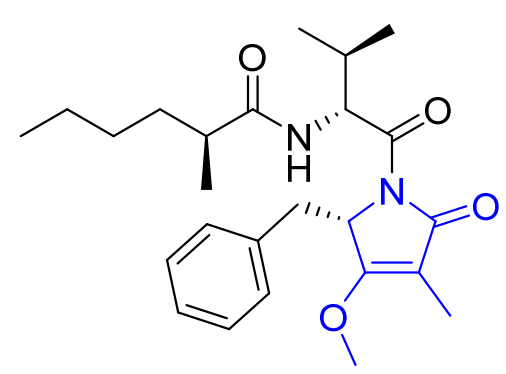

Palau'imide

Fig. S18. Representative cyanobacteria natural products containing C-terminal *N*-acylated pyrrolidine-2,4-dione.

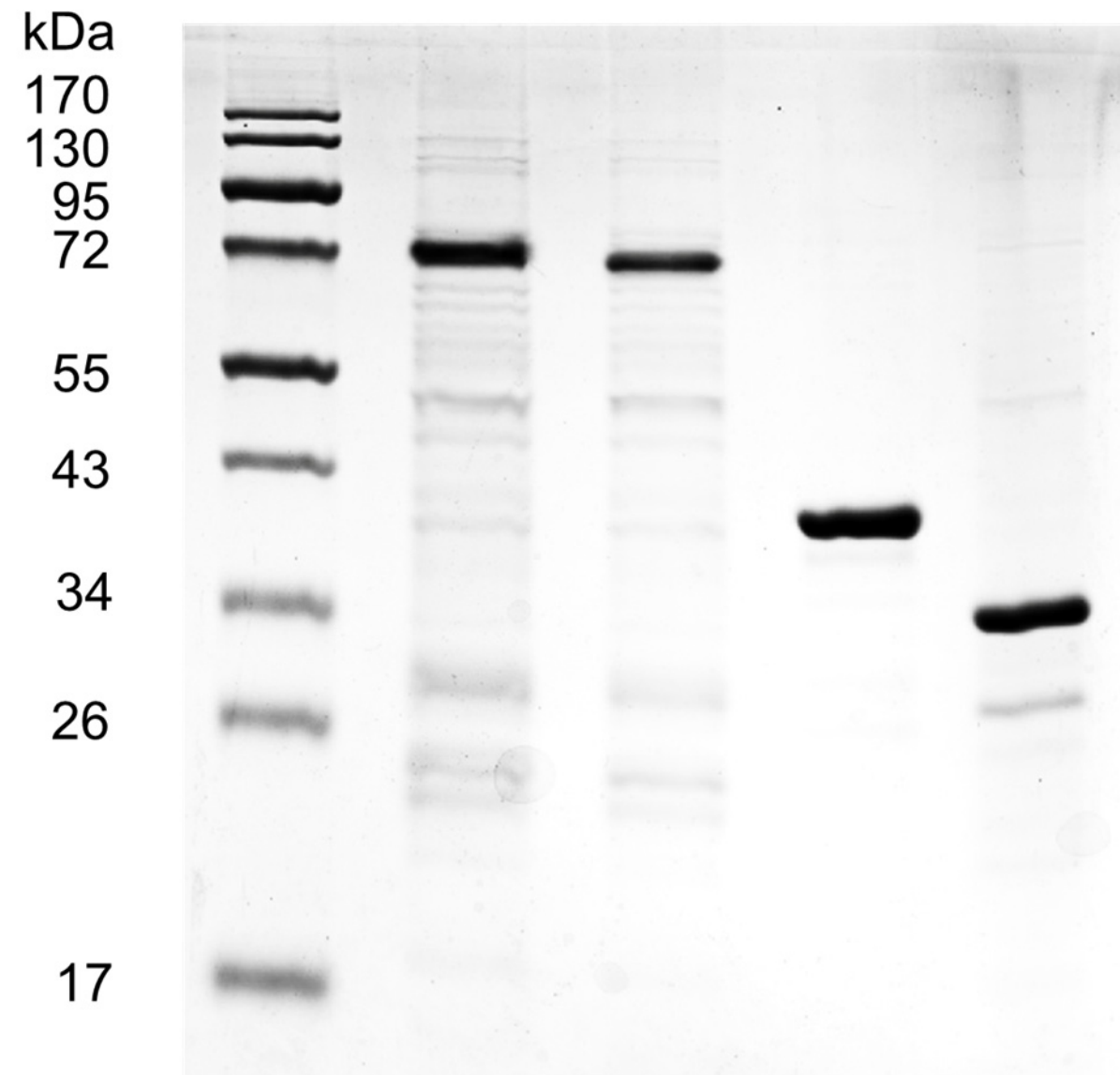

Fig. S19. SDS-PAGE of recombinant proteins. From left to right: Dol15D-M2 KAL WT, V219D variant, Dol15G, and Dol15F-TE. Expected molecular weight of the recombinant proteins: Dol15D-M2 KAL at 72.2 kDa; Dol15G at 39.1 kDa; Dol15F-TE at 33.0 kDa.

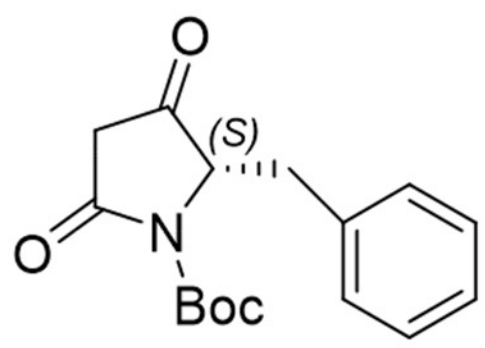

3

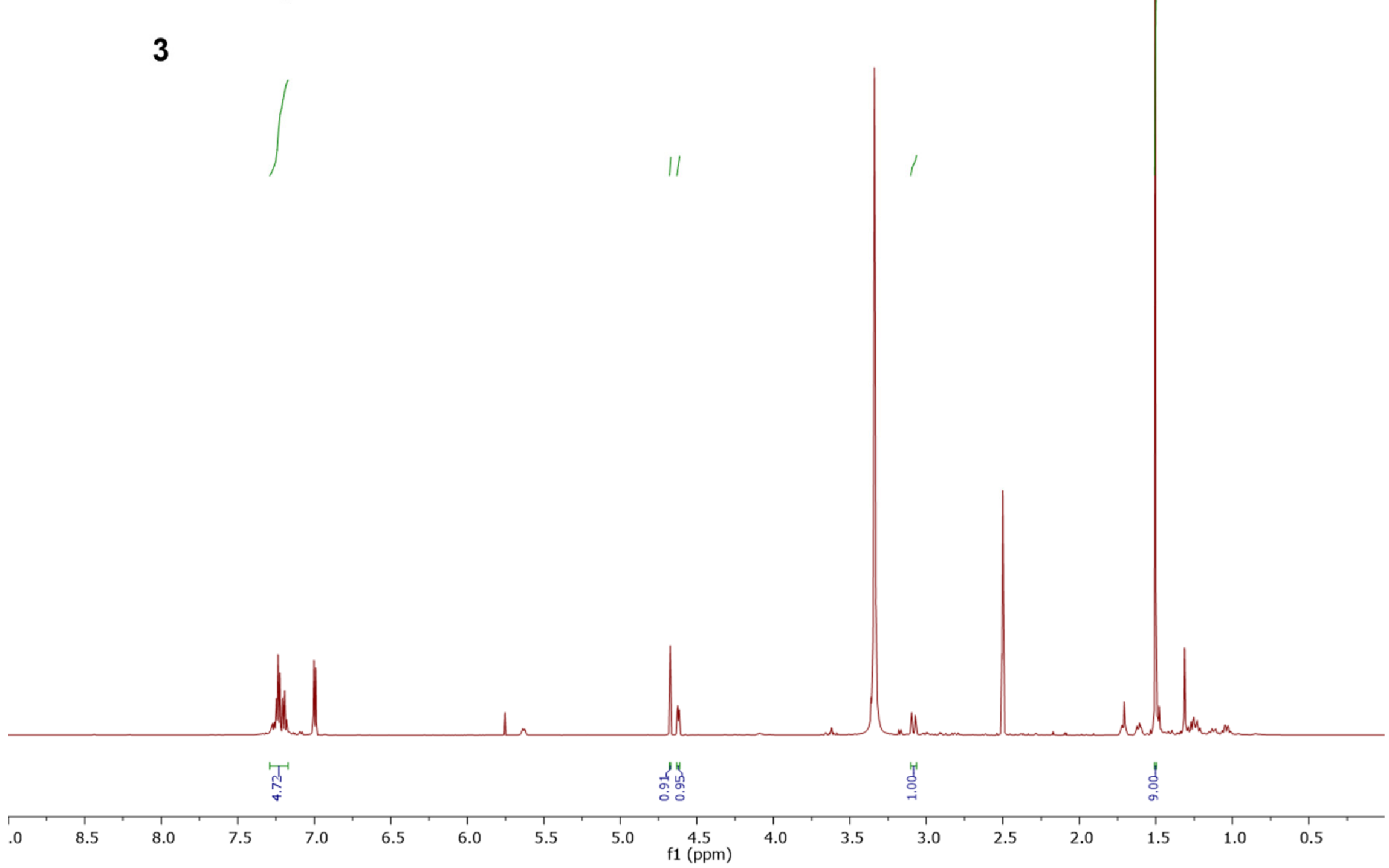

Fig. S20. ¹H NMR spectrum of crude synthetic 3 in DMSO-*d*₆ (600 MHz) at 25 °C.

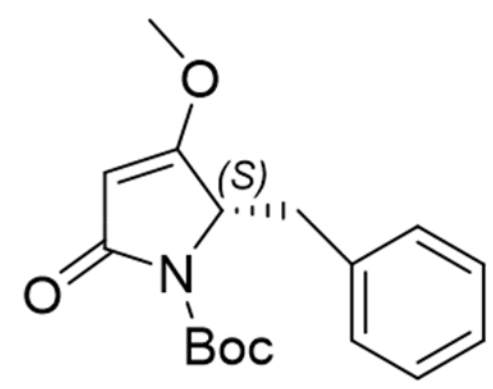

4

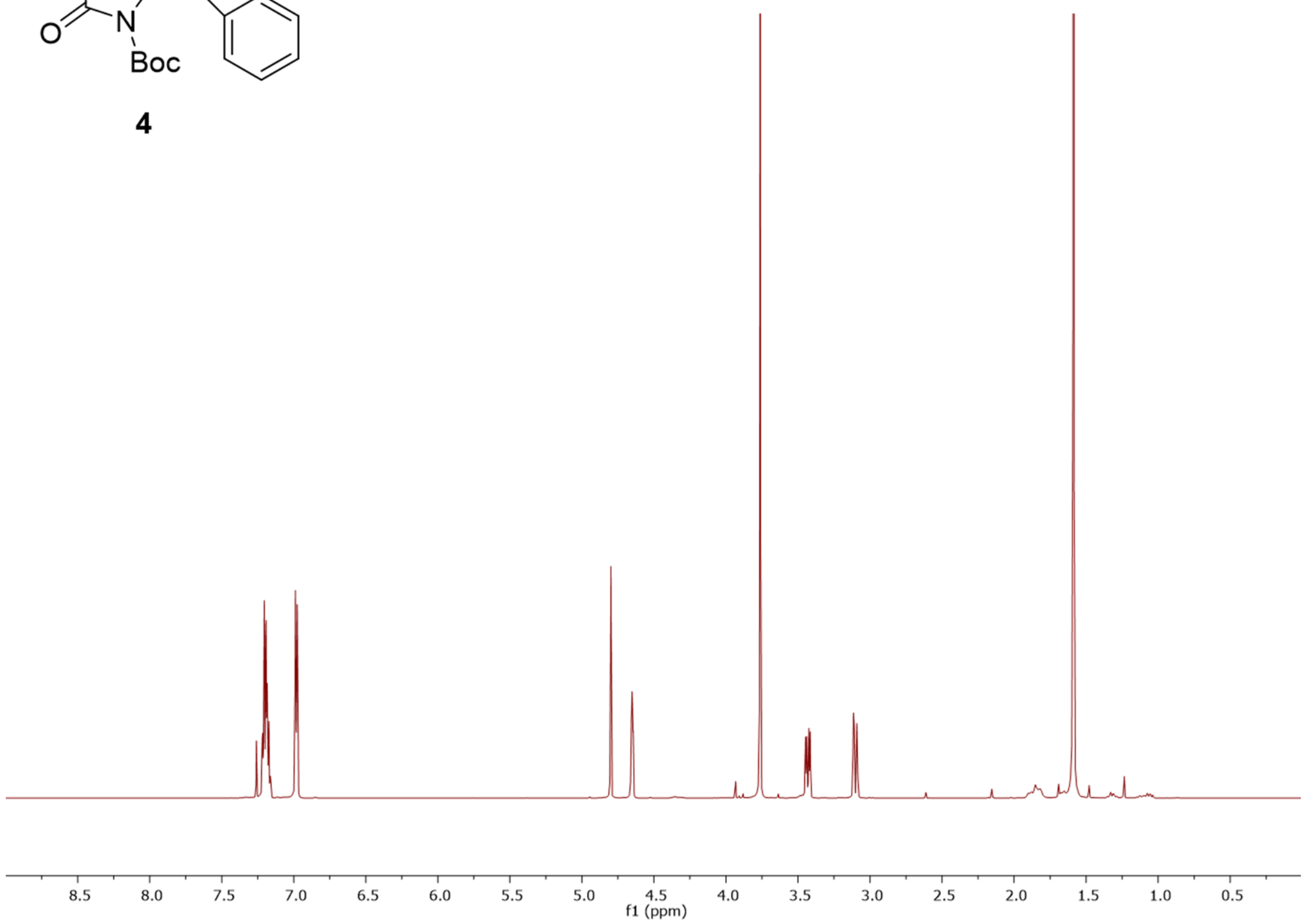

Fig. S21. ¹H NMR spectrum of synthetic 4 in $CDCl_3$ (600 MHz) at 25 °C.

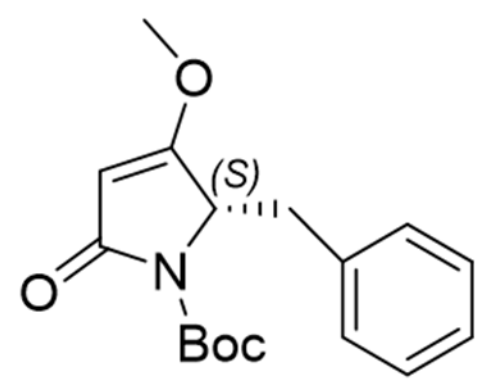

4

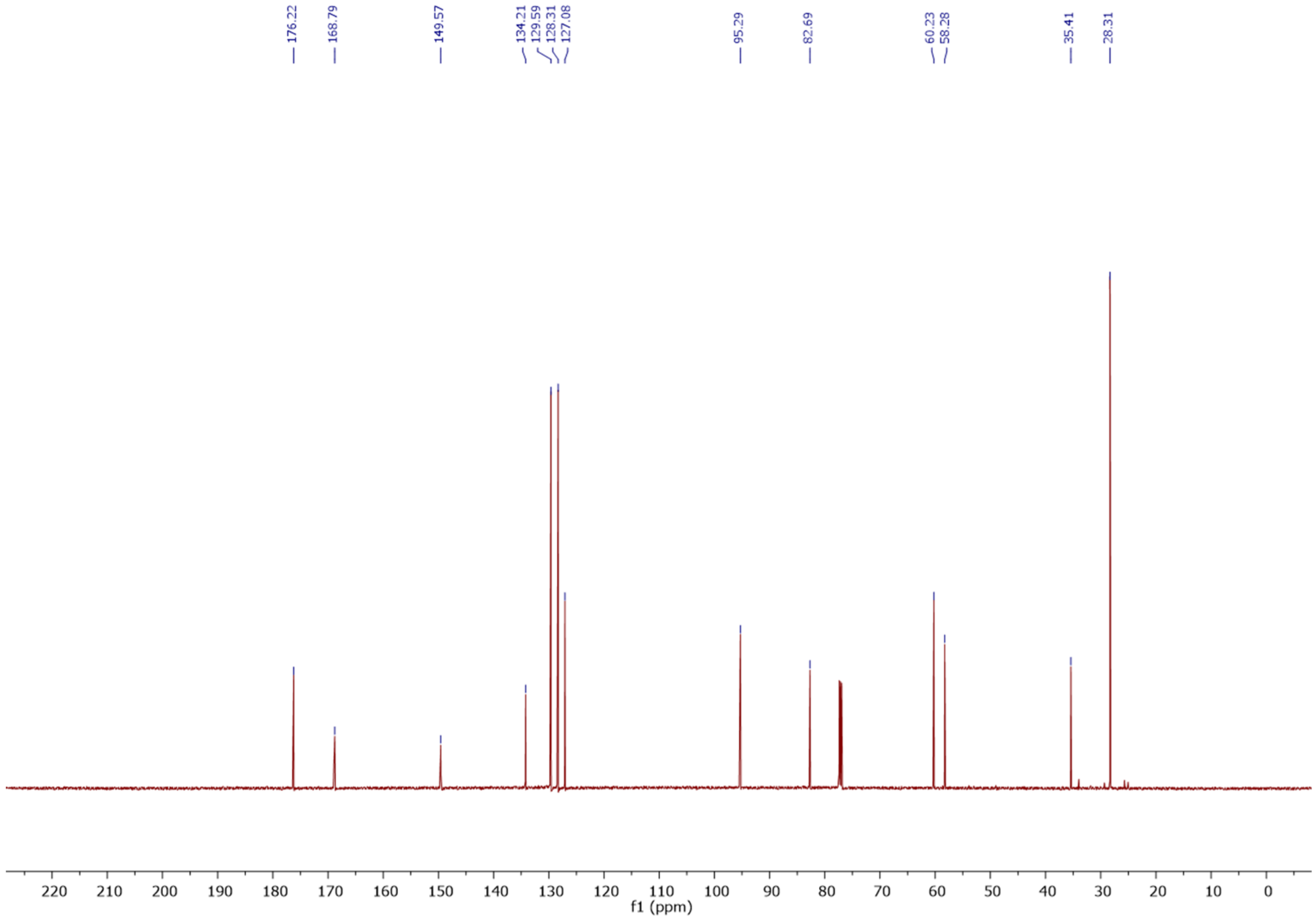

Fig. S22. ^{13}C NMR spectrum of synthetic 4 in $CDCl_3$ (150 MHz) at 25 °C.

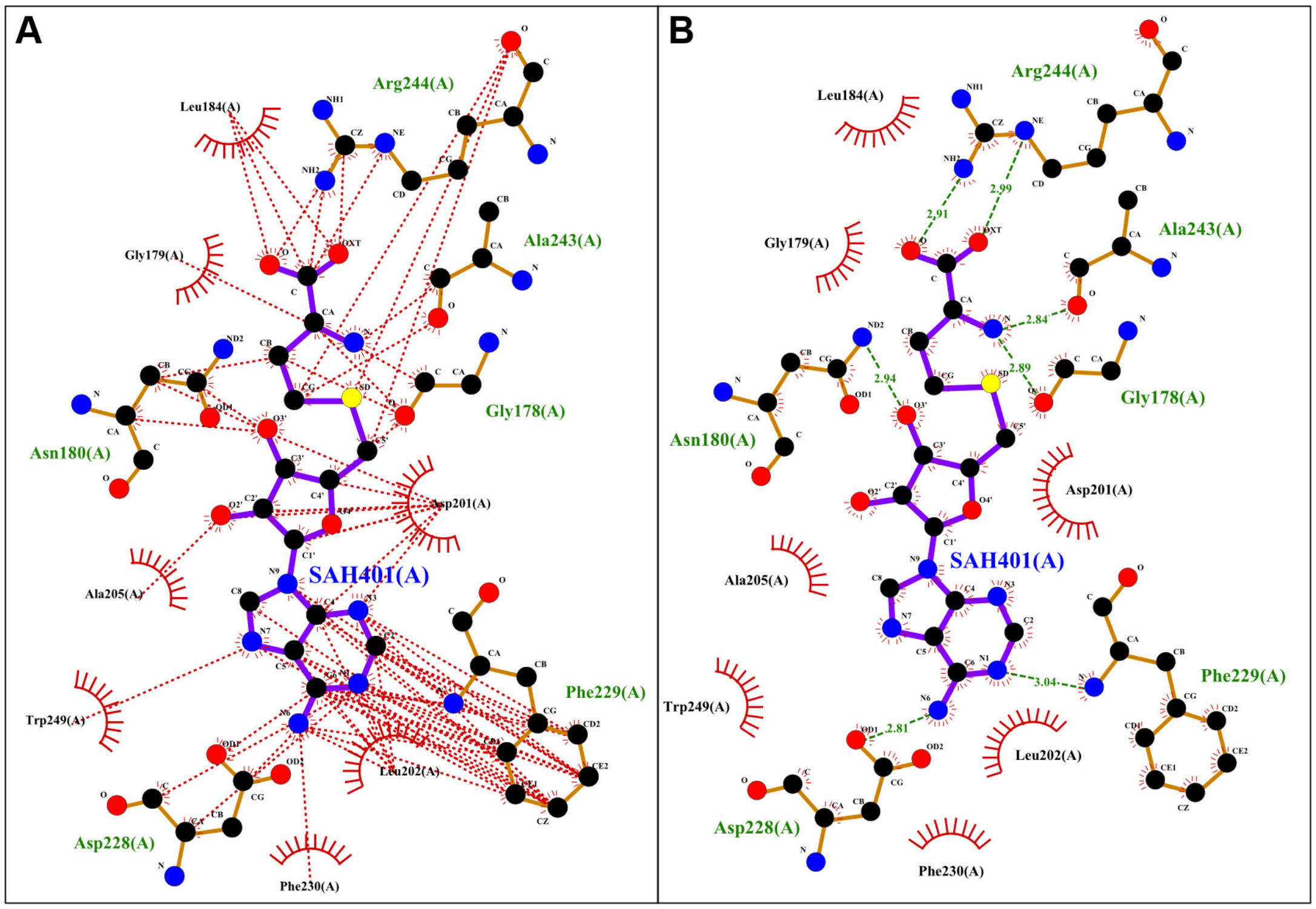

Fig. S23. Ligplot of SAH in Dol15G.

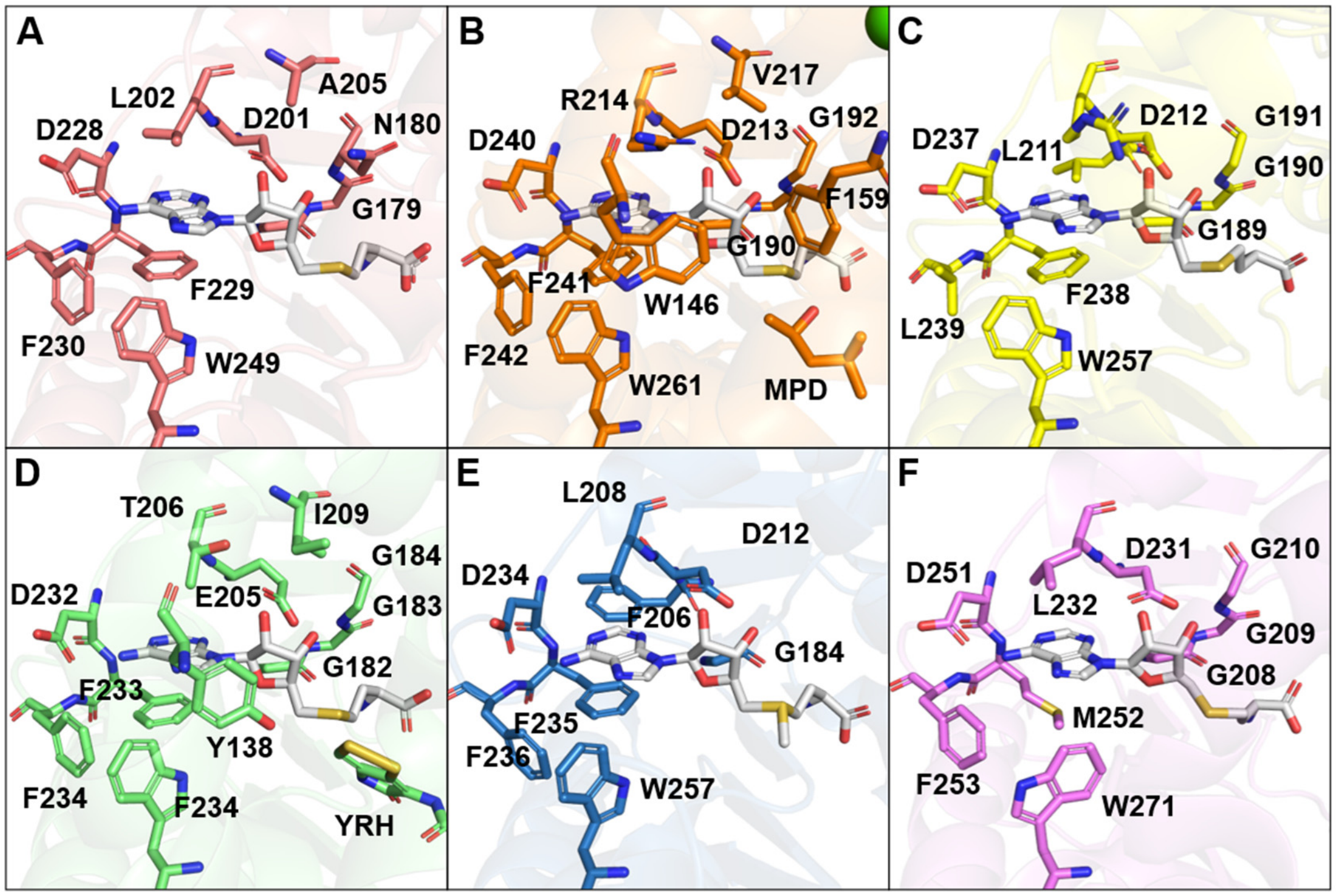

Fig. S24. Structural comparison of different O-methyltransferase homologs (A) Dol15G, (B) 3GWZ, (C) 3LST, (D) 8RDN, (E) 8TJJ, and (F) 1KYW. SAH or SAM within each structure are shown in gray. YRH: ligand of interest in 8RDN.

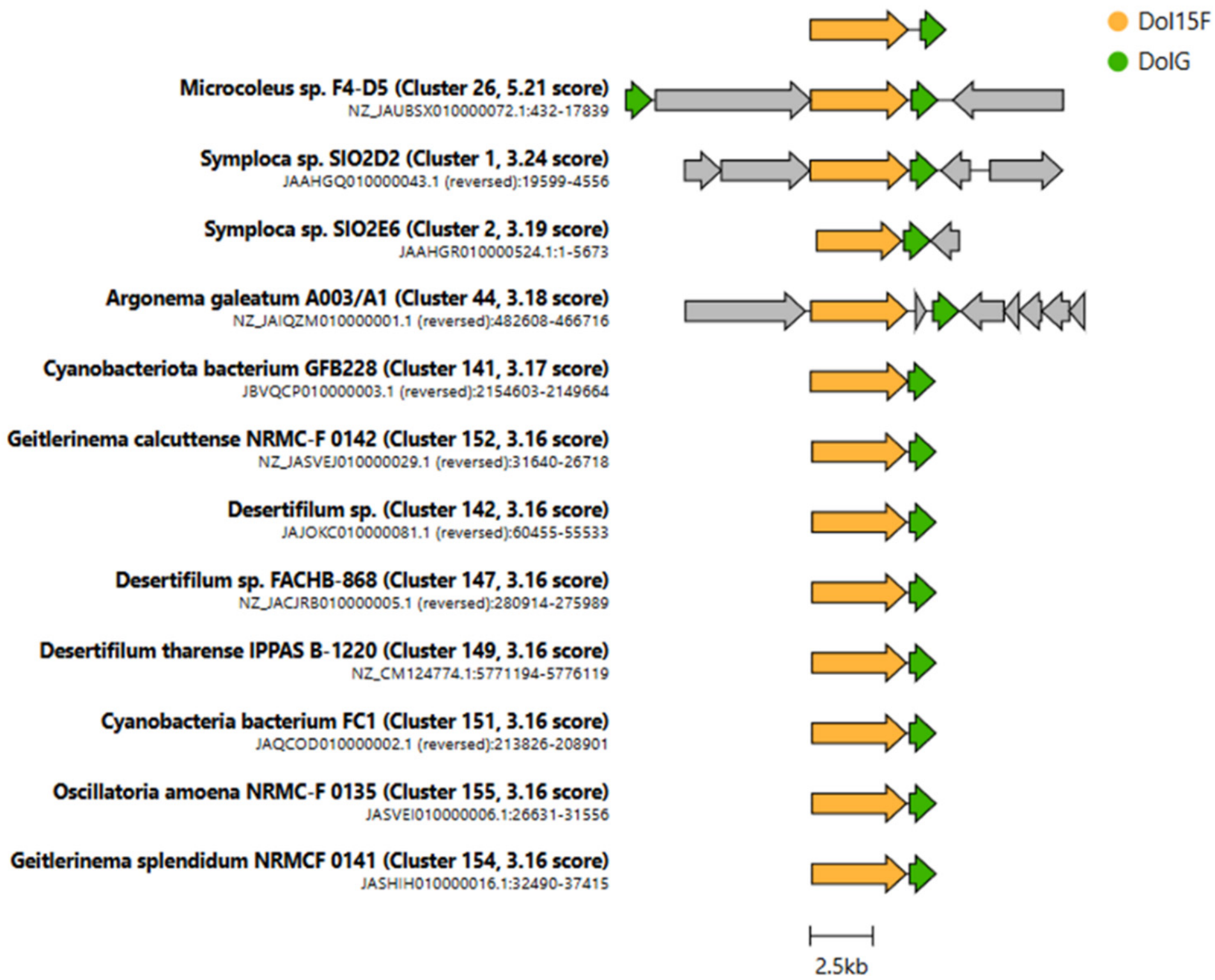

Fig. S25. Bioinformatic analysis revealed frequent co-localization of genes encoding Dol15F-G. Query of the Dol15F-G subcluster against the cyanobacteria genomes revealed hits containing co-localized dol15F-G homologs across diverse cyanobacteria genus.

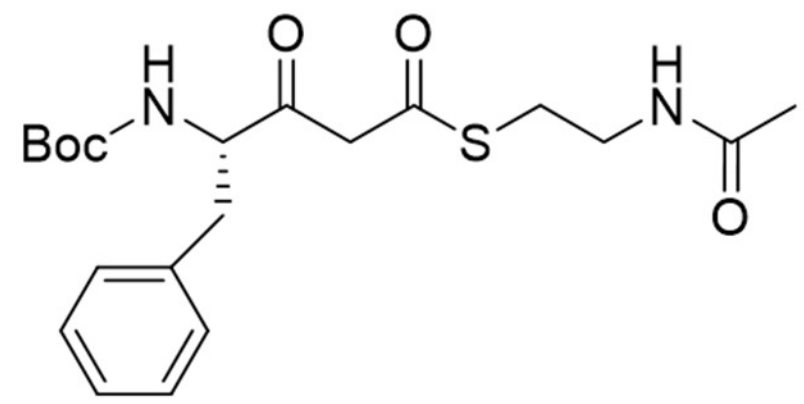

5

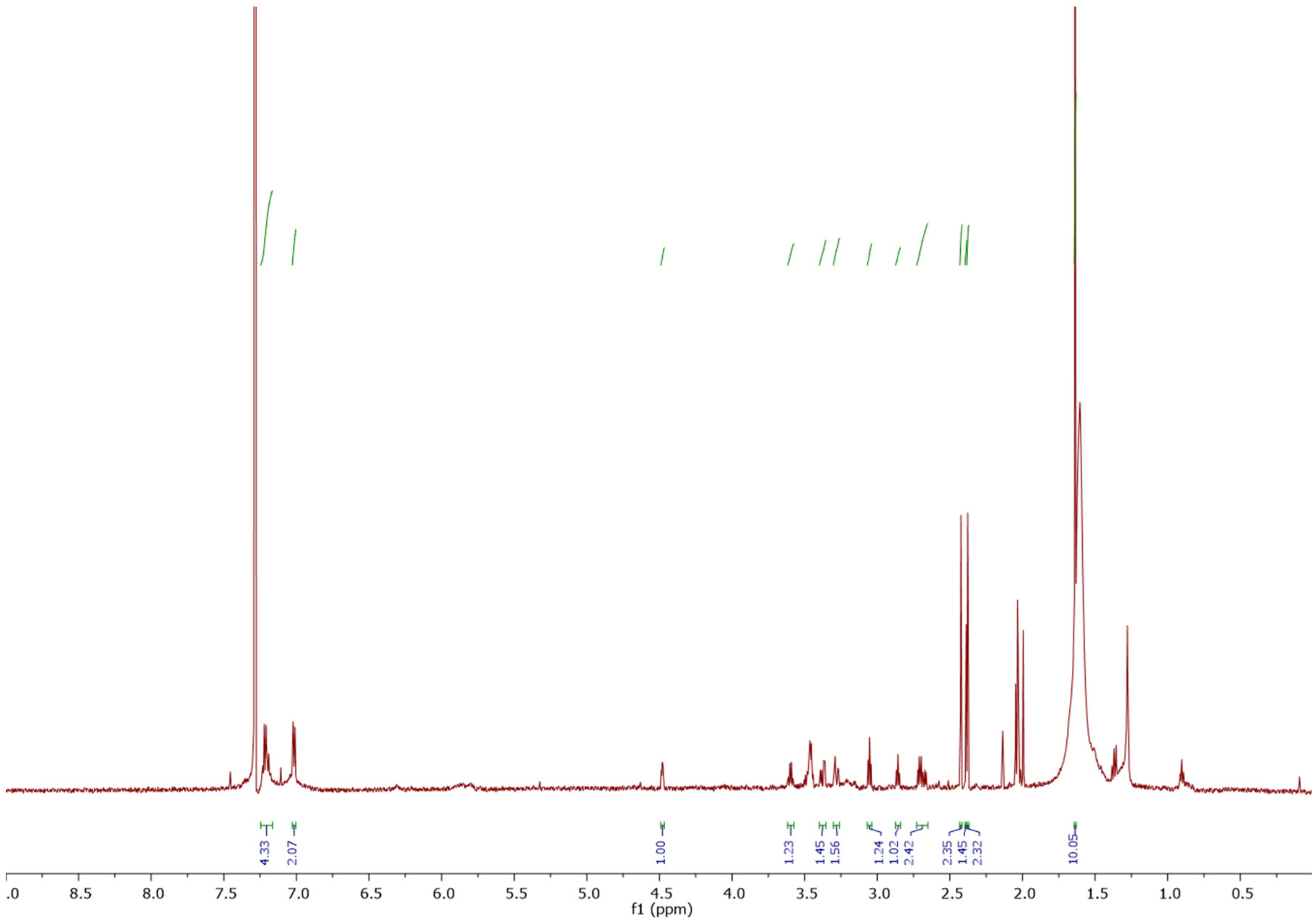

Fig. S26. ^{1}H NMR spectrum of crude synthetic 5 in $CDCl_3$ (600 MHz) at 25 °C.

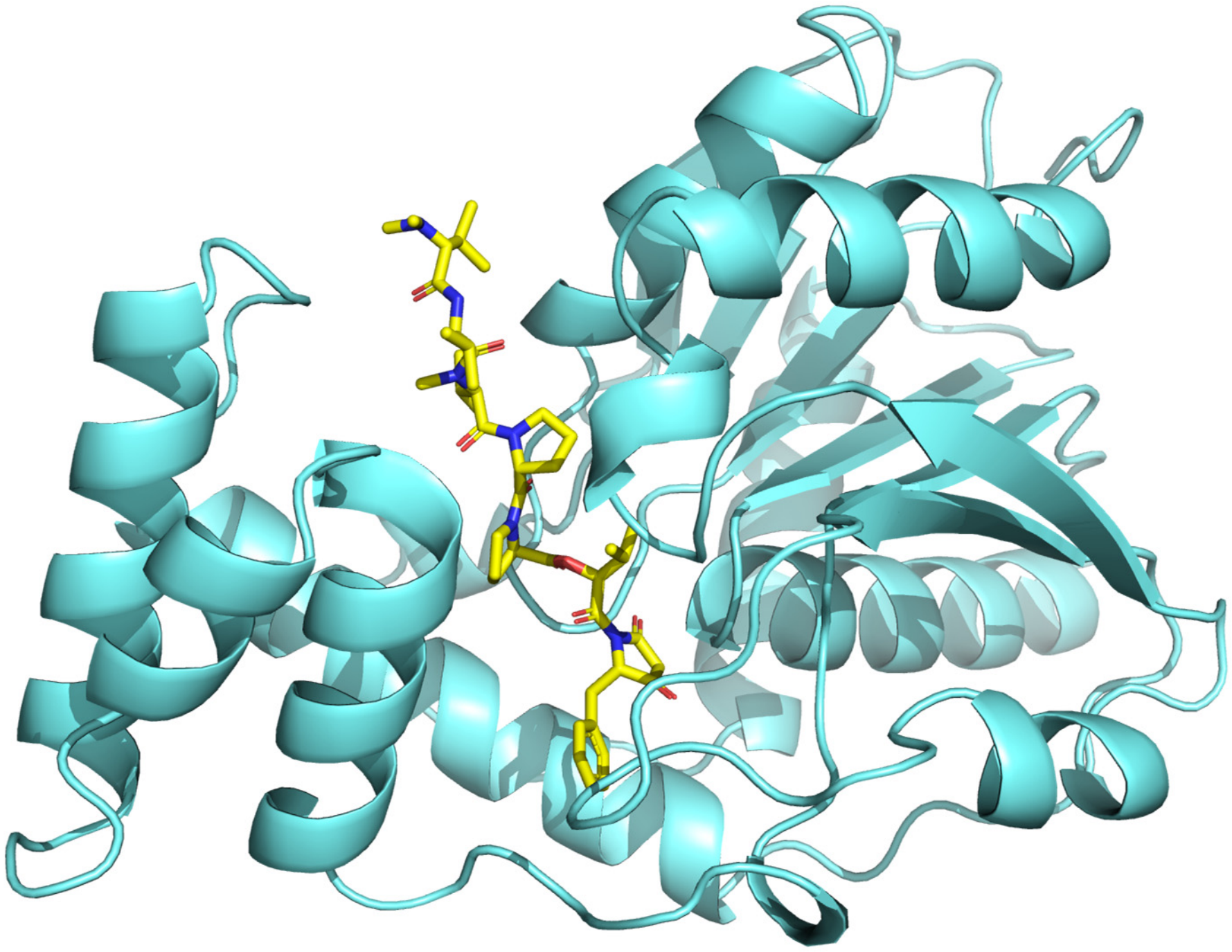

Fig. S27. Structure of Dol15F-TE binding with compound 2 predicted by AlphaFold 3. pTM = 0.94; ipTM = 0.93; ranking score = 0.93

Tables

Table S1. AntiSMASH BGC prediction of *Dapis* sp. VPG23-77.

BGC#	Class	Most similar known cluster	Similarity Confidence
1	hserlactone		
2	azole-containing-RiPP		
3	Terpene-precursor		
4	NRP-metallophore, NRPS, T1PKS	vatiamide	Low
5	NRPS	puwainaphycin	Low
6	terpene		
7	RiPP-like		
8	RiPP-like		
9	RRE-containing		
10	redox-cofactor		
11	RRE-containing,lanthipeptide-class-v,spliceotide	PcpA	Low
12	NRPS	hectochlorin	Low
13	NRPS		
14	NRPS	hexose-palythine-serine/hexose-shinorine	Low
15	T1PKS		
16	NRPS, T1PKS	nostopeptolide A2	Low
17	NRPS		
18	CDPS		

Table S2. Features of the gene product in the *dol15* BGC from *Dapis* sp. VPG23-77.

Protein	Size (AA)	Proposed function	Closest homolog in GenBank/RefSeq	I/S (%)
Dol15A	1048	NRPS [A-NMT-T]	NEP61017.1 [Symploca sp. SIO2G7]	83/91
Dol15B	2662	NRPS [C-A-T-C-A-NMT-T]	WP_293117522.1 [Moorena sp. SIO3E8]	65/76
Dol15C	1159	NRPS [C-A-T]	WP_293166106.1 [Okeania sp. SIO2C9]	82/90
Dol15D	3046	NRPS [C-A-T-C-A_x-KAL-KR-T]	WP_424101438.1 [Moorena producens]	79/86
Dol15E	1137	NRPS [C-A-T]	WP_293113631.1 [Moorena sp. SIO4G3]	84/90
Dol15F	1293	PKS [KS-AT-T-TE]	NEP50771.1 [Moorena sp. SIO3C2]	90/95
Dol15G	335	*O*-methyltransferase	NEP51059.1 [Moorena sp. SIO3C2]	85/90

*I/S: identity and similarity.

Table S3. Substrate prediction of the adenylation domains in the *dol15* BGC.

Domain	Proposed substrate	Amino acid residues*								
		235	236	239	278	299	301	322	330	331
Dol15A-A	Val	D	A	F	W	L	G	G	T	F
Dol15B-A1	Val	D	A	W	W	L	G	G	T	F
Dol15B-A2	Val	D	A	W	W	L	G	G	T	F
Dol15C-A	Pro	D	V	Q	F	V	A	Q	V	V
Dol15D-A1	Pro	D	V	Q	F	V	A	Q	V	V
Dol15DM2-A_x	Unknown	E	V	L	H	L	S	F	G	E
Dol15DM2-KAL	KIV	V	G	V	W	L	A	A	S	A
Dol15E-A	Phe	D	A	W	T	V	A	A	V	C

*Numbering according to the position in GrsA.

Table S4. Data collection and refinement statistics of Dol15G.

	Dol15G with SAH (PDB: 10QU)
**Data collection**	
Wavelength (Å)	0.9197
Space group	C 1 2 1
Resolution range (Å)	49.76 - 2.651 (2.72 - 2.65)
Cell dimensions	
a, b, c (Å)	133.015 139.746 71.145
α, β, γ (°)	90 94.95 90
Total reflections	131820 (8729)
Unique reflections	71863 (5120)
Multiplicity	1.8 (1.7)
Completeness (%)	99.75 (99.06)
Mean I/sigma(I)	5.90 (1.68)
Wilson B-factor	43.16
R-merge	0.08413 (0.4063)
R-meas	0.1165 (0.5603)
R-pim	0.08023 (0.3843)
CC1/2	0.988 (0.779)
**Refinement**	
Reflections used in refinement	37470 (2642)
Reflections used for R-free	1996 (137)
R-work	0.1740 (0.2766)
R-free	0.2191 (0.3168)
Number of non-hydrogen atoms	5594
macromolecules	5240
ligands	16
solvent	338
Protein residues	669
Nucleic acid bases	
RMS(bonds)	0.008
RMS(angles)	0.91
Ramachandran favored (%)	97.29
Ramachandran allowed (%)	2.71
Ramachandran outliers (%)	0
Rotamer outliers (%)	2.14
Clashscore	3.97
Average B-factor	46.06
macromolecules	45.91
ligands	47.86
solvent	47.95

Table S5. Primers and synthesized genes used in this study.

Name	Sequence (5'-3')
28b-KAL-Fw	TGCCGCGCGGCAGCCATATGGTCCCGGAGATAGAAGAAG
28b-KAL-Rv	GTGGTGGTGGTGCTCGAGCTACGAGTTCTCTGTGATCAGG
V219D-Fw	TAGATCAC**GAT**GGTGGCATC
V219D-Rv	GATGCCACC**ATC**GTGATCTA
Codon optimized gene of dol15D-Ax	CATATGACGCAAGAAGAACTGGATCAGATCCTTGTTGAATGGAATAACACTAAGACCGACT<br>ACCCTACGGATAAGTGCATACACCAACTGTTCGAAGAGCAAGTTGAAAAGAATCCGGACG<br>CTATTGCTGTCGTCTTCGAGGAACAGAAATTAACCTATTCCGAGCTGAACTCCAAGGCGA<br>ACCAGTTAGCTCACTACCTGCAGAAGTTAGCTTTTAAACCTGAGATTCAAGTGGGGATATA<br>CGTTGAACGGTCAGTCGAAATGATTATCGCCATTCTTGCGATTTTGAAGGCTGGGGCGGC<br>GTACTTGCCGATAGACCCTCAGGACAAGAGCCAAGACTTACAAAGTATCAGTGTGATTCT<br>CACACAGAATCATTTGAAGTCCAAGATCCCTGATAGCGAGGCCGAGATCTTGTGCCTTGA<br>CAAAGACTGGGAGAGTATTGCAAAGCAAAACACTGATAATCCGAATACCGGTACGACGCC<br>GACAAAGTTAACATATATCTTAAACCAGACGCTGGTGGAGCACCACGCCGTCGCCCAGC<br>GATTGCAGTGGCTGCAGGAGATACTCTCTCTGACAAACCAGGATATAGTGCTGCACAAGA<br>CATCACTGTCCGAAGAGGTCGCACTCCTGGAGATCGGATTGCCCTTGATTTCTGGCGGC<br>TCCATCGTGATCGCGGGTAACGATAATCCAACCGAGCTGCAGAAGTTGATCGCACAGCAT<br>AAGGTGACAATCGTGCATTTATACCCGTCCGAGTTGGCTACTTGGCTTAATACCACGAATC<br>AGGTTGCTAGTTTTAATTCGTGGCATACGTTATTATGTTCAGGGGAGACACTCAGCACCGA<br>GCTGGCGAATAAGTTCTTGCAGTCTTATCCTGTGAGCCTGCATAACTTCTACTCCATCCCT<br>GAGACGGCAGGGGAGATTACCCATTGGCAATGGCTGGAGAAGCCGACCCGTGAGAATG<br>TCCCGGTCGGAAATCCCGGCCGCTTGAGTGTGTACCTGTTGGATCAGCATCAGAATCCC<br>GTGCCGAAGGGCGTGCCGGGTGAGATTTACGTAGGCGGCAGCTCCCTGGCACGCGGTT<br>ACGTTCAGCAGCAACAGCAAACTTCGCAAGAGTTCATCGAGCATCCGCAGCTGGGGCG<br>CCTGTTTCAGACTGGGGATATTGGACGGTACCACAATCTGGGGTACCTGGAGATTCTGG<br>GGACGAAGCAGCGTCACACCTGGATCAAGGGTAAACGTGTGCAGTTGGCGGATATTGAG<br>ACCGCGTTGCTGTCTGCCCCGGGTGTGGAGCAGGCGTACATTCTTGCGCACCAGACTC<br>TCCTTGTTGCCTACGTGGTCGTGGCGGGTGTTTGGAATCCTCAGCAGTTGCATAGCCAG<br>ATCCAGCAACAATTTCCGCCCGATATGATGCCAGTTGCACTGGTACCGGTAAGTCGACTG<br>CCATTGACACATAAGGGCAAGGTTGATGAGGTTGCTCTTGGTAGATTTCCTGTCATCGAT<br>GACAATGTCGTTCAGCAGTGGGAAGCGAAGCTTAAGGCGTAGCTCGAG
Codon optimized gene of dol15D-KAL	CATATGGTCCCGGAGATAGAAGAAGTTGCAGTCGTCGTTCAGCAAAAGACCCTCAAGCT<br>GCCACCGCTGCACATCTCTGATTTATTACCGTCTGATAAGATCAAGTTGCCGAACAACGG<br>CGCTACGACAGTGGTGGAGAAATCCAGTCCCACTACGGAGCTGCAGACACAGTCTACCT<br>CTCGTAAACCTGCAATATCCGAGGGCGAACCGATTAAATGTCCTAAGGACGCTCCCACTA<br>CGCTGGCTCAGACCCTGGAGCGTGCGGCGCAGAAATATGCGGACTCCTCATTGATTTAC<br>ATACAAAGTGACGGAGAAGTTATAACGCAGACGTACCGTGACCTGTGGGTTGAGGCACA<br>GCGCATTCTGGGTGGTCTGCGCCAACTGGGCCTGAAGCCGCAGGATAAGGTGATTTTCC<br>AACTTGAGTCCAACCAGGATTTCATATCTGCCTTCTGGGGATGCGTCTTGGGTGGTTTCG<br>TGCCTGTTCCCGTTAGTATTCCGCCGAGCTACGATCAGTCGCATAGCACGTTAACTAAGC<br>TCCAGAACACGTGGCAGATGCTGGGCCAACCGCTGGTCCTTACTGACTCGAAGCTTGC<br>GCCTTCAGTACGAGGCTGGTCACAGCGATTAAACCTGGGAAAATTCGAATTGCAGATAAT<br>CGACAAGCTGCGGGAGTCTGAGCCGGAGTCTGAACTGCACTCTAGTCAGCCGGAAGAC<br>GTTGCCATCCTGCTGCTGACCAGTGGGTCTACCGGGATGCCCAAGGCAGTGATGCAATC<br>GCACTCGTCACTGCTGTCACGCAGCGCTGCGACCGCCCAAATCAATAACTTCACGTACG<br>AGGACATAAGCCTGAACTGGTTCCCGTTAGATCAC**GTG**GGTGGCATCGTCATGTTCCACA<br>TCAGAGACGTCTACCTTGGTTGCCAACAGATCCACGTTCCTACCCAAGAAATTCTCCAGG<br>CCCCCACGCGTTGGCTCGACCTGATGAGCCAATACGGCACCACGATAACCTGGGCGCC<br>GAATTTCGCCTATGGGCTTATCATAGATCAACTGGAGCAGCTGGATAAGATTGGCTCAGA<br>GAACTACCAGTGGGACTTGTCTAGTCTGAAGTTCATTCTTAACGCGGGCGAGGCTATTGT<br>GGCTAAGACAGCGCGTAAGTTTATGGAGTTCTTAGCGCGATATAACCTCCCTCCACAGGC<br>GATGCATCCGGCATGGGGCATGTCGGAGACAAGCAGTGCCGTCACATTTTCCAACAATTT<br>CTGGCTGGATAGCACCGACGACACCCAAAAGTTCGTTGAGGTCGGATCGCCAATACCTG<br>GATTCGCAATACGCATCGTAGACAACCAGAATCAAATTGTGAGTGAGGAGACCATCGGCC<br>GCGTCCAGGTCAAGGGTGCTTCGGTCACGAGTGGCTATTACCAGAACCCAACCGCCAAC<br>CAGGAAGCGTTCACAGCCGACGGATGGTTCAACACTGGTGACCTGGGATTCCTGAAGAA<br>TGGGTCGCTGACGATCACGGGGCGACAGAAGGATGTCATCATCATTAACGGACTGAATTA<br>TTATTCTCACGAGATAGAGTCCGTAGTGGAAGAGCTGCCGGAGATTGAGGTGTCGTATAC<br>TGCGGCGTGCGCGGTTCGTGAGACAAATAGTAACACCGACAAGCTGGGAATTTTCTTTAA<br>CTCAAGCAAGACTGAGGACACTGAACTGCTGGGACTCCTGAAGACACTGCGCGAGCAA<br>GTCGTAAATCGGATGGGCATCAACCCCGACTACCTTATCCCACTGGATAAGGATTTGATAC<br>CTAAGACCAGTATTGGGAAGATACAGCGCACCCAGTTATCACAGCGGTTCGCGAAGGGT<br>GAGTTTCAAGAGGTTCTGAAGAGAGTTGACCTGATCACAGAGAACTCGTAGCTCGAG
dol15G-Fw	CAGCCATATGTCATCATCACTAATTGC
dol15G-Rv	GGTGCTCGAGTCAAGCAGGTATCGCTTCTA
dol15F-TE-Fw	CAGCCATATGGTGTTTCCCGATTCAATAG
dol15F-TE-Rv	GGTGCTCGAGTCACCCCACAATATCTTGG

Dataset (separate file). (**S1**) Genomic sequencing and genome assembly statistics of four new Dapis strains. (**S2**). Genomic features of Dapis strains and cyanobacteria references used in phylogenomic analysis (Figure 2A). To maintain consistency across the dataset and eliminate potential bias from different annotation pipeline, all genomes were annotated using Prokka for this analysis. (**S3**) Orthogroup analysis of five *Dapis* strains and *Trichodesminum erythraeum* IMS101. Numbers indicate gene counts in each orthogroup category.

SI References

1. G. R. Pettit, *et al.*, Isolation and structure of the cytostatic linear depsipeptide dolastatin 15. *J. Org. Chem.* **54**, 6005–6006 (1989).
2. R. Ratnayake, *et al.*, Dolastatin 15 from a marine cyanobacterium suppresses HIF-1α mediated cancer cell viability and vascularization. *ChemBioChem* **21**, 2356–2366 (2020).
3. M. Kolmogorov, *et al.*, metaFlye: scalable long-read metagenome assembly using repeat graphs. *Nat. Methods* **17**, 1103–1110 (2020).
4. S. Nurk, *et al.*, HiCanu: accurate assembly of segmental duplications, satellites, and allelic variants from high-fidelity long reads. *Genome Res.* **30**, 1291–1305 (2020).
5. D. M. Portik, *et al.*, Highly accurate metagenome-assembled genomes from human gut microbiota using long-read assembly, binning, and consolidation methods. *bioRxiv* (2024). doi: https://doi.org/10.1101/2024.05.10.593587
6. A. Chklovski, D. H. Parks, B. J. Woodcroft, G. W. Tyson, CheckM2: a rapid, scalable and accurate tool for assessing microbial genome quality using machine learning. *Nat. Methods* **20**, 1203–1212 (2023).
7. K. Blin, *et al.*, antiSMASH 8.0: extended gene cluster detection capabilities and analyses of chemistry, enzymology, and regulation. *Nucleic Acids Res.* **53**, W32–W38 (2025).
8. B. J. Walker, *et al.*, Pilon: an integrated tool for comprehensive microbial variant detection and genome assembly improvement. *PLoS One* **9**, e112963 (2014).
9. K. Katoh, J. Rozewicki, K. D. Yamada, MAFFT online service: multiple sequence alignment, interactive sequence choice and visualization. *Brief. Bioinform.* **20**, 1160–1166 (2019).
10. A. Stamatakis, RAxML version 8: a tool for phylogenetic analysis and post-analysis of large phylogenies. *Bioinformatics* **30**, 1312–1313 (2014).
11. T. Seemann, Prokka: rapid prokaryotic genome annotation. *Bioinformatics* **30**, 2068–2069 (2014).
12. D. M. Emms, S. Kelly, OrthoFinder: phylogenetic orthology inference for comparative genomics. *Genome Biol.* **20**, 238 (2019).
13. D. Darriba, *et al.*, ModelTest-NG: a new and scalable tool for the selection of DNA and protein evolutionary models. *Mol. Biol. Evol.* **37**, 291–294 (2020).
14. A. M. Kozlov, D. Darriba, T. Flouri, B. Morel, A. Stamatakis, RAxML-NG: a fast, scalable and user-friendly tool for maximum likelihood phylogenetic inference. *Bioinformatics* **35**, 4453–4455 (2019).
15. P.-A. Chaumeil, A. J. Mussig, P. Hugenholtz, D. H. Parks, GTDB-Tk: a toolkit to classify genomes with the Genome Taxonomy Database. *Bioinformatics* **36**, 1925–1927 (2020).
16. Z. Xie, H. Tang, ISEScan: automated identification of insertion sequence elements in prokaryotic genomes. *Bioinformatics* **33**, 3340–3347 (2017).
17. J. Russel, R. Pinilla-Redondo, D. Mayo-Muñoz, S. A. Shah, S. J. Sørensen, CRISPRCasTyper: automated identification, annotation, and classification of CRISPR-Cas loci. *CRISPR J.* **3**, 462–469 (2020).
18. I. Letunic, P. Bork, Interactive Tree Of Life (iTOL) v5: an online tool for phylogenetic tree display and annotation. *Nucleic Acids Res.* **49**, W293–W296 (2021).
19. S. R. Eddy, Accelerated profile HMM searches. *PLoS Comput. Biol.* **7**, e1002195 (2011).
20. I. Lee, Y. Ouk Kim, S.-C. Park, J. Chun, OrthoANI: An improved algorithm and software for calculating average nucleotide identity. *Int. J. Syst. Evol. Microbiol.* **66**, 1100–1103 (2016).
21. S. Kurtz, *et al.*, Versatile and open software for comparing large genomes. *Genome Biol.* **5**, R12 (2004).

22. M. Krzywinski, *et al.*, Circos: An information aesthetic for comparative genomics. *Genome Res.* **19**, 1639–1645 (2009).
23. A. Draisma, *et al.*, BiG-SCAPE 2.0 and BiG-SLiCE 2.0: scalable, accurate and interactive sequence clustering of metabolic gene clusters. *Nat. Commun.* **17**, 2000 (2026).
24. P. Shannon, *et al.*, Cytoscape: A software environment for integrated models of biomolecular interaction networks. *Genome Res.* **13**, 2498–2504 (2003).
25. C. L. M. Gilchrist, Y.-H. Chooi, Clinker & clustermap.js: automatic generation of gene cluster comparison figures. *Bioinformatics* **37**, 2473–2475 (2021).
26. B. R. Terlouw, *et al.*, PARAS: high-accuracy machine-learning of substrate specificities in nonribosomal peptide synthetases. *JACS Au*. 6: 2315-2336 (2026).
27. S. Capella-Gutiérrez, J. M. Silla-Martínez, T. Gabaldón, trimAl: a tool for automated alignment trimming in large-scale phylogenetic analyses. *Bioinformatics* **25**, 1972–1973 (2009).
28. L.-T. Nguyen, H. A. Schmidt, A. von Haeseler, B. Q. Minh, IQ-TREE: a fast and effective stochastic algorithm for estimating maximum-likelihood phylogenies. *Mol. Biol. Evol.* **32**, 268–274 (2015).
29. S. Kalyaanamoorthy, B. Q. Minh, T. K. F. Wong, A. von Haeseler, L. S. Jermiin, ModelFinder: fast model selection for accurate phylogenetic estimates. *Nat. Methods* **14**, 587–589 (2017).
30. B. Boussau, L. Guéguen, M. Gouy, A mixture model and a hidden markov model to simultaneously detect recombination breakpoints and reconstruct phylogenies. *Evol. Bioinform. Online* **5**, 67-79 (2009).
31. O. Gascuel, BIONJ: an improved version of the NJ algorithm based on a simple model of sequence data. *Mol. Biol. Evol.* **14**, 685–695 (1997).
32. J. Abramson, *et al.*, Accurate structure prediction of biomolecular interactions with AlphaFold 3. *Nature* **630**, 493–500 (2024).
33. M. M. Zdouc, *et al.*, MIBiG 4.0: advancing biosynthetic gene cluster curation through global collaboration. *Nucleic Acids Res.* **53**, D678–D690 (2025).
34. K. Blin, S. Shaw, M. H. Medema, T. Weber, The antiSMASH database version 5. *Nucleic Acids Res.* **54**, D522–D526 (2026).
35. Z. Lin, *et al.*, Evolutionary-scale prediction of atomic-level protein structure with a language model. *Science.* **379**, 1123–1130 (2023).
36. Dawei Ma, Jingyuan Ma, Wenli Ding, Lixin Dai, An improved procedure to homochiral cyclic statines. *Tetrahedron Asymm.* **7**, 2365–2370 (1996).
37. A. Murakami, *et al.*, Natural dolapyrrolidone: Isolation and absolute stereochemistry of a substructure of bioactive peptides. *Chirality* **32**, 1152–1159 (2020).
38. D. K. Schneider, *et al.*, AMX – the highly automated macromolecular crystallography (17-ID-1) beamline at the NSLS-II. *J. Synchrotron Radiat.* **29**, 1480–1494 (2022).
39. W. Kabsch, *XDS*. *Acta Crystallogr. D Biol. Crystallogr.* **66**, 125–132 (2010).
40. K. Cowtan, The *Buccaneer* software for automated model building. 1. Tracing protein chains. *Acta Crystallogr. D Biol. Crystallogr.* **62**, 1002–1011 (2006).
41. P. Emsley, B. Lohkamp, W. G. Scott, K. Cowtan, Features and development of *Coot*. *Acta Crystallogr. D Biol. Crystallogr.* **66**, 486–501 (2010).
42. P. D. Adams, *et al.*, *PHENIX*: a comprehensive Python-based system for macromolecular structure solution. *Acta Crystallogr. D Biol. Crystallogr.* **66**, 213–221 (2010).
43. G. N. Murshudov, *et al.*, *REFMAC* 5 for the refinement of macromolecular crystal structures. *Acta Crystallogr. D Biol. Crystallogr.* **67**, 355–367 (2011).
